# Deep Learning in Digital Breast Tomosynthesis: Current Status, Challenges, and Future Trends

**DOI:** 10.1002/mco2.70247

**Published:** 2025-06-09

**Authors:** Ruoyun Wang, Fanxuan Chen, Haoman Chen, Chenxing Lin, Jincen Shuai, Yutong Wu, Lixiang Ma, Xiaoqu Hu, Min Wu, Jin Wang, Qi Zhao, Jianwei Shuai, Jingye Pan

**Affiliations:** ^1^ Oujiang Laboratory (Zhejiang Lab for Regenerative Medicine, Vision, and Brain Health) Wenzhou Institute University of Chinese Academy of Sciences Wenzhou China; ^2^ Wenzhou Medical University Wenzhou China; ^3^ UCSC Baskin School of Engineering University of California Santa Cruz California USA; ^4^ Department of Anatomy Histology & Embryology School of Basic Medical Sciences Fudan University Shanghai China; ^5^ Department of Medicine Harvard Medical School and Brigham and Women's Hospital Boston Massachusetts USA; ^6^ Joint Research Centre on Medicine The Affiliated Xiangshan Hospital of Wenzhou Medical University Ningbo China; ^7^ Stony Brook University Stony Brook New York USA; ^8^ School of Computer Science and Software Engineering University of Science and Technology Liaoning Anshan China; ^9^ Key Laboratory of Intelligent Treatment and Life Support for Critical Diseases of Zhejiang Province Wenzhou China; ^10^ Department of Big Data in Health Science The First Affiliated Hospital of Wenzhou Medical University Wenzhou China; ^11^ Zhejiang Engineering Research Center for Hospital Emergency and Process Digitization Wenzhou China

**Keywords:** early breast cancer screening, digital breast tomosynthesis, deep learning, public database, medical image analysis

## Abstract

The high‐resolution three‐dimensional (3D) images generated with digital breast tomosynthesis (DBT) in the screening of breast cancer offer new possibilities for early disease diagnosis. Early detection is especially important as the incidence of breast cancer increases. However, DBT also presents challenges in terms of poorer results for dense breasts, increased false positive rates, slightly higher radiation doses, and increased reading times. Deep learning (DL) has been shown to effectively increase the processing efficiency and diagnostic accuracy of DBT images. This article reviews the application and outlook of DL in DBT‐based breast cancer screening. First, the fundamentals and challenges of DBT technology are introduced. The applications of DL in DBT are then grouped into three categories: diagnostic classification of breast diseases, lesion segmentation and detection, and medical image generation. Additionally, the current public databases for mammography are summarized in detail. Finally, this paper analyzes the main challenges in the application of DL techniques in DBT, such as the lack of public datasets and model training issues, and proposes possible directions for future research, including large language models, multisource domain transfer, and data augmentation, to encourage innovative applications of DL in medical imaging.

## Graphical abstract

Breast cancer detection involves a combination of clinical diagnosis, pathological examination, and various imaging tests. We focus on utilizing deep learning models in digital breast tomosynthesis for diagnostic classification, lesion localization, and medical image generation. This study addresses challenges such as data insufficiency, model overfitting, and low interpretability, along with proposed solutions for these issues in deep learning applications.

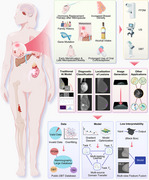

## 1 Introduction

Breast cancer has emerged as the leading malignancy threatening women's health worldwide [1, 2, 3]. According to the International Agency for Research on Cancer, by 2022, female breast cancer (11.6%) was the second most common cancer in the world [4]. Among women, breast cancer is the most common cancer and is the cause of cancer death, with the highest incidence in 157 countries/areas and the highest mortality in 112 countries/areas worldwide [5]. The high mortality and morbidity rates of breast cancer place a heavy burden on global health [6, 7].

The complexity of breast cancer lies in the diversity and dynamics of its somatic mutations and abnormalities in gene and protein expression profiles [8, 9]. These changes involve numerous genetic elements, including mutations and aberrant amplification of oncogenes, which collectively contribute to the onset and progression of breast cancer [10, 11, 12, 13, 14]. Additionally, the interplay among multiple risk factors, such as demographic variables such as sex and age, hormones such as estrogen, genetic predisposition, and modifiable lifestyle factors, further increases the risk of developing breast cancer [15, 16, 17, 18].

Therefore, early detection of breast cancer is crucial for reducing the disease burden, enhancing treatment effectiveness, and improving patient prognosis [19]. According to Cancer Research UK, the 5‐year survival rate for patients with stage 1 breast cancer is nearly 100%, whereas for stage 4 disease, it drops sharply to 25% [20]. These data underscore the critical role of early diagnosis in improving the outcomes and overall survival of patients with breast cancer. Common imaging modalities for breast cancer include ultrasonography, X‐ray, and breast tissue elastography. X‐ray‐based techniques include conventional mammography, digital mammography (DM), full‐field digital mammography (FFDM), and digital breast tomosynthesis (DBT) [21, 22, 23, 24]. While screening mammography is widely utilized globally, current mammography techniques have significant limitations, such as high false‐positive and false‐negative rates and variability in clinician proficiency, which can lead to potential delays and inaccuracies in diagnosis [25]. Additionally, while CT can provide detailed imaging, its clinical application in breast cancer screening and diagnosis remains limited.

**FIGURE 1:**
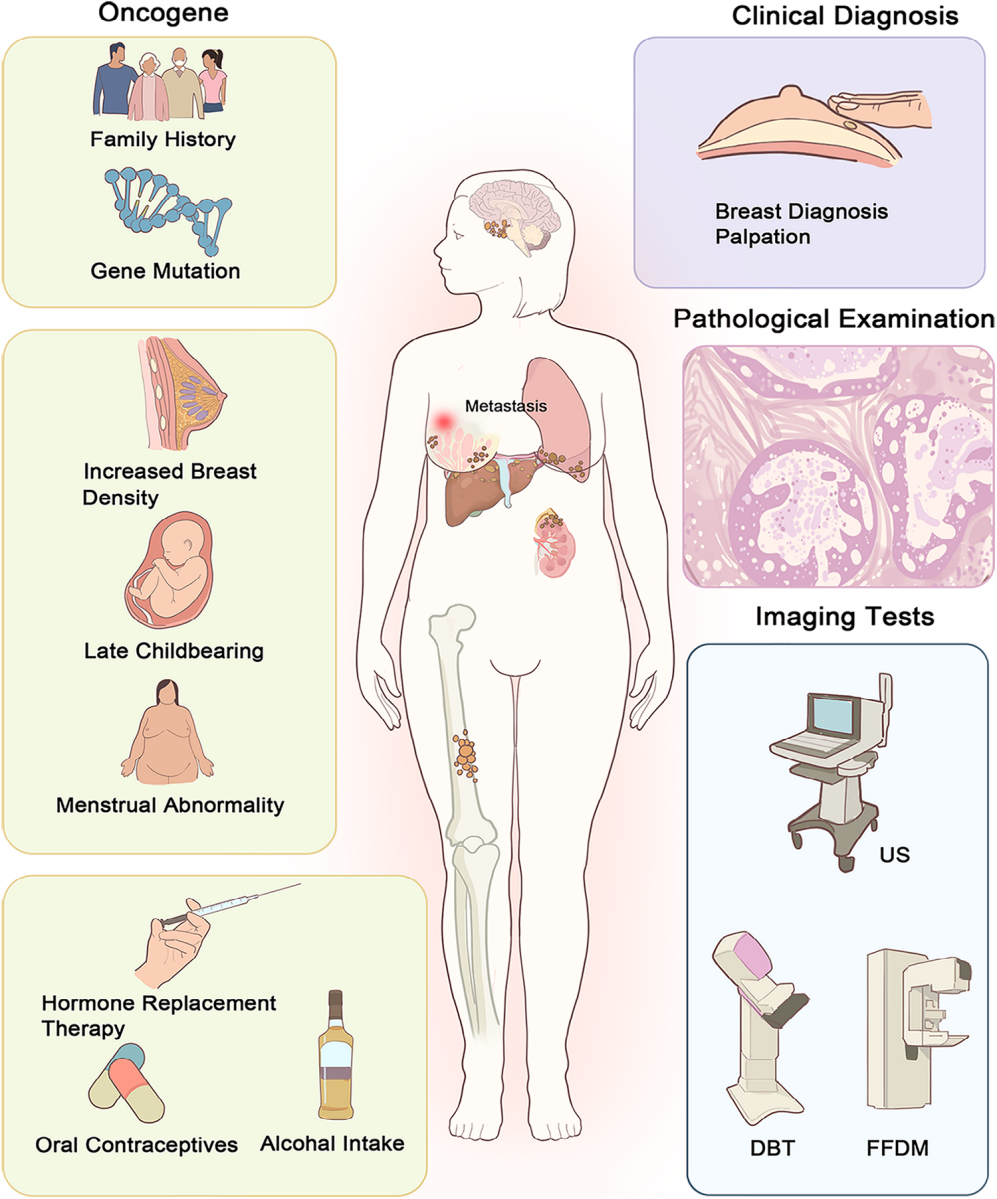
Breast cancer causes, pathogenesis, and screening methods. The causes of breast cancer include family history, gene mutations, increased breast density, late childbearing, menstrual abnormalities, hormone replacement therapy, oral contraceptives, and alcohol intake. The pathogenesis of breast cancer involves mutations in oncogenes within breast cells, leading to abnormal cell proliferation, tumor formation, and invasion of surrounding tissues. Over time, these cancer cells can break through the basement membrane and metastasize to distant sites via the lymphatic and circulatory systems. Screening methods for breast cancer include clinical diagnosis, pathological examination, and imaging tests such as US, FFDM, and DBT.

Compared with conventional mammography, DM offers superior image quality, faster processing, and more convenient storage [26]. The subsequently developed FFDM, which projects raw three‐dimensional (3D) breast tissue data and converts it into a two‐dimensional (2D) image, has become widely used in clinical settings because of its utility in early detection and intervention [27]. Compared with the DM, the FFDM provides a wider field of view, results in fewer repeat examination errors, and achieves higher detection rates [28]. Randomized controlled studies have shown that FFDM‐assisted screening can reduce the associated mortality rate with breast cancer by approximately 20% [29, 30]. However, FFDM images are 2D and have relatively low specificity [31, 32]. DBT offers superior detection rates, reduced interference from overlapping tissues, and improved accuracy in breast cancer screening with respect to FFDM (Figure 1) [26, 33].

DBT is an innovative advancement in breast imaging based on FFDM [34]. X‐ray receivers acquire a series of low‐dose X‐ray images at various angles around the breast [35, 36, 37]. These multiple 2D images are then reconstructed via a computer algorithm to produce a 3D volumetric image of the breast, enabling a more detailed view of the breast tissue and facilitating the detection of abnormalities such as lumps and calcifications [38, 39, 40]. The complex reconstruction algorithm minimizes the effects of tissue overlap and structural noise, significantly improving the visualization of lesion edges and thereby the diagnosis of breast cancer; in turn, this improved visualization markedly enhances the sensitivity of DBT [10]. Additionally, DBT reduces diagnostic errors associated with the inherent complexity of fibroglandular breast tissue, thus lowering the false‐negative rate [41, 42]. The advantages of DBT in terms of detection efficacy have led to it replacing DM as the imaging modality of choice over the past decade [30]. However, the large number of slices per patient who must be acquired to achieve 3D imaging relative to 2D mammography can influence the efficiency and accuracy of the physician responsible for diagnosing the patient [43].

The development of artificial intelligence (AI) has profoundly impacted the medical field; among the different forms of AI, deep learning (DL) technology has shown powerful automatic image feature extraction capabilities [44, 45, 46]. This technology can perform end‐to‐end feature learning and classification decision‐making directly from raw data, eliminating the need for manual feature selection [47, 48, 49]. DL has been widely used to improve the early detection and treatment of various types of cancers, leading to substantial increases in patients' chances of survival [50, 51, 52, 53]. Researchers have also applied DL to assist in the diagnosis of breast cancer, particularly in detecting and diagnosing lesions with DBT data, with the aim of improving the speed and accuracy of radiologists interpretations [30, 54‐56]. In recent years, rapid advances in the field of DL have improved the field of breast imaging, resulting in better performance than traditional mammography‐assisted detection methods [57]. However, the application of DL technology in the domain of DBT still faces numerous difficulties and challenges, indicating substantial room for improvement.

Numerous authors have reviewed the application of AI, particularly DL technology, in the field of DBT. For example, Geras et al. [58] introduced the limitations of classical computer‐aided detection systems in DBT data, demonstrating how DL systems can enhance the accuracy of malignant tumor detection through neural networks. However, their summary was neither comprehensive nor adequate they propose effective solutions [58]. In 2023, Yoon et al. [59] conducted a systematic review and meta‐analysis to comprehensively evaluate the effectiveness of AI technology in DM and DBT. Nevertheless, they did not include sufficient studies assessing the performance of AI systems in interpreting DBT screening examinations and they neglected the collection of publicly available data on mammography [59]. In 2021, Bai et al. [60] discussed the specific technical challenges and solutions faced by DL algorithms in processing DBT data. However, their discussion did not explore algorithmic interpretability, patient privacy protection, and legal and ethical issues in practical clinical applications [60]. In 2023, Zhang et al. [61] provided a comprehensive review of AI in breast cancer imaging analysis, covering aspects such as data augmentation, image segmentation, diagnosis, and prognosis assessment. However, their review lacked a detailed discussion of DL models in DBT, making it difficult to fully address their applicability for this imaging modality [61]. This review aims to provide a more comprehensive summary and advanced insights into the application of DL technology in DBT to help promote the development and application of AI in this field.

This work reviews the methods, challenges, and future directions related to the application of DL to DBT data for the early screening of breast cancer. The manuscript can be divided into four parts. First, a brief overview of the basic principles, advantages, and limitations of DBT is provided. Second, based on the application of DL in medical image processing and analysis of breast diseases, DL models are classified into three categories, and their applications in early breast cancer screening are summarized. Third, the application of DL in other areas of DBT, along with the application of other AI techniques in early DBT screening, including the provision of publicly accessible databases, is explored. Finally, the challenges of applying DL in DBT are presented, and future research directions are summarized.

## 2 DBT: Basic Knowledge and Importance

Commonly used mammography examinations in clinical practice include DM, FFDM, and DBT. The DM employs digital sensors to capture mammographic images; specifically, the acquired electronic data are transmitted to a computer for processing, producing a breast image [62, 63]. DM has played a key role in reducing breast cancer mortality rates but has faced criticism due to its high number of false‐positive results and limited sensitivity, and the potential for overdiagnosing clinically unimportant lesions [64]. FFDM captures X‐ray images of the entire breast area with digital sensors, allowing a wider field of view and resulting in a more accurate mammogram [65, 66].

FFDM has long been considered the gold standard for breast cancer screening [67, 68]. However, it exhibits reduced sensitivity and specificity when affected by the “masking effects” of the upper parenchyma mal tissues [69]. These limitations are addressed in DBT through the arc motion of the X‐ray generator above the patient, resulting in the emission of low‐dose X‐rays at predetermined intervals from different angles and allowing the capture of images from various perspectives [70]. After acquisition, the DBT images are reconstructed into slices as thin as 1 mm in a plane parallel to the detector, and 3D reconstruction is performed to help determine the position of the lesion relative to the breast tissue [71, 72].

Studies have shown that for breast lesions, DBT provides better visibility of the mass, areas of structural distortion, and margin characterization [73, 74]. Researchers have shown that models trained on 3D DBT data can help reduce recall rates, improve biopsy patient selection, and increase cancer detection rates, particularly for patients with dense breasts [75, 76, 77]. Therefore, DBT is more advantageous for breast cancer screening and diagnosis than DM and FFDM are, facilitating early treatment and prognostic assessment of the disease [78].

Despite the many advantages of DBT over conventional mammography, there are some limitations in integrating this emerging technology into routine clinical practice. DBT, which functions as a pseudotomographic imaging modality, produces a series of 2D slices of the target breast tissue at varying vertical resolutions. This partial tomosynthesis effect significantly reduces the masking effect caused by overlapping tissue and is less effective for women with very dense breasts [79, 80, 81]. During screening, the radiosensitivity of the female breast requires careful attention to the radiation dose [82, 83, 84]. Barufaldi et al. [85] conducted an empirical assessment using a specialized tracking system to monitor the radiation doses from the DM and DBT. Their findings indicated a statistically discernible increase in the radiation dose associated with DBT in comparison with that associated with DM (2.21 vs. 1.76 mGy, respectively). While the radiation dose from DBT is marginally higher than that from DM, it remains within the acceptable limits prescribed by the Mammography Quality Standards Act [85].

**FIGURE 2:**
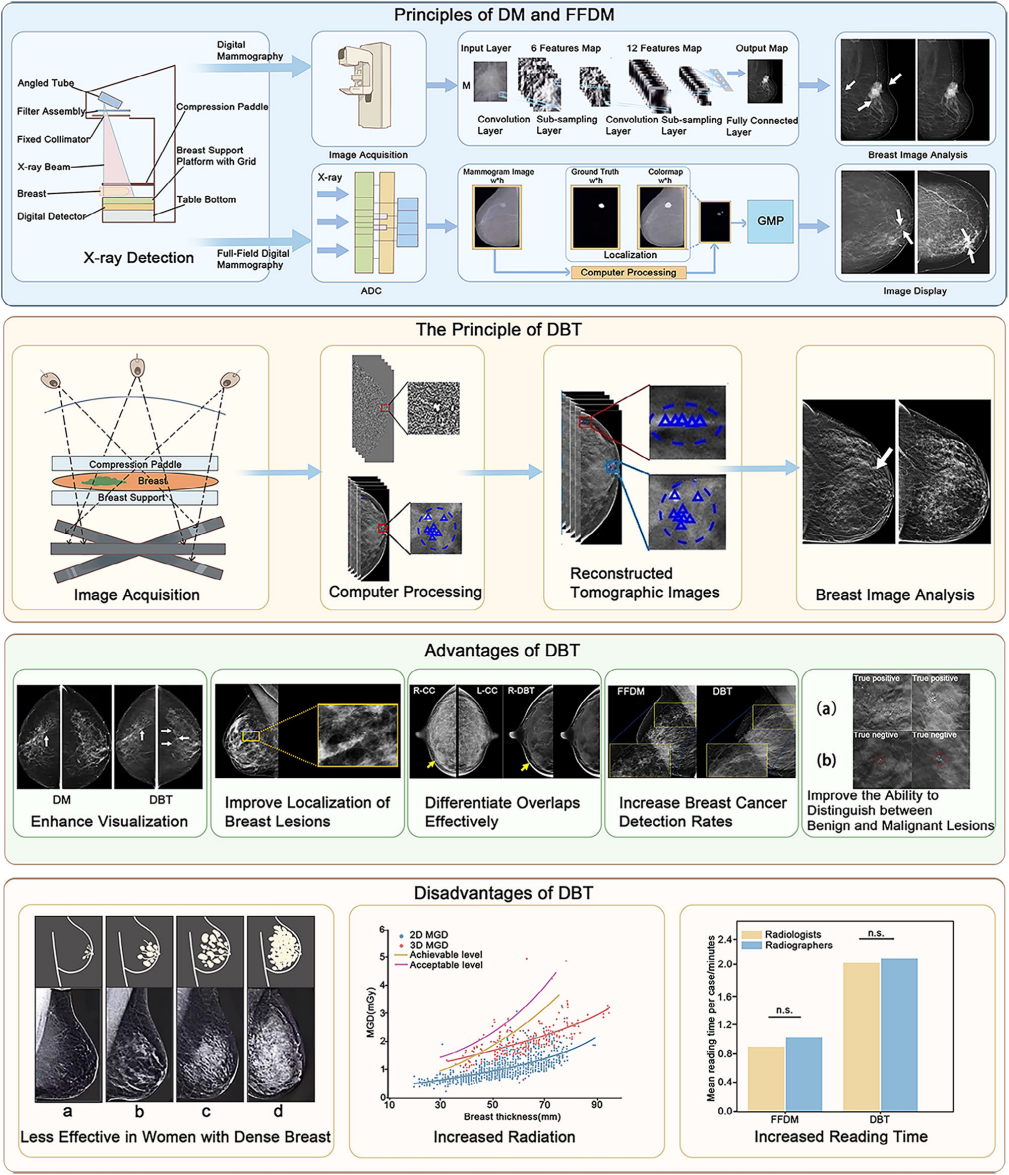
Principles of DM, FFDM, and DBT imaging and the advantages and disadvantages of DBT in clinical applications. DM uses a digital detector to penetrate the breast tissue with X‐rays. The sensor then converts X‐ray absorption into a digital signal that is then processed by a computer to produce a high‐resolution image of the breast. This is achieved through the use of image filtering, feature extraction, and enhancement. FFDM uses a planar detector and a rotating arm to comprehensively scan the breast tissue area, convert the X‐ray signals to digital form, and capture high‐resolution images of the internal structure of the breast via a fully digital detector. The data are then processed and reconstructed by a computer to produce high‐resolution breast images. DBT is based on the imaging principle of obtaining a series of high‐resolution, 2D breast images by taking multiple, low‐dose exposures of an X‐ray tube over a limited angular range. The images are then reconstructed by a computer into high‐resolution tomograms parallel to the detector, resulting in 3D tomograms. DBT has several advantages over DM and FFDM, including enhanced visualization, precise localization, effective differentiation of overlap, improved detection of breast cancer, and improved discrimination between benign and malignant lesions. However, DBT also has some disadvantages, such as being less effective in women with dense breasts, higher radiation dose [396], and longer reading time [87], which can impact efficiency. MGD, mean glandular dose.

In addition, the workload is increased by the complexity of DBT images and the long interpretation time, which leads to the need for double reading for many screening procedures, further exacerbating the workload [43, 86, 87]. DBT also faces challenges in the detection of calcifications and the need for breast compression during the imaging process, and the long compression time may affect patient tolerance [88] (Figure 2). Therefore, a comprehensive review of the use of computational modeling in DBT to accelerate the diagnostic process is of particular importance.

## 3 DL in DBT for the Early Screening of Breast Cancer

Developments in AI have led to its implementation in medical contexts by an increasing number of people to improve the efficiency and accuracy of diagnosis [43, 89‐91]. DL plays an important role in the clinical diagnosis and treatment of diseases, covering the fields of serology, imaging, and genomics. In 2023, Chi et al. [92] identified specific pancreatic cancer biomarkers by analyzing serum miRNA expression profiles from the GEO database via machine learning and artificial neural networks and developed a novel artificial neural network model for early diagnosis. Li et al. [93] developed and validated a two‐stage DL model based on multichannel MRI for automatic detection and segmentation of brain metastases, which excelled in improving the detection sensitivity and segmentation accuracy of tumors smaller than 5 mm, achieving 90% sensitivity and 56% accuracy on a test set. In 2024, Hoang et al. [94] developed the ENLIGHT‐DeepPT framework, which effectively predicts cancer treatment outcomes from pathology images with an overall advantage ratio of 2.28 and an accuracy of 46.5%.

DBT typically requires nearly twice the acquisition and interpretation time of DM, thus increasing the workload of the imaging physician [90]. AI algorithms have been shown to enhance the ability of the imaging physician to recognize cancers in mammograms without prolonging the reading time. Over the past decade, DL‐based computer‐aided diagnostic (CAD) systems have achieved remarkable success in medical image diagnostics, especially in early screening tasks involving DBT [95, 96, 97, 98]. DL techniques have also demonstrated broad application prospects in other medical image analysis fields, such as disease classification [99], segmentation [100, 101], detection [102], and image alignment [103].

### 3.1 Diagnostic Classification of Breast Diseases From DBT Data via DL

DL has a wide range of applications in different stages of DBT, including image reconstruction [104], improving image quality [105], and reducing noise. Over the years, several DL‐based methods have been developed for performing classification tasks. In the early days, perceptron and multilayer perceptron (MLP) methods were developed to address linear and nonlinear problems [106, 107]. In the 1990s, Yann LeCun's [108] LeNet‐5 demonstrated the potential utility of convolutional neural networks (CNNs). The success of AlexNet in 2012 marked the widespread adoption of DL. Subsequently, a visual geometry group network was developed, enhancing classification performance through increased network depth [109, 110, 111, 112]. Google neural networks subsequently introduced the inception module to improve efficiency [113, 114], and a residual network (ResNet) resolved the gradient vanishing problem in deep networks through residual connectivity [115, 116]. In recent years, an efficient neural network (EfficientNet) has been developed even higher efficiency and accuracy through composite scaling [117, 118, 119], whereas the vision transformer has demonstrated the competitiveness of transformer‐based architectures in image classification tasks [120, 121, 122]. Continued innovations in these models have driven significant performance improvements in classification tasks and facilitated the application of DL in other domains.

**FIGURE 3:**
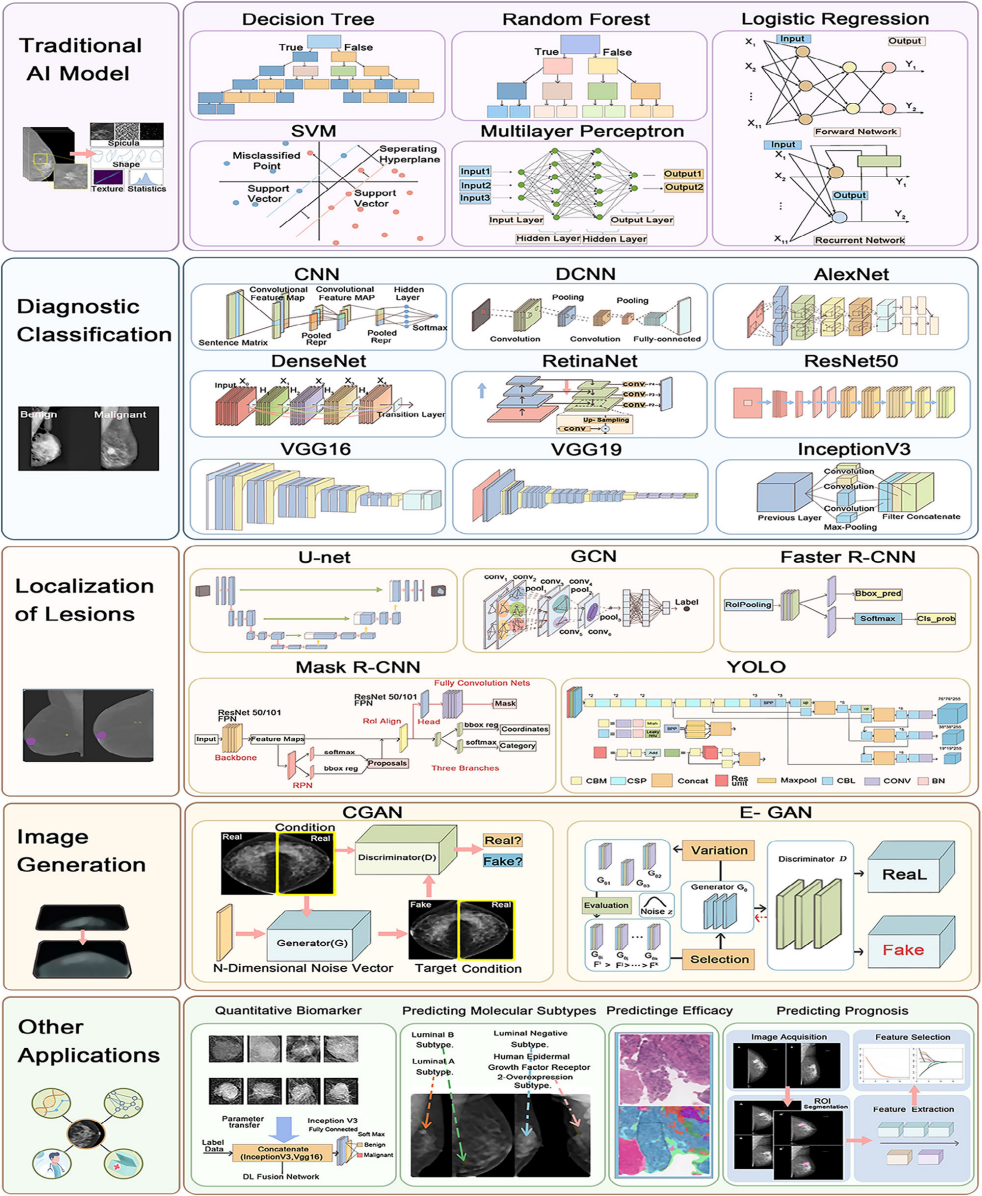
Applying DL models and other AI techniques to DBT data. The traditional AI models applied to DBT include decision trees, random forests, logistic regression, SVM, and multilayer perceptron. Deep learning models for the diagnostic classification of breast diseases on DBT data include CNN, DCNN, AlexNet, DenseNet, RetinaNet, ResNet50, VGG16, VGG19, and Inception V3. The deep learning models used for breast lesion segmentation and detection on DBT data include U‐Net and GCN. Other DL models used for medical image generation from DBT data include Faster R‐CNN, Mask R‐CNN, and YOLO. In addition, a GAN has been used for this purpose. The applications of DL in DBT go beyond early detection and diagnosis of breast cancer. It can also act as a quantitative biomarker, predict molecular subtypes, assess treatment efficacy, and predict patient prognosis.

However, most well‐established DL models originally designed to analyze natural images may produce suboptimal results when applied directly to medical imaging tasks [123], in part due to the substantial differences between the two types of images. Additionally, DBT data differ notably from ordinary medical imaging data [124]. For example, in the application of case of DBT data to breast cancer screening, 3D imaging is used to provide higher tissue resolution and contrast via the acquisition of a series of low‐dose X‐ray images from different angles and their reconstruction into 3D images [125, 126]. While this method reduces the effect of tissue overlap and improves lesion detection, it also introduces a series of challenges [127, 128]. Lesions may appear as different shapes and with different densities in different slices, and the complexity of normal tissues further increases lesion identification difficult [129]. Therefore, DBT data hold great research importance for the classification of breast diseases. Next, we detail the application of various DL models in DBT (Figure 3).

#### 3.1.1 Convolutional Neural Network

CNN extract local features from data through convolutional operations, construct high‐level semantic information layer by layer, and are commonly used to process structured data such as images. In 2019, Samala et al. developed a CNN using multistage transfer learning (MSTL) for classifying malignant and benign masses on DBT images. They first fine‐tuned an ImageNet‐trained CNN using more readily available mammography data and then performed a second stage of fine‐tuning using a small, available DBT dataset. This article employs a MSTL approach to feature extraction, in particular applying pretrained knowledge of CNNs in relevant ancillary domains to the task of classifying breast cancer. They compared multistage fine‐tuned CNNs with single‐stage CNNs fine‐tuned directly with DBT data and assessed how the different fine‐tuning strategies affected the performance of the CNNs when the available mammography and DBT data varied over a wide range. Additionally, the impact of different migration learning strategies on classification performance was evaluated via statistical methods with restricted sample sizes, such as area under the curve (AUC) and paired *t*‐tests. In the single‐stage transfer learning (STRL) test set, the view‐based AUC was 0.85 ± 0.05, whereas in the MSTL test set, the AUC significantly improved to 0.91 ± 0.03. The study demonstrated that when the target domain has a limited number of training samples, using data from similar auxiliary domains for additional stages of training is effective [130].

In 2021, Aswiga et al. [131] developed an innovative, two‐tier framework for classifying the data in DBT datasets. Initially, they built a basic framework based on multilevel transfer learning, whose main goal was to utilize knowledge gained from five other datasets (including a general nonmedical imaging dataset and a specialized mammography dataset) for feature extraction and subsequent classification of the target DBT dataset. By progressively fine‐tuning the model, they enhanced the classification performance. Notably, the AUC for the fine‐tuned MedCNN using the CNCF fusion algorithm reached an impressive value of 0.89.

#### 3.1.2 Deep Convolutional Neural Network

The deep CNN (DCNN) is based on CNNs and is suitable for more complex tasks by increasing the depth and number of layers of the network to improve the feature extraction and representation capabilities of the model. In 2018, Samala et al. [132] proposed a layered pathway evolution method to compare multiple DCNNs for breast cancer classification via DBT. Initially, transfer learning was employed with 19,632 augmented regions of interest (ROIs) from 2454 lesions identified on mammograms to further train a DCNN pretrained on ImageNet. Subsequently, 9120 DBT ROIs from 228 lesions were used in the second stage to further train the pretrained DCNN. The results demonstrated substantial network simplification; the number of neurons decreased by 87%, the number of parameters was reduced by 34%, and the required multiplications and additions in the convolutional layer were diminished by 95%. The AUC in the test increased from 0.88 to 0.90 in the evaluation of 89 lesions identified on mammograms from 94 independent DBT cases before and after pruning, respectively. The results of that study suggest that the features learned by a DCNN from mammography can be effectively transferred to DBT.

#### 3.1.3 Graph Convolutional Network

The graph convolutional network (GCN) is a neural network for graph‐structured data that extracts node features and captures node relationships through neighborhood aggregation. In 2018, Zhang et al. [133] developed a novel method called boundary‐aware dense region CNN with a GCN (BDR–CNN–GCN), which integrates two state‐of‐the‐art neural networks. This study used a combination of a CNN and a GCN through feature extraction to increase the performance of breast cancer detection, and the performance was evaluated via statistical metrics such as sensitivity, specificity, and accuracy. The experimental results indicate that compared with the five proposed neural network models and 15 state‐of‐the‐art breast cancer detection methods, the BDR–CNN–GCN method achieves superior performance, with a sensitivity of 96.20 ± 2.90%, a specificity of 96.00 ± 2.31%, and an accuracy of 96.10 ± 1.60%. These findings demonstrate that BDR–CNN–GCN is an effective method for improving malignant breast mass detection. However, importantly, both the training and test sets in that study were derived from the Mini‐Mammographic Image Analysis Society dataset, and no independent external validation was conducted. Therefore, the reliability of the model requires further verification. Future studies are planned to test the proposed AI model by expanding the dataset to include mammography X‐ray images from diverse sources and with varying resolutions.

#### 3.1.4 Dense Convolutional Network Model

Dense convolutional network (DenseNet) enhances feature reuse and improves training efficiency by directly using the output of each layer as the input for all subsequent layers. In 2022, Pawar et al. [134] proposed a multichannel DenseNet architecture for breast cancer detection. This architecture consists of a four‐channel transfer learning architecture that extracts important features from two medial oblique views and two craniocaudal views of digital mammograms of a single patient. An evaluation of 800 digital mammograms with different breast imaging reporting and data system (BI‐RADS) density levels revealed that the architecture achieved statistically good performance: 96.67% accuracy in the training set and 90.06% accuracy in the test set, with a mean AUC of 0.9625. These findings suggest that the proposed architecture can achieve state‐of‐the‐art results with a relatively small number of images and low computational power. Additionally, DenseNet enhances the propagation of features throughout the network and facilitates feature reuse, significantly reducing the number of parameters needed and thus simplifying the network's computational requirements. Compared with most advanced network architectures, the DenseNet architecture yields superior breast cancer detection models with less computational effort.

#### 3.1.5 RetinaNet Model

RetinaNet enhances the detection of small and challenging objects by introducing focal loss, which balances positive and negative samples while maintaining both efficiency and accuracy. In 2023, Habeeb et al. [135] achieved efficient breast cancer detection with a two‐stage transfer learning approach combined with RetinaNet. For feature extraction, the first stage of transfer learning employs the curated breast imaging subset of the digital database for screening mammography (CBIS‐DDSM) dataset for pretraining, whereas the second stage fine‐tunes the model on the INbreast dataset, resulting in substantial performance improvements. Specifically, the model achieves true positive (TP) rates of 0.99 ± 0.02 and 1.67 false positives (FPs) per image, indicating a substantial enhancement over the STRL methods. This approach provides an innovative framework and serves as a successful example for breast cancer detection.

#### 3.1.6 ResNet50 model

ResNet50 is a 50‐layer deep ResNet that alleviates the vanishing gradient problem in deep networks by introducing residual connections, enhancing training efficiency and accuracy. In 2022, Chen et al. [136] employed the ResNet50 architecture in DBT to diagnose breast cancer in patients with structural distortions, employing gradient‐based class activation mapping (Grad‐CAM) to visualize suspicious areas in 298 patients. In that study, feature extraction was performed via radiomic and DL methods, radiological features were extracted by manually outlining the ROI, key features were identified via support vector machines (SVMs), and finally, the diagnostic performance of the different models was assessed via the DeLong test. The AUC of the imaging radiomics model, which incorporated CC + MLO features, was 0.82, with a sensitivity of 0.78, a specificity of 0.68, and an accuracy of 0.74, whereas the AUC of the DL model was 0.61. Despite its lower accuracy, the trained model allows Grad‐CAM to highlight suspicious areas of structural distortion, facilitating automatic ROI depiction. The results of the study suggest that the developed CAD tool can initially detect subtle pathologic textures on DBT images and subsequently perform further characterization to make a diagnosis.

#### 3.1.7 Visual Geometry Group‐16 Model

Visual geometry group‐16 (VGG16) is a 16‐layer CNN that enhances feature extraction and classification performance via small convolutional kernels and a deep architecture. In 2024, Esposito et al. [137] developed a preprocessing tool called the digital breast imaging tool (DBIT) and evaluated its performance in improving DL‐based CAD systems for cancer detection. They extracted over 200 DBT volumes from a public repository, dividing them into negative and positive (benign and malignant) tumors to form the “original dataset” (raw images) and the “processed dataset” (images processed with DBIT). In the study, 214 features were extracted via PyRadiomics, a SVM was used for feature selection and model construction, and the model performance was evaluated via fivefold cross‐validation and the DeLong test. The classification performance of VGG16 in the original and processed datasets was evaluated in terms of the AUC, accuracy, F1 score, precision, sensitivity, and specificity. The average classification accuracy of VGG16 increased by approximately 12% with the processed dataset. The results demonstrate that DBIT effectively preprocesses images to serve as suitable inputs for DL‐based CAD systems, thereby assisting imaging physicians in the diagnosis of breast cancer.

#### 3.1.8 Visual Geometry Group‐19 Model

Visual geometry group‐19 (VGG19) is a 19‐layer CNN that extends VGG16 with additional convolutional layers, offering enhanced feature representation for more complex image classification tasks. In 2018, Mendel et al. [138] used an ImageNet pretrained VGG19 model to extract features from DBT images for the classification of malignant and benign lesions. Features were selected from the VGG19 model after each maxpool layer, and mean pooling was applied to reduce feature dimensionality. To avoid feature redundancy, a “leave‐one‐out” stepwise feature selection method was used to identify the most frequently selected features, followed by an estimation of the likelihood of malignancy. The study utilized 78 lesion images, including 30 images of malignant lesions and 48 images of benign lesions. The results revealed a significant improvement in classification accuracy when combined with the use of anterior and posterior images from DBT, with DBT images having an AUC as high as 0.98 in the classification of mass and architectural distortion (ARD) lesions, compared with 0.88 in FFDM (*p* = 0.024), highlighting the advantages of DBT in the detection of breast malignancies.

In 2023, Mukhlif et al. [139] proposed a novel method called dual transfer learning, which is based on pattern convergence between the source and target domains. Four pretrained models (VGG16, Xception, ResNet50, and MobileNetV2) were fine‐tuned on sufficient unclassified breast cancer images by adjusting the corresponding final layers. A small number of already classified images of the same disease and target task were also used, and data augmentation techniques were employed to balance the categories and increase the sample size. The experimental results indicate that the proposed method enhances the performance of all the models, whereas data augmentation further improves the performance of the VGG16, Xception, ResNet50, and MobileNetV2 models by 19.66, 34.76, 31.76, and 33.03%, respectively. Notably, the Xception model achieved an accuracy of 99%, a precision of 99.003%, a recall of 98.995%, an F1 score of 99%, a sensitivity of 98.55%, and a specificity of 99.14%.

#### 3.1.9 InceptionV3 Model

InceptionV3 uses a modular Inception architecture and factorized convolutions to improve computational efficiency and feature extraction, making it suitable for various image recognition tasks. In 2023, Harron et al. [140] conducted a comparative investigation of various pretrained CNN models for feature extraction in fuzzy detection with DBT images. The CNN models assessed included ResNet18, ResNet50, AlexNet, VGG16, and InceptionV3. These pretrained CNN models, combined with a SVM classifier, yield promising results in the fuzzy classification of DBT images. Specifically, deep feature extraction methods were used for feature extraction, with InceptionV3 having the highest accuracy of 97% and a maximum AUC of 0.9961.

### 3.2 Breast Lesion Segmentation and Detection with DL on DBT Data

In the task of lesion detection and classification, the development of DL has significantly changed the traditional methods of image segmentation and detection [141, 142, 143, 144]. In 2012, AlexNet marked the rise of DL in computer vision [145, 146], followed by the region‐based CNN (R‐CNN) family [147, 148] (including Fast R‐CNN and Faster R‐CNN) and the You Only Look Once (YOLO) model from 2016 onward, which drove further developments in object detection [149, 150, 151, 152]. For semantic segmentation, models such as the fully convolutional network and U‐shaped network (U‐Net) have achieved breakthroughs in pixel‐level prediction [153, 154], and the Mask R‐CNN and DeepLab series have resulted in further improvements in instance and semantic segmentation [155, 156, 157, 158, 159]. In 2021, transformer architectures, such as the detection transformer and swin transformer, were introduced to perform visual tasks [160, 161]. In 2023, Xia and Wang [162] systematically reviewed transformer‐based approaches, noting that models such as vision transformer and swin transformer have been widely used for image segmentation tasks for brain tumors, stroke, and other diseases. The article summarizes a variety of architectures that fuse convolution with transformer, including SwinUNet, TransBTS, and so on. These methods utilize the global modeling capability of transformer, which significantly improves the segmentation effect in multimodal medical images, and especially show strong adaptive ability under small sample conditions [162]. These advancements have led to significant progress in image segmentation and detection techniques [163, 164].

For segmentation and detection tasks, DBT data present unique advantages and disadvantages compared with other medical data [165, 166, 167]. The higher resolution of DBT allows the capturing of fine structures and lesions in breast tissue, providing clearer images than traditional 2D mammography methods and enhancing the accuracy of detection and segmentation algorithms [168, 169, 170]. However, the high resolution also results in substantial storage requirements and increased computational resources for processing and analysis [171]. The volume of 3D imaging data grows exponentially with respect to that of 2D imaging data, placing greater demands on hardware [172]. Additionally, DBT images usually contain hundreds of slices, making manual lesion labeling extremely time consuming and error prone [173, 174, 175]. Consequently, the segmentation and detection tasks for DBT data are highly complex.

#### 3.2.1 U‐Net Model

U‐Net achieves multiscale feature fusion through an encoder–decoder structure with symmetric skip connections. In 2020, Lai et al. [176] proposed an algorithm for automatically segmenting DBT‐detected images masses using the U‐Net architecture. This approach can be divided into six stages: DBT image preprocessing, patch extraction, data augmentation, voting scheme fusion, segmentation via the U‐Net architecture, and postprocessing. The authors employed a 23‐layer U‐Net model for segmentation, after which they compared the performance of their model with that of linear discriminant analysis, SVM and CNN. Their model outperformed the other methods, with accuracies, sensitivities, specificities, and AUCs for the entire experimental dataset of 0.871, 0.869, 0.882, and 0.859, respectively. The results demonstrate that the proposed U‐Net‐based system is an effective solution to the problem of DBT mass segmentation.

#### 3.2.2 Faster R‐CNN

Faster R‐CNN is an efficient object detection framework that integrates an RPN for generating region proposals and a CNN for object classification and localization. In 2019, Fan et al. [177] designed a CAD system for DBT masses via Faster R‐CNN. They first collected a dataset comprising 89 patients and 105 masses. The detection architecture employs a CNN with a region proposal network to generate region proposals (e.g., bounding boxes) with mass likelihood scores for each slice. The masses detected on consecutive 2D slices are then merged into a 3D DBT volume through a slice fusion procedure. The performance of the CAD system was evaluated with free‐response ROC curve analysis, and the results indicated that, in a comparison between R‐CNN‐based CAD systems and DCNN‐based CAD systems, the AUCs were 0.96 and 0.92, respectively [178]. The results demonstrated the utility of faster R‐CNN for pretesting ROIs and distinguishing true masses from FPs in DBT. They further compared the performance of this method with that of a previous DCNN‐based CAD system, showing that the faster R‐CNN could improve prescreening and reduce FPs in mass CAD systems.

#### 3.2.3 Mask R‐CNN

Mask R‐CNN is an extension of Faster R‐CNN that adds a parallel branch, such as segmentation, to achieve object classification, localization, and pixel‐level segmentation. Additionally, in 2022, Fan et al. [178] proposed the Mask R‐CNN CAD system framework for breast lump detection and segmentation of DBT images, which uses ResNet‐50 as a feature extractor. The study was based on a 364‐sample design divided into a training set (*n* = 201) and a test set (*n* = 163). In lesion detection, the 3D‐Mask R‐CNN achieves a sensitivity of 90% and an FP rate of 0.8 per lesion, which outperforms the performance of the 2D‐Mask R‐CNN and Faster R‐CNN. Statistical analysis revealed that the 3D‐mask R‐CNN significantly outperformed 2D‐based detection in patients with different features (*p* < 0.05).

#### 3.2.4 YOLO Model

YOLO is an end‐to‐end object detection framework that reformulates object detection as a single neural network regression problem, enabling real‐time object detection and localization. In 2022, Hossain et al. [179] presented a novel algorithm for detecting breast lesions on DBT images using multidepth level convolutional models. They employed YOLOv5 as the base network and enhanced the detection algorithm through data augmentation and fine‐tuning, ultimately developing an integrated algorithm for medium‐to‐large models. The integrated model achieves an average sensitivity of 0.786 FPs per DBT volume on the DBTex independent test set, with a 2 FP per image sensitivity of 0.743. The results indicate that the FP outcomes of nonbiopsied benign lesions provide valuable information for the lesion detection algorithm and that the integrated detection model improves lesion detection.

### 3.3 Breast Medical Image Generation with DL on DBT Data

Since 2012, DL has achieved tremendous progress in the field of image generation. In 2013, autoencoder and variational autoencoder (VAE) improved the quality of image generation through unsupervised learning [180, 181, 182]. In 2014, the generative adversarial network (GAN) was proposed by Ian Goodfellow et al. [183]; this system significantly enhanced the quality of generated images through adversarial training between a generator and discriminator. Subsequent variants, such as deep convolutional GAN, Pix2pix, and cycle GAN, further advanced image transformation and style migration [184, 185, 186, 187]. After 2017, the Wasserstein GAN, big GAN, and style GAN families demonstrated notable improvements in training stability and image quality [188, 189, 190, 191]. Since 2020, generative transformation models such as vector quantized VAE 2, diffusion models for autoregressive language learning‐E, and contrastive language‐image pretraining, which combine VAE and transformer architectures, have substantially improved image quality and consistency in multimodal generation tasks [192, 193, 194, 195, 196, 197].

The applications of generative models in the field of medical images primarily include image enhancement and restoration [198, 199], image segmentation [200, 201], image synthesis [202, 203], image transformation [204], and anomaly detection [205]. A prominent challenge with DBT data in the context of DL is the high cost of manual delineation and annotation. DBT images typically consist of hundreds of slices, and manually annotating lesions in each slice requires medical experts to spend substantial time and effort, resulting in fewer publicly available DBT datasets with annotations such as outlining [36]. Therefore, image synthesis, dataset extension, and improvements in the generalization ability of the model through data augmentation techniques and GANs have become important areas of research.

#### 3.3.1 Generative Adversarial Network

GAN achieves high‐quality data generation through adversarial training between a generator and a discriminator. Although primarily used for image generation, GANs have also played significant roles in improving breast cancer detection through various innovative approaches [206, 207, 208]. GANs can augment DBT datasets by generating synthetic images that closely resemble real cases, even when trained on a small dataset [209, 210]. These synthetic images can be integrated into the original dataset, enriching and diversifying the available training data and improving the robustness and generalizability of machine learning models dedicated to breast cancer detection, greatly facilitating the development of more accurate and reliable detection algorithms [211, 212].

In 2021, Lee and Nishikawa [213] developed a conditional GAN (CGAN) model to simulate normal‐appearing mammograms based on mammograms of the opposite breast and processed the images with a CNN. After testing, the fusion AUC of the CNN reached 0.77, which was significantly better than that of the CNN model when only real mammograms were used (AUC of 0.70) and that of the CNN model when only simulated mammograms were used (AUC of 0.68). These results indicate that the CGAN model could aid in the detection of breast cancer [213]. In addition, the study trained a deep image‐to‐image network for feature segmentation on DBT images and subsequently constructed a task‐driven GAN model for simultaneous synthesis and parsing of unseen real DBT images. In 2022, Staffa et al. [214] proposed an evolutionary GAN (E‐GAN) to augment and balance DBT image datasets. The quality of the synthetic images generated by the E‐GAN was significantly improved at a later stage of the learning process, especially in terms of detail and fidelity, with the structure and texture of the breast becoming more visible [214].

Breast disease classification and diagnosis, lesion segmentation and detection, and medical image generation can be achieved on DBT data via DL models. The integration of image data obtained from DBT is essential for developing an intelligent early breast cancer screening system based on DL. A comprehensive and in‐depth exploration of the application of DL in the field of DBT is particularly necessary to fully realize its potential.

### 3.4 Application of DL to Other Areas of DBT

DL has various potential applications in the field of DBT [58]. It has already been widely used in breast disease diagnosis and classification, lesion segmentation and detection, and medical image generation [215]. Additionally, in combination with DBT, DL has shown great promise in predicting breast cancer molecular subtypes, chemotherapy response, and recurrence risk [216, 217, 218]. DL can also be applied in various clinical environments, suggesting unprecedented opportunities for its application in DBT. Table 1 illustrates several potential applications of DL in DBT in addition to those described previously.

**TABLE 1:** Summary of other applications of DL in DBT.

Paper/reference	Core algorithm	Model application	Evaluation metric	Best results
Shimokawa et al. [219]	DL	Predict the presence of stromal invasion	AUC	0.750
Schmitgen et al. [220]	Random forest	Individualized treatment prediction	Sensitivity	0.770
Rigaud et al. [221]	DL	Automated assessment of breast density	Binary classifications	0.750
Michielsen et al. [222]	CNN	Improve accuracy and precision of iodine quantification in contrast‐enhanced tomosynthesis	Dice similarity coefficient	0.975
Jang et al. [223]	CNN	Signal known statistically and background known statistically detection tasks in breast tomosynthesis images	Optimal detection performance	Signal known exactly: 0.912 Signal known statistically: 0.824
Gao et al. [224]	DCNN	Improve the image quality of DBT in terms of image noise and MC conspicuity	AUC	0.970
Su et al. [225]	DL	Improve the DBT imaging performance	Azimuthal symmetry function curves/the image reconstruction time	5.900 mm/9.300 s
Lee et al. [56]	Deep neural network	Improved breast cancer classification performance	AUC	0.910
Yang et al. [226]	Convolutional inception style transfer module	Classify lesion malignancy in DBT	AUC	0.802
Wang et al. [49]	Multiscale feature deep neural network	Breast mass classification using DBT	AUC	0.870
Wang et al. [227]	CNN	Alleviate the impacts for the accurate and rapid detection of microcalcification clusters in DBT	Sensitivity	93.51%
Samala et al. [228]	DCNN	Masses in DBT volume	AUC	0.900
Mota et al. [229]	CNN	An automatic classification of a complete DBT image for the presence or absence of MCs	AUC	94.19%
Lai et al. [176]	U‐Net	Improve the automatic segmentation accuracy of breast masses in DBT images	Specificity/AUC	0.882/0.859
Conant et al. [230]	DCNN	Shorten DBT reading time while maintaining or improving accuracy	AUC	0.852
Xiao et al. [231]	CNN	Improve the classification performance of benign and malignant MCs in DBT	AUC	0.8837
Teuwen et al. [104]	CNN	Predictions of breast density	Structural similarity index	0.910
Whitney et al. [232]	CNN	Distinguish between benign or malignant lesions	AUC	0.930
Matthews et al. [233]	DL	Predict breast density	AUC	0.980
Kim et al. [234]	CNN	Characterize malignant masses in DBT	AUC	0.910

*Note*: Aside from the source code of Ref. 133 and the database of Ref. 143, none of the source codes or databases of the papers listed in Table 1 are publicly available.

Abbreviations: AUC, area under the curve; CNN, convolutional neural network; DBT, digital breast tomosynthesis; DCNN, deep convolutional neural network; DL, deep learning; MC, microcalcification cluster; U‐Net, U‐shaped network.

*Source code*: https://www.mdpi.com/article/10.3390/cancers14205003/s1.

*Database*: https://wiki.cancerimagingarchive.net/pages/viewpage.action?pageId=39879200.

DBT has the potential to allow the extraction of quantitative biomarkers of breast cancer [235]. In 2019, Tagliafico et al. [236] extracted a set of 106 quantitative features from DBT images, including morphological features, grayscale/scaling statistics, and texture features. They then applied the least absolute shrinkage and selection operator method to select the most predictive features, yielding 34 features that were significantly correlated with Ki‐67, with correlation coefficients exceeding 0.5 for five of them. The quantitative radiographic features of the breast tumors extracted from the DBT images were associated with Ki‐67 expression in breast cancer [236].

DL also provides a way for DBT to predict molecular subtypes of breast cancer. This involves the detailed exploration of specific molecular subtype features visualized on DBT images, which can help classify lesions based on receptor status and molecular subtype [237, 238, 239]. In 2019, Cai et al. [240] retrospectively analyzed 234 breast cancer patients with surgical, complete pathological, and immunohistochemical data, classified the patients’ tumors into four molecular subtypes and then assessed the associations between the clinical features of each molecular subtype and DBT features. The results revealed that the calcification score and lymph node size significantly differed among the four molecular subtypes. Subgroup analyses based on tumor size, calcification score, and lymph node size revealed a significant difference in the distribution of patients with a lymph node size ≥1.5 cm versus those with a lymph node size <1.5 cm. In summary, diagnostic imaging features such as the calcification score and lymph node size determined by DBT can be used as auxiliary diagnostic indicators for the molecular subtype of breast cancer [240].

DL has shown promise for risk prediction and prognosis in DBT [241]. In 2020, Conant et al. [242] compared the resection rate, complete dissection rate, and biopsy recommendation rates; positive predictive value of recall; positive predictive value of biopsy; false‐negative rates; and biology, size, and lymph node status of screen‐detected and interval cancers in patients consecutively screened with DBT versus DM. The results suggested that DBT screening detects a greater proportion of cancers with a poor prognosis than does DM screening. The combination of DL with DBT, together with other clinical indicators, aims to facilitate early prediction of patient and survival outcomes [243]. The potential application of combining DBT with DL could also be extended to predicting cancer recurrence, as demonstrated through some recent developments in breast imaging [244]. This includes combining AI with imaging genomics, using the features of the latter to predict the risk of recurrence in patients with breast cancer.

DL has demonstrated a wide range of applications in the field of DBT, significantly improving the accuracy of breast disease diagnosis and lesion segmentation and detection and offering the potential for fully automated detection. When combined with DL, DBT technology excels in predicting the molecular subtypes of breast cancer, assessing the response to chemotherapy, and predicting the risk of recurrence, thereby greatly improving the sensitivity in diagnosing breast cancer.

## 4 Machine Learning in the Field of DBT

Using the enhanced features extracted from DBT images, different research teams have significantly improved the effectiveness and diagnostic accuracy of early breast cancer screening by combining various techniques, including logistic regression models and machine learning methods [245, 246, 247]. In 2022, Eriksson et al. [248] developed a logistic regression model based on DBT information, producing a fundamental machine learning‐based classification algorithm that has been widely studied in the field of early breast cancer screening. This model uses enhanced image features extracted from DBT images to predict the risk of breast cancer in women who undergo annual screening [248].

Similarly, Johnson et al. [249] reported a decrease in the incidence of interstitial cancer following DBT screening. Their findings, derived from a prospective, population‐based DBT screening trial, indicated a rate of 1.6 interval cancers per 1000 women screened, a reduction from the accepted value that suggests the potential benefits of their method, such as enhanced early detection, leading to reduced breast cancer mortality rates [249]. Additionally, Sharpe et al. [76] analyzed the effect of DBT on recall and cancer detection rates in breast cancer screening. By combining single‐ and mixed‐effects logistic regression models, their results demonstrated that the implementation of DBT significantly improved cancer detection rates, markedly increasing the efficacy of breast cancer screening [76].

In the medical field, machine learning techniques have been widely used for feature extraction from DBT images, as well as for screening and diagnosing breast cancer, as shown in Table 2. In 2022, Niu et al. [250] developed a nomogram that combines radiomic features with clinically important factors. Compared with the classical mammography‐DBT assessment method, their nomogram demonstrated superior diagnostic ability and was recommended as a valuable tool for assisting clinicians in the early screening of breast cancer [250].

**TABLE 2:** Non‐DL AI models in DBT.

Paper/reference	Core algorithm	Model type	Evaluation metric	Best results
Conant et al. [64]	Logistic regression	DBT‐based logistic regression model	Sensitivity specificity	0.909 0.913
Niu et al. [250]	Multivariate logistic regression	DBT‐based radiomics model	AUC	0.980
Wang et al. [251]	Logistic regression	DBT‐based combined radiomics nomogram	AUC	0.905
Eriksson et al. [248]	Elastic net logistic regression, nested cross‐validation	DBT‐based risk model	AUC	0.820
Bahl et al. [252]	Multivariable logistic regression	DBT‐based DL models	Adjusted odds ratio	0.880–1.140
Kim et al. [253]	Multivariable logistic regression	DBT‐based logistic regression model	TP, FP	18.7 vs. 21.7% 75.9 vs. 77.6%
Peng et al. [254]	Random forest	DBT‐based random forest classifier	AUC	0.834
Sakai et al. [255]	Support vector machine	DBT‐based support vector machine classifier	Correct identification rate	0.840
Wels et al. [256]	Multiple multivariate random forest regression	DBT‐based machine learning methods	Localization error	22.480 ± 8.670 mm
Samala et al. [132]	Feature selection, random forest classification	DBT‐based deep DCNN	AUC	0.900

*Note*: None of the source codes or databases of the papers listed in Table 2 are publicly available.

Abbreviations: AUC, area under the curve; DBT, digital breast tomosynthesis; DCNN, deep convolutional neural network; DL, deep learning; FP, false positive; SVM, support vector machine; TP, true positive.

In 2020, Sakai et al. [255] explored the use of various classifiers, including SVM, naive Bayes, random forest, and MLP classifiers, to classify a variety of extracted radiomic features, with a particular focus on comparing their accuracy. Among these classifiers, the SVM‐based classifier was the most effective, achieving accuracies of 55 and 84% in detecting benign and malignant tumors, respectively. These results indicate that the proposed method can assist imaging physicians in diagnosing lesions more accurately [255].

Radiomics employs machine learning technology to analyze and interpret medical images, as demonstrated by numerous studies applying such methods to this field [257, 258, 259, 260]. Radiomic methods can extract specific imaging features (e.g., first‐order features, textural elements, intensities, gradients, and curvatures) from DBT images to identify ROIs [251, 255, 261]. This capability plays a key role in all aspects of breast cancer management, including diagnosis, subtype differentiation, treatment response assessment, and prognosis.

For example, Tagliafico et al. [262] used radiomics in DBT to assess dense breasts, extracting 104 different features from the images of 20 patients and analyzing the differences in three specific features between healthy individuals and cancer patients, achieving an AUC of 0.567. Moreover, Fusco et al. [263] utilized radiomics to differentiate between malignant and benign lesions, focusing on the morphological features distinguishable on DBT, and achieved an AUC of 0.74 following univariate analysis. Together, these studies illustrate the ability of radiomics to identify ROIs by extracting specific imaging features from DBT images. Advances in machine learning technology are critical for improving the early screening and diagnosis of breast cancer.

## 5 Public Mammography Databases

DL models in the field of DBT require a substantial amount of data and comprehensive databases [264]. Currently, DM research benefits from numerous datasets sourced from multiple medical centers, but the limited availability of public datasets hampers the development of the DBT field [265, 266, 267].

In this subsection, we present several key databases that allow researchers access to DBT‐related data. These databases can be categorized into two types: DM datasets and DBT datasets. Notably, the DM and FFDM datasets are relatively abundant in publicly available sources. We list 11 widely used public datasets with data sizes ranging from a few hundred to hundreds of thousands of samples. Most of these datasets contain detailed regional‐level annotations.

We summarize the currently available mammography databases, including DM and FFDM databased. The MIAS and INbreast databases are specifically dedicated to mammography image analysis. The MIAS database was constructed from Joyce‐Loebl scanning of films from the UK National Breast Screening Program [268]. It includes images of women of various ages and ethnicities, with annotations detailing lesion location, size, and morphology. INbreast comprises data from 115 women (410 images), including patients with both breasts and those who had undergone mastectomy and lesion types including masses, calcifications, asymmetries, and deformities [269]. The DDSM [270], LAPIMO [271], BCDR‐DM [272], CSAW‐CC [273], OMI‐DB [274], and VinDr‐Mammo [273] databases provide extensive collections of mammography and FFDM images. These databases offer not only conventional X‐ray images but also ultrasound images and a rich array of images with BI‐RADS classification annotations to support the development, training, and performance testing of mammography CAD programs.

**FIGURE 4:**
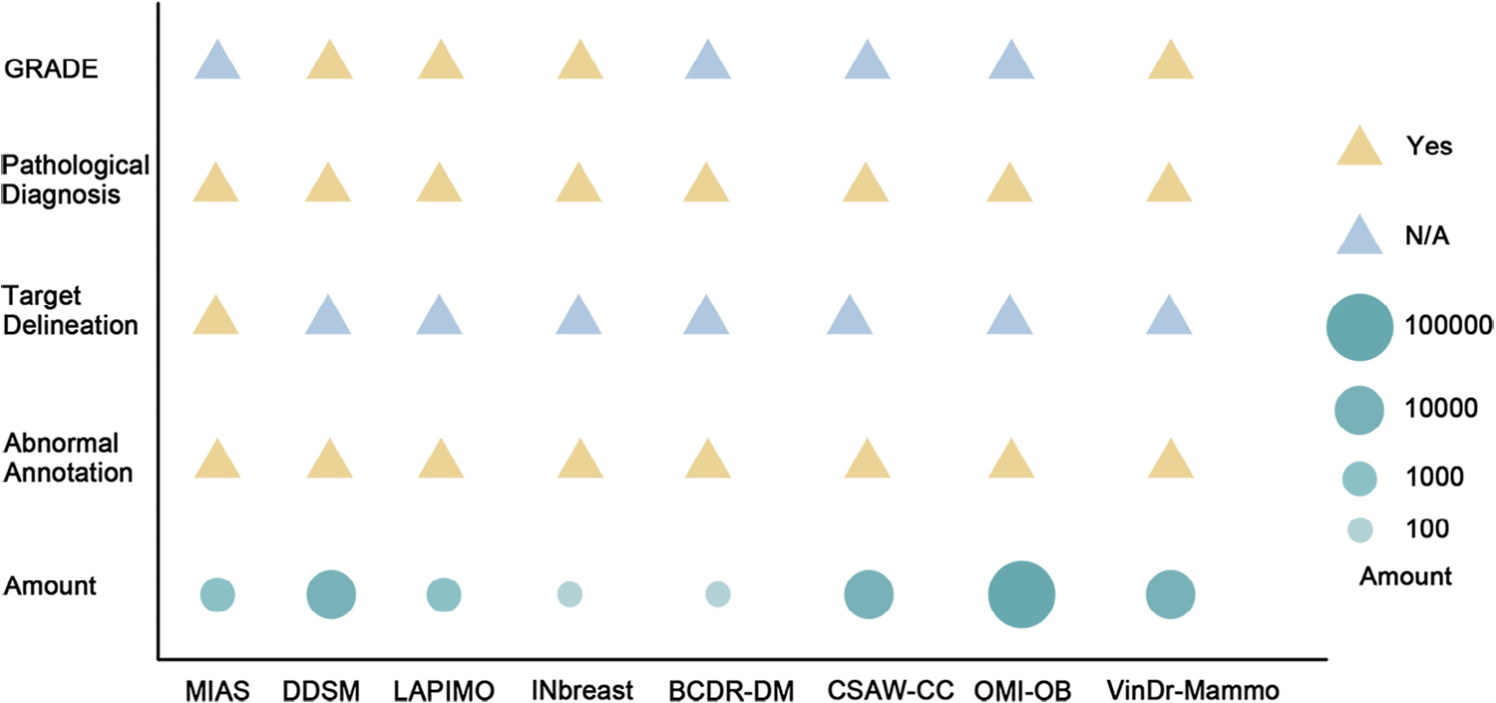
Detailed comparison of breast cancer image databases. We make detailed comparisons between databases based on several key attributes, including GRADE, that is, whether information on the severity grading of breast cancer is included; pathological diagnosis, that is, whether benign and malignant information is included; target delineation, that is, whether the delineation of target regions for breast cancer is included; abnormal annotation, that is, whether abnormal annotations are included; and amount, that is, the number of images in the dataset. The yellow triangles indicate that the attribute is present, whereas the blue triangles represent “not applicable,” that is, the data are not provided. The size of the circle indicates the order of magnitude of the images in the dataset. For example, the largest circle represents an order of magnitude of more than 100,000 images, whereas the smallest circle represents an order of magnitude of fewer than 100 images. The representative breast cancer image databases include MIAS, DDSM, LAPIMO, INbreast, BCDR‐DM, CSAW‐CC, OMI‐OB, and VinDr‐Mammo.

The DDSM, LAPIMO, INbreast, and VinDr‐Mammo databases contain grading of recommendations, assessment, development, and evaluation annotations. The OMI‐DB and VinDr‐Mammo databases, containing 148,461 and 5000 four‐view FFDM images, respectively, are annotated with detailed assessments and lesion findings at the breast level, providing high‐quality resources for research and clinical diagnosis (Figure 4).

In contrast, few publicly available DBT datasets exist because of the relative recency of DBT technology. The main shortcomings of DBT databases with respect to other databases (e.g., DM databases) are their small number of datasets, insufficient detailed annotations, and lack of patient diversity. This limits the options available to researchers and clinicians for training and evaluating DL models. Currently, DBT databases are widely used in training tumor detection and classification algorithms, evaluating their effectiveness in the diagnosis of various conditions, improving image reconstruction and enhancement techniques, conducting multimodal image research, training and educating clinicians, and developing optimized CAD systems [275, 276, 277]. These applications not only enhance the early detection and diagnosis of diseases such as breast cancer but also promote the advancement of related technologies and algorithms.

However, most datasets constructed in these studies are not publicly available. The BCS‐DBT dataset, consisting of 5060 samples, is currently the only publicly accessible DBT dataset [167, 278]. Two imaging physicians annotated these samples, documented the presence of masses and ARDs, and assessed whether the findings were benign or malignant lesions [279, 280]. However, the details of their regional‐level annotations are very limited. This discrepancy highlights the scarcity of comprehensive DBT datasets, especially considering the volume and depth of information needed. Therefore, the development and availability of large‐scale, well‐annotated public DBT databases is highly important. A comparison between the DBT and DM databases is shown in Table 3.

**TABLE 3:** DBT and DM databases.

Dataset	Modality	Samples	Short description	Uniform resource locator
Yonsei University	DBT	173	Malignant breast lesions were included in 169 patients.	Unavailable
Duke University	DBT	4348	The data consist of 19,230 reconstructed volumes from 4348 patients. Cancerous lumps and building distortions are marked by the radiologist with bounding boxes.	Unavailable
The TOMMY trial	DBT	7060	Provides women (47–73 years of age) recalls for further evaluation after routine breast screening and women (40–49 years of age) who participate in annual mammograms.	Unavailable
BCS‐DBT	DBT	5060	DBT dataset consists of a collection of patients who has a DBT exam at Duke Health system within January 1, 2014 to January 30, 2018.	https://sites.duke.edu/mazurowski/resources/digital‐breast‐tomosynthesis‐database/
MIAS	DM	322	Provides an organization of UK research groups interested in the understanding of mammograms and generates a database of digital mammograms.	http://peipa.essex.ac.uk/pix/mias/all‐mias.tar.gz
DDSM	DM	10,480	Provides a database established by medical institutions in the United States to store breast cancer images, including: cancer, normal, benign, benign without callback.	http://marathon.csee.usf.edu/Mammography/Database.html
LAPIMO	DM	320	Development of a database with significant number of cases and digitized mammographic images forward to the development, training, and performance tests of mammography CAD schemes.	http://lapimo.sel.eesc.usp.br/bancoweb/english/index.php
INbreast	DM	115	Provides a total of 115 patients (410 images), of which 90 are from women with both breasts (four images each) and 25 from mastectomy patients (two images each). Including several types of lesions (lumps, calcifications, asymmetry, and deformation).	http://medicalresearch.inescporto.pt/breastresearch/index.php/Get_INbreast_Database
BCDR‐DM	DM	116	Provides 724 (723 female and one male) Portuguese patients cases (with ages between 27 and 92 years old), including 1042 studies, 3612 MLO and/or CC mammography incidences, and 452 lesions clinically described (already identified in MLO and CC views).	https://bcdr.eu/information/about
CMMD	DM	1775	Provides 3728 mammograms performed by 1775 patients between July 2012 and January 2016 are presented, and the biopsy confirmed to be of benign or malignant tumor type. For 749 of these patients (1498 mammography), we also include the molecular subtypes of the patients.	https://www.cancerimagingarchive.net/collection/cmmd/
CSAW‐CC	DM	65,240	CSAW includes 499,807 women invited to be screened between 2008 and 2015, with a total of 1,182,733 completing screening tests. Approximately 2 million mammogram images have been collected, including images of all women with breast cancer.	https://link.springer.com/article/10.1007/s10278‐019‐00278‐0
OMI‐DB	FFDM	172,282	Consists of several relational databases and cloud storage systems, containing mammography images and associated clinical and pathological information. The database contains over 2.5 million images from 173,319 women collected from three UK breast screening centers.	https://medphys.royalsurrey.nhs.uk/omidb/
DMID	DM	510	Detailed radiological reports and pathology information via pixel‐level annotations and associated segmentation masks to support mammography for DL systems.	https://figshare.com/articles/dataset/_b_Digital_mammography_Dataset_for_Breast_Cancer_Diagnosis_Research_DMID_b_DMID_rar/24522883
VinDr‐Mammo	FFDM	5000	A Vietnamese digital mammography dataset with mammal‐level assessment and extensive lesion‐level annotations, each examination has four standard views and is a double reading, and provides categories, locations, and BI‐RADS assessments of nonbenign findings.	https://vindr.ai/datasets/mammo

Abbreviations: BI‐RADS, breast imaging reporting and data system; CAD, computer‐aided diagnostic; DBT, digital breast tomosynthesis; DL, deep learning; DM, digital mammography; FFDM, full‐field digital mammography.

## 6 Challenges and Prospects of DL Applications in DBT Imaging

**FIGURE 5:**
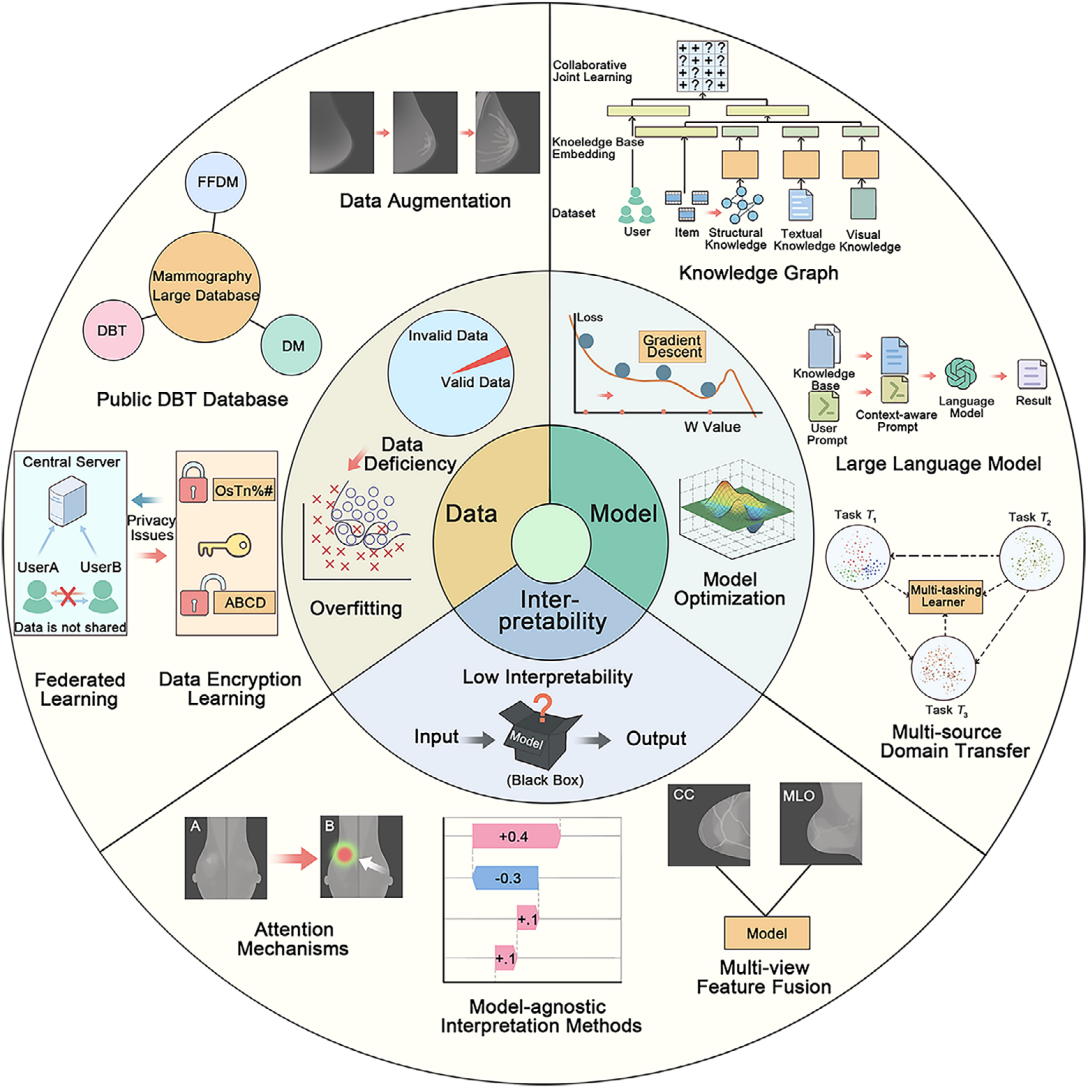
Challenges and future prospects of DL techniques for DBT applications. The challenges are grouped into three categories: data challenges, model challenges, and interpretability challenges. The training of DBT‐DL models depends on the availability of a substantial, high‐quality annotated training set, as there is a paucity of publicly available datasets for DBT. A lack of data can lead to model overfitting. In addition to building public databases, the use of data augmentation, federated learning, encrypted data learning, and other methods is expected to improve model generalizability across different datasets. In addition, the training process of DL models presents numerous challenges. Multisource domain migration, LLM, and knowledge graphs are expected to facilitate the implementation and dissemination of DBT techniques in clinical settings. Since DL models are opaque, resulting in low interpretability. Attention mechanism networks, multiperspective feature fusion, and in‐model interpretation approaches are expected to improve interpretability.

Although DL techniques have shown great potential in analyzing breast cancer images, their application in the DBT field remains limited. Next, we review the three main challenges faced in the application of DL techniques in DBT research and propose directions for future development. Figure 5 provides an overview of the deployment of DL and machine learning in the DBT domain, highlighting their advantages and challenges.

### 6.1 Challenges and Perspectives Related to DBT Datasets

The training of DL models relies on large, high‐quality annotated training sets [281, 282], and public datasets provide a foundation for researchers to develop and evaluate models. However, the lack of public DBT datasets remains a significant challenge, with existing well‐known mammography datasets (e.g., DDSM and OMI‐DB) not applicable to DBT and the sharing of image data severely restricted owing to privacy policies [283, 284, 285]. Therefore, further development of DL‐based DBT models requires the collection of a large amount of accessible data. Typically, DL models perform best on large amounts of highly annotated data, but DBT typically produces more than 100 times more images than DM does; however, malignant features are usually only visible in a few slices [286, 287]. Previous studies have improved model performance by collating and annotating DBT image datasets, applying multi‐instance learning, and so on [288, 289]. Buda et al. [167] collated data from more than 22,000 3D‐DBT volumes from 5060 patients to facilitate the development of a breast cancer screening model. Lotter et al. [287] also developed a new method to efficiently train a model using DBT data labeled with breast‐level labels while maintaining the interpretation of lesion location. while maintaining interpretability of the lesion location, thereby significantly reducing the need for detailed annotated data without sacrificing performance.

The lack of a common DBT dataset limits the generalization of DL models because their repeatability relies on consistent results under similar conditions, a problem exacerbated by differences in the range of angles, acquisition methods, and reconstruction techniques from different vendors [61, 290, 291]. In addition, DBT images vary significantly from the appearance of normal breast tissue, so image standardization is critical in DBT applications [292, 293]. Researchers use different datasets and evaluation strategies, making results difficult to evaluate and compare. The test sets of many studies tend to come from a single institution and exclude specific data, which obscures the true performance of the algorithm [60]. To this end, data augmentation techniques can be used to improve the learning and generalization capabilities of deep networks [294, 295, 296, 297]. For example, Garrucho et al. [298] used synthetic high‐density FFDM as data enhancement in the training of a breast mass detection model, and the results showed that this method improved the sensitivity and accuracy of the model on small datasets and enhanced the domain generalization ability of the training model on large databases.

Another important reason for the lack of DBT public datasets is patient privacy protection and ethical issues [299, 300]. Due to data privacy and regulatory issues, data are usually retained within the hospital host server and cannot be easily shared [301, 302]. Ethical requirements to ensure the security and confidentiality of patient data. Failure to properly protect patient privacy can lead to data breaches and legal liabilities, undermining patient confidence in the healthcare system [303, 304]. This ethical consideration has led healthcare organizations to take a more cautious approach to sharing sensitive data, limiting the construction of and access to DBT public databases. Data privacy protection methods range from data anonymization and obfuscation to federated learning and encrypted data learning. Federated learning is a private, distributed, and decentralized machine learning method that uses private data to train shared models locally without exchanging original patient data [305, 306, 307]. In 2019, Sheller et al. [308] demonstrated federated learning on clinically acquired brain tumor segmentation data for an interagency segmentation task. Their study revealed that federated semantic segmentation models (Dice = 0.852) performed similarly to models trained on shared data (Dice = 0.862) and outperformed two alternative collaborative learning methods [308].

In summary, the challenges faced by datasets during the training of successful models are related to the generalization ability of the model and data privacy protection. In the future, data privacy‐preserving methods such as federated learning and encrypted data learning are expected to improve the generalization ability of models for different datasets while meeting the requirements of data privacy preservation. In addition, we encourage research teams to make DBT datasets publicly available to promote the further application of DL models in the field of DBT.

### 6.2 Challenges and Perspectives Related to Modeling

New developments in DL models in the DBT field continue to emerge, which we categorize into three parts. We also outline the challenges associated with model training and present prospects for the application of multiple‐source domain transfer (MSDT), large language models (LLMs), and knowledge graphs in the DBT domain.

In the field of the diagnosis and classification of breast cancer, DL has made significant progress, with multimodal data fusion achieving encouraging results [309, 310, 311]. The synergistic application of DM, DBT, and contrast‐enhanced magnetic resonance imaging has been demonstrated to enhance the identification of malignant tumors and reduce the incidence of interstitial cancer [312, 313]. A groundbreaking study by Shoshan et al. [86] used an ensemble of 50 DL and machine learning classifiers to combine DBT data with numerous clinical parameters, including patient age, ethnic background, hormonal status, and family history of breast cancer. Their findings highlighted the efficacy of AI in enhancing breast cancer screening programs. Notably, the AI‐driven approach resulted in a significant reduction in the clinical workload by 39.6% and a 25% reduction in patient recall without compromising sensitivity [86]. Thus, this multimodal screening paradigm shows great promise in enhancing DBT by improving diagnostic accuracy and streamlining the screening process.

In the field of early screening and diagnosis, Rodriguez‐Ruiz et al. [314] introduced a DL‐enhanced interactive tool for imaging students that calculates a localized cancer likelihood score after selecting a specific breast region. This innovation significantly improved the AUC, increasing sensitivity while maintaining specificity and accuracy without prolonging the reading time [314]. In addition, the application of AI in the design and implementation of clinical trials is equally significant. Dong et al. [315] aimed to analyze the characteristics of registered AI trials for cancer diagnosis by searching the ClinicalTrials.gov database to statistically and analytically analyze the design and outcomes of 97 clinical trials involved. The results revealed that most of the trials were observational in design and that the number of interventional trials was low, indicating that the field is in dire need of more high‐quality clinical validation [315]. The use of AI in clinical trials is still in its infancy, but it is a rapidly evolving field. As regulators provide more guidance on the acceptability of AI in specific areas, its use will expand, and its implementation will increase rapidly.

Important breakthroughs in lesion segmentation and detection have also been achieved by DL [316, 317, 318]. Advanced segmentation models can accurately identify and segment structures and lesions in the breast, aiding in diagnosis and treatment. U‐Net and its variants, as well as the DeepLab family, are advanced segmentations models that have been applied to DBT [319, 320, 321, 322, 323]. In 2023, Bobowicz et al. [324] proposed a clustering‐based constrained attention multiple instance learning (CLAM) classifier that can be trained efficiently despite a relative scarcity of data. A feature extractor pretrained on ImageNet (ResNet18, ResNet34, ResNet50, and EfficientNetB0) was used, resulting in an AUC of 0.848. The attention map of the CLAM algorithm highlights the features in the image most relevant for the algorithm [324].

The field of medical image generation has also benefited from developments in DL, particularly through the application of GANs and VAEs. In 2019, Cogan et al. [325] presented a comprehensive mammogram screening solution consisting of three main components: a machine learning algorithm to accept or reject images as valid mammograms, an artificial neural network to locate potential malignancies, and a web service for uploading images and viewing results. The image receiver is primarily a class of SVMs constructed based on features derived from a VAE. If an image is accepted as a mammogram, the malignant tumor recognizer (ResNet‐101 Faster R‐CNN) will locate the tumor in the mammogram. In the test data, the malignant tumor recognizer achieved an AUC of 0.951 [325]. In 2023, Balaji [326] proposed a 3D Connected‐UNet based on an encoder–decoder architecture for tumor segmentation from 3D MR images and evaluated it on the INbreast and private datasets. The experimental results show that the proposed model outperforms existing breast tumor segmentation methods [326].

MSDT may be a potential optimization approach for integrating DM, FFDM, and DBT data, mitigating variability between datasets and enhancing the generalization ability and diagnostic accuracy of models in the DBT field [327, 328, 329]. Specifically, MSDT can reduce data bias, resulting in more stable and accurate models when processing images from various sources, thereby improving the sensitivity and specificity of early breast cancer detection [330, 331]. Additionally, MSDT can significantly reduce the need for and cost of labeled data, as models can be effectively trained using multiple unlabeled datasets. This approach not only addresses the challenge of data scarcity but also accelerates the adoption of DBT technology in clinical settings [332, 333].

In the future, with advancements in transfer learning algorithms and computational resources, the application of the MSDT in DBT is expected to further enhance image quality, diagnostic efficiency, and patient outcome prediction. Regularization methods (e.g., dropout) and the development of more robust model architectures (e.g., transformer) have been proposed for improving model adaptability across different sets of data and tasks [334, 335, 336, 337, 338]. In 2019, Aslani et al. [339] proposed the use of secondary networks and corresponding regularized loss terms to learn domain‐specific knowledge. Their study demonstrated that networks with independent branches produced more accurate segmentation, such as a dice similarity coefficient (DSC) of 0.7649, than did single‐branch networks with all modalities stacked, highlighting the importance of the fluid‐attenuated inversion recovery modality for multiple sclerosis lesion segmentation (DSC > 0.65) [339].

These secondary networks learn to predict the class of the input scanner domains, encouraging the backbone segmentation network to ignore domain‐specific information and helping it outperform other baseline networks in generalizing to new data points. In 2022, Sendra‐Balcells et al. [340] evaluated the potential of domain generalization for increasing the number of images with data augmentation, domain blending, transfer learning, and domain adaptation techniques. Their results indicated that combining data augmentation with transfer learning can produce single‐center models that generalize well to new clinical centers not included in the training data [340]. Single‐domain neural networks enriched with appropriate generalization procedures can meet or exceed the performance of multicenter, multivendor models for augmented imaging, thereby eliminating the need for comprehensive multicenter datasets for training generalizable models [340, 341].

Recently, LLMs have achieved important milestones in addressing various challenges in the biomedical field [342, 343, 344, 345]. For example, in 2024, Huang et al. [346] introduced the clustered rule‐interval short palindromic repeats‐generative pretrained transformer (CRISPR‐GPT) that enhances domain knowledge and external tools to automate the process of designing CRISPR‐based gene editing experiments. CRISPR‐GPT leverages the reasoning capabilities of LLMs to facilitate the selection of CRISPR systems and experimental design, demonstrating the potential of LLMs in complex biodiscovery tasks [346]. At the same time, LLMs play an important role in clinical trials. In 2024, Jin et al. [347] introduced TrialGPT, an LLM‐based framework for assisting in the matching of patients to clinical trials. Evaluated across 183 patients and more than 18,000 trial annotations, TrialGPT not only achieved 87.3% predictive accuracy, but also significantly reduced the time for clinical trial matching, demonstrating its great potential to enhance patient recruitment efficiency [347].

DL models are highly dependent on the amount of data, and the number of mammograms required for a human physician to achieve better diagnostic performance is much less than that required for DL models. Human doctors utilize their extensive experience and comprehensive background knowledge in the diagnostic process and can make accurate diagnoses with fewer images, whereas DL models require large amounts of data for training to ensure their accuracy and generalizability in various situations. However, LLMs have the potential to mitigate the extensive data needs of DL models in medical imaging diagnosis with many mammography images. Like human doctors, LLMs can use textual information to enhance their diagnostic capabilities. By analyzing electronic health records and other textual information, an LLM can acquire more contextual knowledge, improving the accuracy of image diagnosis [348, 349, 350, 351]. LLMs can also accumulate a vast amount of medical knowledge and reasoning ability through the pretraining process, enabling them to apply existing knowledge and reasoning strategies when processing new image data, thus improving diagnostic efficiency and accuracy, even in cases of low image data availability [352, 353, 354].

In addition, various retrieval augmented generation (RAG) methods have been developed to search for documents from a knowledge corpus and generate responses by attaching them unconditionally or selectively to the input of the LLM [355, 356, 357]. In 2024, Jeong et al. [358] introduced self‐biomedical RAG (Self‐BioRAG), a reliable biomedical text framework that is specialized in generating explanations, retrieving domain‐specific documents, and self‐reflective generated responses. The experimental results for Self‐BioRAG demonstrated significant performance gains, with an average absolute improvement of 7.2% compared with those of state‐of‐the‐art open‐base models with parameter sizes of 7B or less [358].

Overall, the application of the RAG score to LLMs enables the models to act like medical experts do, utilizing information from retrieved documents and coded knowledge, thereby enhancing the capabilities of the biomedical and clinical domains.

A retrospective study by Andrea et al. in 2024 demonstrated that, based on breast imaging reports written in three languages, publicly available LLMs such as GPT‐4, GPT‐3.5, and Google Bard reached moderate agreement with the BI‐RADS category assignments given by human readers (AC1 values of 0.52, 0.48, and 0.42, respectively). However, their limitations in processing multilingual breast imaging reports have revealed their performance in complex clinical tasks needs improvement [359]. Additionally, in 2024, Vera et al. evaluated the performance of ChatGPT‐3.5 and GPT‐4 in clinical note analysis, guideline‐based Q&A, and patient management recommendations by searching medical literature analysis and retrieval systems online for relevant studies published before December 22, 2023. The results revealed the potential of LLMs in breast cancer patient care, with high accuracy in structured tasks but demonstrating issues regarding inconsistency and cue dependency, highlighting the importance of careful validation and continuous monitoring of these models [360]. Currently, state‐of‐the‐art models such as GPT‐4 and pathway language model 2 occupy a central position in healthcare AI innovation, showing significant potential for DBT screening in the early stages of breast cancer [359, 361, 362]. Incorporating LLMs into this field is expected to help health care professionals more accurately identify and classify breast lesions and shorten consultation times, potentially transforming the landscape of breast cancer diagnosis and screening.

However, the problem of the baselessness of LLMs in generating knowledge highlights the need to incorporate other technological tools to improve the accuracy and reliability of the generated information. Among these, the integration of knowledge graphs into LLMs provides one effective solution [363, 364, 365]. Knowledge graphs describe knowledge as a structured and decisive representation in the form of “head entity‐relation‐tail entity” triples; examples include Wikidata, yet another great ontology, and never‐ending language learning [366, 367, 368]. Knowledge graphs, an important subfield of knowledge engineering, can provide LLMs with structured medical knowledge and relationships to enhance their comprehension and reasoning capabilities, thus helping the models more accurately and comprehensively consider pathological features and diagnostic information when analyzing and interpreting medical images [369, 370, 371].

In 2023, Li et al. [372] designed a workflow centered on a DL model called the bidirectional long short‐term memory highway conditional random field. This workflow first establishes the structure of the knowledge graph for breast cancer diagnosis at the conceptual level and then searches for associations through bidirectional long short‐term memory while optimizing information flow with a highway network, which optimizes information flow and feature extraction, leading to significant improvements in performance [372]. In 2022, Zhang and Cao [373] combined knowledge graphs with natural language processing to simplify the extraction of breast cancer genetic features, using the Bhattacharyya distance index and Gini index for sample gene selection. These advances are expected to enrich the pathways for breast cancer gene extraction and significantly contribute to disease control and prevention [373].

In conclusion, DL models have broad application prospects in the field of DBT, and their potential to achieve significant advancements in diagnostic classification, lesion segmentation and detection, and medical image generation has been preliminarily verified in various studies. Through multisource domain migration, DL models are expected to integrate data from different sources better and achieve improved generalizability and diagnostic accuracy. AI technologies other than DL, including LLMs and knowledge graphs, are expected to accelerate the application and diffusion of DBT technologies in clinical settings.

### 6.3 Interpretability Challenges and Perspectives

One of the main challenges in applying DL and other AI models to DBT is the issue of model interpretability [374, 375, 376]. DL models are often regarded as “black boxes” [377] due to a lack of transparency in their decision‐making process [378, 379]. If a model makes a mistake, it is difficult for doctors to detect and correct it, potentially resulting in diagnostic errors [380].

Regulatory issues have a profound impact on the interpretability of DL models, and regulatory restrictions have made the development of tools and strategies for interpreting complex models of sophisticated AI a pressing need [381]. On the one hand, regulatory frameworks have driven the development of interpretability standards to ensure that AI meets transparency and compliance requirements in its applications [382]. On the other hand, regulatory requirements enhance model transparency, prompting developers to emphasize interpretability in the design and implementation process, enabling healthcare professionals to understand model decisions and thus increasing trust in AI [383]. Regulation also facilitates the validation and approval process by requiring developers to demonstrate evidence of explainability to gain approval for clinical applications, which drives the development of compliant technologies. Farah et al. [384] emphasized the importance of explainability in enhancing trust among healthcare stakeholders through a review of the existing literature. The specific tools proposed in the article include decision support flowcharts and evaluation methods that incorporate interpretability into the core of algorithm development to help understand how machine learning algorithms work [384].

Strategies for improving the interpretability of DL models applied to DBT include feature visualization, fusion of multiview feature fusion, model‐agnostic interpretation, and location annotation. Mohseni et al. [383] presented a multidisciplinary framework that integrates knowledge from different domains to support the design and evaluation of explainable AI (XAI) in clinical workflows. The article categorizes the design goals and evaluation methods of XAI, emphasizing how interpretable tools can be practically applied to enhance physicians' trust and willingness to use them [385]. Lu et al. [386] enhanced the model's application in clinical trial management by employing a selective classification approach in conjunction with a hierarchical interaction network that ensured the retention of prediction decisions at low confidence, thereby enhancing physicians’ trust and willingness to use model predictions. The results of the study showed that the method significantly improved the accuracy and interpretability of clinical trial approval prediction [386]. Improving interpretability can be achieved by introducing innovative neural network architectures and visualization techniques, such as models based on attention mechanisms and Grad‐CAM. The introduction of the attention mechanism enhanced model interpretability, allowing networks based on this mechanism to represent the significance of features at different spatial locations and guide the information visible in other parts of the network [387]. Fusion of multiview features involves combining image information from different angles and levels and enhancing the interpretability and reliability of the model by integrating features from different viewpoints, thereby making the model's decision more clinically meaningful.

In 2023, Zhong et al. [388] proposed a multiview fusion network with local‐global dual‐path transformer architecture, for mammography‐based breast density classification in breast cancer screening, achieving AUCs of 96.73 and 91.12% on two publicly available mammography datasets, CBIS‐DDSM and INbreast, respectively. The designed fusion model utilizes information from multiple views more efficiently than existing models do, outperforming baseline and state‐of‐the‐art methods [388]. In 2016, Gal and Ghahramani [389] developed a new theoretical framework that treats dropout training in deep neural networks as approximate Bayesian inference in a deep Gaussian process.

Model‐agnostic interpretation methods, such as LIME and Shapley additive explanations (SHAPs), can reveal the detailed contributions of individual features to the prediction results of a DL model [390, 391, 392]. In 2022, Ma et al. [216] built an interpretable machine learning model to distinguish the molecular subtypes of breast cancer, using the SHAP technique to identify important features for predicting the molecular subtypes from many imaging signs. The application of these explanatory methods in DBT can better elucidate the decision‐making process of the model for each image segment, identify key lesion features, and enhance physicians’ trust in the model's predictions, thereby increasing the clinical application value of DBT [393].

Another intuitive and enhanced interpretability approach is lesion localization annotation [394]. This method can reveal the spatial locations of key features in the model's decision‐making process, aiding imaging physicians in understanding and validating the model's diagnostic results, thereby improving trust and clinical usability in the model. The noninterpretability of DL models remains the most significant barrier to their clinical deployment, highlighting the urgent need for models with better interpretability [395].

## 7 Conclusion

This review comprehensively analyzes the applications, challenges, and future directions related to DL technology in the field of DBT. First, we analyzed the current status of DBTs, including their principles and applications. Next, we summarize the functions and applications of DL in the treatment of breast diseases and classify the DL models into three main categories. Additionally, we explore the application of DL in other areas of DBT and other AI techniques in addition to DL in early DBT screening, summarizing publicly accessible databases. Finally, we address the challenges and future research directions in the application of DL to DBT, summarize the potential of knowledge graph‐ and LLM‐based applications in DBT, and lay a foundation for advancing DL‐based early DBT screening applications. Our work highlights the significant promise of DL applications in DBT and outlines future research trajectories.

## Author Contributions

R.Y.W.: Data curation, investigation, and writing—original draft. F.X.C.: Data curation, investigation, and writing—original draft. H.M.C.: Data curation, investigation, and writing—original draft. C.X.L.: Investigation, methodology, and visualization. J.C.S.: Investigation, methodology, and visualization. Y.T.W.: Investigation, methodology, and visualization. L.X.M.: Investigation, methodology, and visualization. X.Q.H.: Investigation, methodology, and visualization. M.W.: Investigation, methodology, and visualization. J.W.: Investigation, methodology, and visualization. Q.Z.: Conceptualization, funding acquisition, project administration, supervision, and writing—review and editing. J.W.S.: Conceptualization, funding acquisition, project administration, supervision, and writing—review and editing. J.Y.P.: Conceptualization, funding acquisition, project administration, supervision, and writing—review and editing. All authors have read and approved the final manuscript.

## Ethics Statement

The authors have nothing to report.

## Conflicts of Interest

The authors declare no conflicts of interest.

## Supporting information




Supporting Information

## Data Availability

The authors have nothing to report.

## References

[1] S. Lei , R. Zheng , S. Zhang , et al., “Global Patterns of Breast Cancer Incidence and Mortality: A Population‐based Cancer Registry Data Analysis From 2000 to 2020,” Cancer Communications (London) 41, no. 11 (2021): 1183–1194. 10.1002/cac2.12207 PMC8626596 34399040

[2] A. Jemal , F. Bray , M. M. Center , J. Ferlay , E. Ward , and D. Forman , “Global Cancer Statistics,” CA: A Cancer Journal for Clinicians 61, no. 2 (2011): 69–90. 21296855 10.3322/caac.20107

[3] F. Bray , J. Ferlay , I. Soerjomataram , R. L. Siegel , L. A. Torre , and A. Jemal , “Global Cancer Statistics 2018: GLOBOCAN Estimates of Incidence and Mortality Worldwide for 36 Cancers in 185 Countries,” CA: A Cancer Journal for Clinicians 68, no. 6 (2018): 394–424. 30207593 10.3322/caac.21492

[4] J. Huang , P. S. Chan , V. Lok , et al., “Global Incidence and Mortality of Breast Cancer: A Trend Analysis,” Aging (Albany NY) 13, no. 4 (2021): 5748–5803. 33592581 10.18632/aging.202502 PMC7950292

[5] F. Bray , M. Laversanne , H. Sung , et al., “Global Cancer Statistics 2022: GLOBOCAN Estimates of Incidence and Mortality Worldwide for 36 Cancers in 185 Countries,” CA: A Cancer Journal for Clinicians 74, no. 3 (2024): 229–263. 38572751 10.3322/caac.21834

[6] R. L. Siegel , K. D. Miller , H. E. Fuchs , and A. Jemal , “Cancer Statistics, 2022,” CA: A Cancer Journal for Clinicians 72, no. 1 (2022): 7–33. 35020204 10.3322/caac.21708

[7] S. Chen , Z. Cao , K. Prettner , et al., “Estimates and Projections of the Global Economic Cost of 29 Cancers in 204 Countries and Territories from 2020 to 2050,” JAMA Oncology 9, no. 4 (2023): 465–472. 36821107 10.1001/jamaoncol.2022.7826 PMC9951101

[8] N. Zacharakis , H. Chinnasamy , M. Black , et al., “Immune Recognition of Somatic Mutations Leading to Complete Durable Regression in Metastatic Breast Cancer,” Nature Medicine 24, no. 6 (2018): 724–730. 10.1038/s41591-018-0040-8 PMC6348479 29867227

[9] L. Chen , L. Yang , L. Yao , et al., “Characterization of PIK3CA and PIK3R1 Somatic Mutations in Chinese Breast Cancer Patients,” Nature Communications 9, no. 1 (2018): 1357. 10.1038/s41467-018-03867-9 PMC5893593 29636477

[10] L. Garcia‐Martinez , Y. Zhang , Y. Nakata , H. L. Chan , and L. Morey , “Epigenetic Mechanisms in Breast Cancer Therapy and Resistance,” Nature Communications 12, no. 1 (2021): 1786. 10.1038/s41467-021-22024-3 PMC7979820 33741974

[11] Breast Cancer Association C , L. Dorling , S. Carvalho , et al., Breast Cancer Association C , “Breast Cancer Risk Genes—Association Analysis in More Than 113,000 Women,” New England Journal of Medicine 384, no. 5 (2021): 428–439. 33471991 10.1056/NEJMoa1913948 PMC7611105

[12] S. S. Buys , J. F. Sandbach , A. Gammon , et al., “A Study of Over 35,000 Women With Breast Cancer Tested With a 25‐gene Panel of Hereditary Cancer Genes,” Cancer 123, no. 10 (2017): 1721–1730. 28085182 10.1002/cncr.30498

[13] C. Hu , S. N. Hart , R. Gnanaolivu , et al., “A Population‐Based Study of Genes Previously Implicated in Breast Cancer,” New England Journal of Medicine 384, no. 5 (2021): 440–451. 33471974 10.1056/NEJMoa2005936 PMC8127622

[14] J. Sun , H. Meng , L. Yao , et al., “Germline Mutations in Cancer Susceptibility Genes in a Large Series of Unselected Breast Cancer Patients,” Clinical Cancer Research 23, no. 20 (2017): 6113–6119. 28724667 10.1158/1078-0432.CCR-16-3227

[15] J. D. Yager and N. E. Davidson , “Estrogen Carcinogenesis in Breast Cancer,” New England Journal of Medicine 354, no. 3 (2006): 270–282. 16421368 10.1056/NEJMra050776

[16] American College of Obstetricians and Gynecologists . “Hereditary Cancer Syndromes and Risk Assessment: ACOG COMMITTEE OPINION SUMMARY, Number 793,” Obstetrics and Gynecology 2019;134(6):1366–1367. 31764755 10.1097/AOG.0000000000003563

[17] B. Xia , Q. Sheng , K. Nakanishi , et al., “Control of BRCA2 Cellular and Clinical Functions by a Nuclear Partner, PALB2,” Molecular Cell 22, no. 6 (2006): 719–729. 16793542 10.1016/j.molcel.2006.05.022

[18] A. McTiernan , “Behavioral Risk Factors in Breast Cancer: Can Risk be Modified?,” The Oncologist 8, no. 4 (2003): 326–334. 12897329 10.1634/theoncologist.8-4-326

[19] C. Coleman , “Early Detection and Screening for Breast cancer,” Seminars in Oncology Nursing. (Elsevier, 2017): 141–155. 10.1016/j.soncn.2017.02.009 28365057

[20] H. Qiu , S. Cao , and R. Xu , “Cancer Incidence, Mortality, and Burden in China: A Time‐trend Analysis and Comparison With the United States and United Kingdom Based on the Global Epidemiological Data Released in 2020,” Cancer Communications (London) 41, no. 10 (2021): 1037–1048. 10.1002/cac2.12197 PMC8504144 34288593

[21] I. Sechopoulos , J. Teuwen , and R. Mann , “Artificial Intelligence for Breast Cancer Detection in Mammography and Digital Breast Tomosynthesis: State of the Art,” Seminars in Cancer Biology 72 (2021): 214–225. 32531273 10.1016/j.semcancer.2020.06.002

[22] A. Chong , S. P. Weinstein , E. S. McDonald , and E. F. Conant , “Digital Breast Tomosynthesis: Concepts and Clinical Practice,” Radiology 292, no. 1 (2019): 1–14. 31084476 10.1148/radiol.2019180760 PMC6604796

[23] L. R. Cochon , C. S. Giess , and R. Khorasani , “Comparing Diagnostic Performance of Digital Breast Tomosynthesis and Full‐Field Digital Mammography,” Journal of the American College of Radiology 17, no. 8 (2020): 999–1003. 32068009 10.1016/j.jacr.2020.01.010

[24] E. O. Cohen , O. O. Weaver , H. H. Tso , K. E. Gerlach , and J. W. T. Leung , “Breast Cancer Screening via Digital Mammography, Synthetic Mammography, and Tomosynthesis,” American Journal of Preventive Medicine 58, no. 3 (2020): 470–472. 31732323 10.1016/j.amepre.2019.09.016

[25] X. Qian , J. Pei , H. Zheng , et al., “Prospective Assessment of Breast Cancer Risk From Multimodal Multiview Ultrasound Images via Clinically Applicable Deep Learning,” Nature Biomedical Engineering 5, no. 6 (2021): 522–532. 10.1038/s41551-021-00711-2 33875840

[26] T. Hovda , A. S. Holen , K. Lang , et al., “Interval and Consecutive Round Breast Cancer After Digital Breast Tomosynthesis and Synthetic 2D Mammography versus Standard 2D Digital Mammography in BreastScreen Norway,” Radiology 294, no. 2 (2020): 256–264. 31821118 10.1148/radiol.2019191337

[27] E. R. Myers , P. Moorman , J. M. Gierisch , et al., “Benefits and Harms of Breast Cancer Screening: A Systematic Review,” Jama 314, no. 15 (2015): 1615–1634. 26501537 10.1001/jama.2015.13183

[28] R. Murakami , N. Uchiyama , H. Tani , T. Yoshida , and S. Kumita , “Comparative Analysis Between Synthetic Mammography Reconstructed From Digital Breast Tomosynthesis and Full‐field Digital Mammography for Breast Cancer Detection and Visibility,” European Journal of Radiology Open 7 (2020): 100207. 33102630 10.1016/j.ejro.2019.12.001 PMC7569412

[29] S. Weigel , W. Heindel , H. W. Hense , et al., “Breast Density and Breast Cancer Screening With Digital Breast Tomosynthesis: A TOSYMA Trial Subanalysis,” Radiology 306, no. 2 (2023): e221006. 36194110 10.1148/radiol.221006

[30] W. Wang , L. Zhang , J. Sun , Q. Zhao , and J. Shuai , “Predicting the Potential human lncRNA‐miRNA Interactions Based on Graph Convolution Network With Conditional Random Field,” Briefings in Bioinformatics 23, no. 6 (2022): bbac463. 36305458 10.1093/bib/bbac463

[31] J. Lei , P. Yang , L. Zhang , Y. Wang , and K. Yang , “Diagnostic Accuracy of Digital Breast Tomosynthesis versus Digital Mammography for Benign and Malignant Lesions in Breasts: A Meta‐analysis,” European Radio 24 (2014): 595–602. 10.1007/s00330-013-3012-x 24121712

[32] P. Skaane , R. Gullien , H. Bjorndal , et al., “Digital Breast Tomosynthesis (DBT): Initial Experience in a Clinical Setting,” Acta Radiologica 53, no. 5 (2012): 524–529. 22593120 10.1258/ar.2012.120062

[33] Y. Choi , O. H. Woo , H. S. Shin , K. R. Cho , B. K. Seo , and G. Y. Choi , “Quantitative Analysis of Radiation Dosage and Image Quality Between Digital Breast Tomosynthesis (DBT) With Two‐dimensional Synthetic Mammography and Full‐field Digital Mammography (FFDM),” Clinical Imaging 55 (2019): 12–17. 30703693 10.1016/j.clinimag.2019.01.014

[34] D. B. Kopans , “Time for Change in Digital Breast Tomosynthesis Research,” Radiology 302, no. 2 (2022): 293–294. 34751614 10.1148/radiol.2021204697

[35] A. M. Mota , J. Mendes , and N. Matela , “Digital Breast Tomosynthesis: Towards Dose Reduction Through Image Quality Improvement,” Journal of Imaging 9, no. 6 (2023): 119. 37367467 10.3390/jimaging9060119 PMC10299507

[36] R. G. Roth , A. D. Maidment , S. P. Weinstein , S. O. Roth , and E. F. Conant , “Digital Breast Tomosynthesis: Lessons Learned From Early Clinical Implementation,” Radiographics 34, no. 4 (2014): E89–E102. 25019451 10.1148/rg.344130087 PMC4319526

[37] E. Dhamija , M. Gulati , S. V. S. Deo , A. Gogia , and S. Hari , “Digital Breast Tomosynthesis: An Overview,” Indian Journal of Surgical Oncology 12, no. 2 (2021): 315–329. 34295076 10.1007/s13193-021-01310-y PMC8272763

[38] A. Rodriguez‐Ruiz , J. Teuwen , S. Vreemann , et al., “New Reconstruction Algorithm for Digital Breast Tomosynthesis: Better Image Quality for Humans and Computers,” Acta Radiologica 59, no. 9 (2018): 1051–1059. 29254355 10.1177/0284185117748487 PMC6088454

[39] R. Zeng , A. Badano , and K. J. Myers , “Optimization of Digital Breast Tomosynthesis (DBT) Acquisition Parameters for human Observers: Effect of Reconstruction Algorithms,” Physics in Medicine and Biology 62, no. 7 (2017): 2598–2611. 28151728 10.1088/1361-6560/aa5ddc PMC5541400

[40] M. Ertas , I. Yildirim , M. Kamasak , and A. Akan , “Digital Breast Tomosynthesis Image Reconstruction Using 2D and 3D Total Variation Minimization,” Biomedical Engineering Online [Electronic Resource] 12 (2013): 112. 24172584 10.1186/1475-925X-12-112 PMC4228494

[41] H. R. Peppard , B. E. Nicholson , C. M. Rochman , J. K. Merchant , R. C. Mayo 3rd , and J. A. Harvey , “Digital Breast Tomosynthesis in the Diagnostic Setting: Indications and Clinical Applications,” Radiographics 35, no. 4 (2015): 975–990. 26024062 10.1148/rg.2015140204

[42] S. Vedantham , L. Shi , K. E. Michaelsen , et al., “Digital Breast Tomosynthesis Guided Near Infrared Spectroscopy: Volumetric Estimates of Fibroglandular Fraction and Breast Density From Tomosynthesis Reconstructions,” Biomedical Physics & Engineering Express 1, no. 4 (2015): 045202. 26941961 10.1088/2057-1976/1/4/045202 PMC4771071

[43] V. Magni , A. Cozzi , S. Schiaffino , A. Colarieti , and F. Sardanelli , “Artificial Intelligence for Digital Breast Tomosynthesis: Impact on Diagnostic Performance, Reading Times, and Workload in the Era of Personalized Screening,” European Journal of Radiology 158 (2023): 110631. 36481480 10.1016/j.ejrad.2022.110631

[44] F. Shaheen , B. Verma , and M. Asafuddoula , “Impact of Automatic Feature Extraction in Deep Learning Architecture,” In: 2016 International conference on digital image computing: techniques and applications (DICTA) . IEEE; 2016:1–8.

[45] G. Farias , S. Dormido‐Canto , J. Vega , et al., “Automatic Feature Extraction in Large Fusion Databases by Using Deep Learning Approach,” Fusion Engineering and Design 112 (2016): 979–983.

[46] M. M. Adnan , M. S. M. Rahim , A. Rehman , Z. Mehmood , T. Saba , and R. A. Naqvi , “Automatic Image Annotation Based on Deep Learning Models: A Systematic Review and Future Challenges,” IEEE Access 9 (2021): 50253–50264.

[47] A. Gordo , J. Almazan , J. Revaud , and D. Larlus , “End‐to‐end Learning of Deep Visual Representations for Image Retrieval,” International Journal of Computer Vision 124, no. 2 (2017): 237–254.

[48] Z. Wang , L. Zhang , X. Shu , Q. Lv , and Z. Yi , “An End‐to‐end Mammogram Diagnosis: A New Multi‐instance and Multiscale Method Based on Single‐image Feature,” IEEE Transactions on Cognitive and Developmental Systems 13, no. 3 (2020): 535–545.

[49] L. Wang , Q. He , X. Wang , et al., “Multi‐criterion Decision Making‐based Multi‐channel Hierarchical Fusion of Digital Breast Tomosynthesis and Digital Mammography for Breast Mass Discrimination,” Knowledge‐Based Systems 228 (2021): 107303.

[50] C. Yu and J. Wang , “Data Mining and Mathematical Models in Cancer Prognosis and Prediction,” Medical Review (2021) 2, no. 3 (2022): 285–307. 37724193 10.1515/mr-2021-0026 PMC10388766

[51] R. A. Welikala , P. Remagnino , J. H. Lim , et al., “Automated Detection and Classification of Oral Lesions Using Deep Learning for Early Detection of Oral Cancer,” IEEE Access 8 (2020): 132677–132693.

[52] D. Crosby , S. Bhatia , K. M. Brindle , et al., “Early Detection of Cancer,” Science 375, no. 6586 (2022): eaay9040. 35298272 10.1126/science.aay9040

[53] A. Alsadoon , G. Al‐Naymat , A. H. Osman , B. Alsinglawi , M. Maabreh , and M. R. Islam , “DFCV: A Framework for Evaluation Deep Learning in Early Detection and Classification of Lung Cancer,” Multimedia Tools and Applications 82, no. 28 (2023): 44387–44430.

[54] R. Ricciardi , G. Mettivier , M. Staffa , et al., “A Deep Learning Classifier for Digital Breast Tomosynthesis,” Physical Medicine 83 (2021): 184–193. 10.1016/j.ejmp.2021.03.021 33798904

[55] D. Fornvik , S. Borgquist , M. Larsson , S. Zackrisson , and I. Skarping , “Deep Learning Analysis of Serial Digital Breast Tomosynthesis Images in a Prospective Cohort of Breast Cancer Patients Who Received Neoadjuvant Chemotherapy,” European Journal of Radiology 178 (2024): 111624. 39029241 10.1016/j.ejrad.2024.111624

[56] W. Lee , H. Lee , H. Lee , E. K. Park , H. Nam , and T. Kooi , “Transformer‐based Deep Neural Network for Breast Cancer Classification on Digital Breast Tomosynthesis Images,” Radiology: Artificial Intelligence 5, no. 3 (2023): e220159. 37293346 10.1148/ryai.220159 PMC10245183

[57] C. D. Lehman , R. D. Wellman , D. S. Buist , et al., “Diagnostic Accuracy of Digital Screening Mammography with and without Computer‐Aided Detection,” JAMA Internal Medicine 175, no. 11 (2015): 1828–1837. 26414882 10.1001/jamainternmed.2015.5231 PMC4836172

[58] K. J. Geras , R. M. Mann , and L. Moy , “Artificial Intelligence for Mammography and Digital Breast Tomosynthesis: Current Concepts and Future Perspectives,” Radiology 293, no. 2 (2019): 246–259. 31549948 10.1148/radiol.2019182627 PMC6822772

[59] J. H. Yoon , F. Strand , P. A. T. Baltzer , et al., “Standalone AI for Breast Cancer Detection at Screening Digital Mammography and Digital Breast Tomosynthesis: A Systematic Review and Meta‐Analysis,” Radiology 307, no. 5 (2023): e222639. 37219445 10.1148/radiol.222639 PMC10315526

[60] J. Bai , R. Posner , T. Wang , C. Yang , and S. Nabavi , “Applying Deep Learning in Digital Breast Tomosynthesis for Automatic Breast Cancer Detection: A Review,” Medical Image Analysis 71 (2021): 102049. 33901993 10.1016/j.media.2021.102049

[61] J. Zhang , J. Wu , X. S. Zhou , F. Shi , and D. Shen , “Recent Advancements in Artificial Intelligence for Breast Cancer: Image Augmentation, Segmentation, Diagnosis, and Prognosis Approaches,” Seminars in Cancer Biology 96 (2023): 11–25. 37704183 10.1016/j.semcancer.2023.09.001

[62] M. J. Yaffe , “Detectors for Digital Mammography,” Digital Mammography (2010): 13–31.

[63] I. K. Maitra , S. Nag , and S. K. Bandyopadhyay , “Technique for Preprocessing of Digital Mammogram,” Computer Methods and Programs in Biomedicine 107, no. 2 (2012): 175–188. 21669471 10.1016/j.cmpb.2011.05.007

[64] E. F. Conant , E. F. Beaber , B. L. Sprague , et al., “Breast Cancer Screening Using Tomosynthesis in Combination With Digital Mammography Compared to Digital Mammography Alone: A Cohort Study Within the PROSPR Consortium,” Breast Cancer Research and Treatment 156, no. 1 (2016): 109–116. 26931450 10.1007/s10549-016-3695-1 PMC5536249

[65] S. K. Yang , W. K. Moon , N. Cho , et al., “Screening Mammography‐detected Cancers: Sensitivity of a Computer‐aided Detection System Applied to Full‐field Digital Mammograms,” Radiology 244, no. 1 (2007): 104–111. 17507722 10.1148/radiol.2441060756

[66] J. S. The , K. J. Schilling , J. W. Hoffmeister , E. Friedmann , R. McGinnis , and R. G. Holcomb , “Detection of Breast Cancer With Full‐field Digital Mammography and Computer‐aided Detection,” Ajr American Journal of Roentgenology 192, no. 2 (2009): 337–340. 19155392 10.2214/AJR.07.3884

[67] S. P. Poplack , A. N. Tosteson , M. R. Grove , W. A. Wells , and P. A. Carney , “Mammography in 53,803 Women From the New Hampshire Mammography Network,” Radiology 217, no. 3 (2000): 832–840. 11110951 10.1148/radiology.217.3.r00dc33832

[68] H. Sung , J. Ferlay , R. L. Siegel , et al., “Global Cancer Statistics 2020: GLOBOCAN Estimates of Incidence and Mortality Worldwide for 36 Cancers in 185 Countries,” CA: A Cancer Journal for Clinicians 71, no. 3 (2021): 209–249. 33538338 10.3322/caac.21660

[69] P. Skaane , S. Sebuodegard , A. I. Bandos , et al., “Performance of Breast Cancer Screening Using Digital Breast Tomosynthesis: Results From the Prospective Population‐based Oslo Tomosynthesis Screening Trial,” Breast Cancer Research and Treatment 169, no. 3 (2018): 489–496. 29429017 10.1007/s10549-018-4705-2

[70] R. E. Hendrick , “Radiation Doses and Risks in Breast Screening,” Journal of Breast Imaging 2, no. 3 (2020): 188–200. 38424982 10.1093/jbi/wbaa016

[71] I. Sechopoulos , “A Review of Breast Tomosynthesis. Part II. Image Reconstruction, Processing and Analysis, and Advanced Applications,” Medical Physics 40, no. 1 (2013): 014302. 23298127 10.1118/1.4770281 PMC3548896

[72] Y. Z. Tang , A. Al‐Arnawoot , and A. Alabousi , “The Impact of Slice Thickness on Diagnostic Accuracy in Digital Breast Tomosynthesis,” Canadian Association of Radiologists Journal 73, no. 3 (2022): 535–541. 35193417 10.1177/08465371211068200

[73] K. Nakashima , T. Uematsu , T. Itoh , et al., “Comparison of Visibility of Circumscribed Masses on Digital Breast Tomosynthesis (DBT) and 2D Mammography: Are Circumscribed Masses Better Visualized and Assured of Being Benign on DBT?,” European Radiology 27, no. 2 (2017): 570–577. 27236817 10.1007/s00330-016-4420-5

[74] J. Krammer , S. Zolotarev , I. Hillman , et al., “Evaluation of a New Image Reconstruction Method for Digital Breast Tomosynthesis: Effects on the Visibility of Breast Lesions and Breast Density,” Bjr 92, no. 1103 (2019): 20190345. 31453718 10.1259/bjr.20190345 PMC6849672

[75] J. M. Park , E. A. Franken Jr. , M. Garg , L. L. Fajardo , and L. T. Niklason , “Breast Tomosynthesis: Present Considerations and Future Applications,” Radiographics 27, no. suppl_1 (2007): S231–S240. 18180229 10.1148/rg.27si075511

[76] R. E. Sharpe Jr , S. Venkataraman , J. Phillips , et al., “Increased Cancer Detection Rate and Variations in the Recall Rate Resulting From Implementation of 3D Digital Breast Tomosynthesis Into a Population‐based Screening Program,” Radiology 278, no. 3 (2016): 698–706. 26458206 10.1148/radiol.2015142036 PMC4770944

[77] J. H. Yoon , E. K. Kim , G. R. Kim , et al., “Comparing Recall Rates Following Implementation of Digital Breast Tomosynthesis to Synthetic 2D Images and Digital Mammography on Women With Breast‐conserving Surgery,” European Radiology 30, no. 11 (2020): 6072–6079. 32529566 10.1007/s00330-020-06992-6

[78] H. J. Teertstra , C. E. Loo , M. A. van den Bosch , et al., “Breast Tomosynthesis in Clinical Practice: Initial Results,” European Radiology 20, no. 1 (2010): 16–24. 19657655 10.1007/s00330-009-1523-2

[79] A. Vourtsis and W. A. Berg , “Breast Density Implications and Supplemental Screening,” European Radiology 29, no. 4 (2019): 1762–1777. 30255244 10.1007/s00330-018-5668-8 PMC6420861

[80] X.‐A. Phi , A. Tagliafico , N. Houssami , M. J. Greuter , and G. H. de Bock , “Digital Breast Tomosynthesis for Breast Cancer Screening and Diagnosis in Women With Dense Breasts–a Systematic Review and Meta‐analysis,” BMC Cancer 18 (2018): 1–9. 29615072 10.1186/s12885-018-4263-3 PMC5883365

[81] I. Hadadi , W. Rae , J. Clarke , M. McEntee , and E. Ekpo , “Breast Cancer Detection: Comparison of Digital Mammography and Digital Breast Tomosynthesis Across Non‐dense and Dense Breasts,” Radiography (London) 27, no. 4 (2021): 1027–1032. 10.1016/j.radi.2021.04.002 33906803

[82] G. Gennaro , S. Del Genio , G. Manco , and F. Caumo , “Phantom‐based Analysis of Variations in Automatic Exposure Control Across Three Mammography Systems: Implications for Radiation Dose and Image Quality in Mammography, DBT, and CEM,” European Radiology Experimental 8, no. 1 (2024): 49. 38622388 10.1186/s41747-024-00447-z PMC11018565

[83] R. M. Ali , A. England , A. K. Tootell , and P. Hogg , “Radiation Dose From Digital Breast Tomosynthesis Screening–A Comparison With Full Field Digital Mammography,” Journal of Medical Imaging and Radiation Sciences 51, no. 4 (2020): 599–603. 32943362 10.1016/j.jmir.2020.08.018

[84] N. Houssami , D. Bernardi , and G. Gennaro , “Radiation Dose With Digital Breast Tomosynthesis Compared to Digital Mammography: Per‐view Analysis,” European Radio 28 (2018): 573–581. 10.1007/s00330-017-5024-4 28819862

[85] B. Barufaldi , H. Schiabel , and A. D. A. Maidment , “Design and Implementation of a Radiation Dose Tracking and Reporting System for Mammography and Digital Breast Tomosynthesis,” Physical Medicine 58 (2019): 131–140. 10.1016/j.ejmp.2019.02.011 PMC6404780 30824144

[86] Y. Shoshan , R. Bakalo , F. Gilboa‐Solomon , et al., “Artificial Intelligence for Reducing Workload in Breast Cancer Screening With Digital Breast Tomosynthesis,” Radiology 303, no. 1 (2022): 69–77. 35040677 10.1148/radiol.211105

[87] G. J. Partridge , I. Darker , J. J. James , et al., “How Long Does It Take to Read a Mammogram? Investigating the Reading Time of Digital Breast Tomosynthesis and Digital Mammography,” European Journal of Radiology 177 (2024): 111535. 38852330 10.1016/j.ejrad.2024.111535

[88] S. A. Abdullah Suhaimi , A. Mohamed , M. Ahmad , and K. K. Chelliah , “Effects of Reduced Compression in Digital Breast Tomosynthesis on Pain, Anxiety, and Image Quality,” Malaysian Journal of Medical Sciences 22, no. 6 (2015): 40–46. PMC5295750 28223884

[89] J. X. Hu , C. F. Zhao , S. L. Wang , et al., “Acute Pancreatitis: A Review of Diagnosis, Severity Prediction and Prognosis Assessment From Imaging Technology, Scoring System and Artificial Intelligence,” World Journal of Gastroenterology 29, no. 37 (2023): 5268–5291. 37899784 10.3748/wjg.v29.i37.5268 PMC10600804

[90] T. Lefevre and L. Tournois , “Artificial Intelligence and Diagnostics in Medicine and Forensic Science,” Diagnostics (Basel) 13, no. 23 (2023): 3554. 38066795 10.3390/diagnostics13233554 PMC10706664

[91] M. Moor , O. Banerjee , Z. S. H. Abad , et al., “Foundation Models for Generalist Medical Artificial Intelligence,” Nature 616, no. 7956 (2023): 259–265. 37045921 10.1038/s41586-023-05881-4

[92] H. Chi , H. Chen , R. Wang , et al., “Proposing New Early Detection Indicators for Pancreatic Cancer: Combining Machine Learning and Neural Networks for Serum miRNA‐based Diagnostic Model,” Frontiers in Oncology 13 (2023): 1244578. 37601672 10.3389/fonc.2023.1244578 PMC10437932

[93] R. Li , Y. Guo , Z. Zhao , et al., “MRI‐based Two‐stage Deep Learning Model for Automatic Detection and Segmentation of Brain Metastases,” European Radiology 33, no. 5 (2023): 3521–3531. 36695903 10.1007/s00330-023-09420-7

[94] D. T. Hoang , G. Dinstag , E. D. Shulman , et al., “A Deep‐learning Framework to Predict Cancer Treatment Response From Histopathology Images Through Imputed Transcriptomics,” Nature Cancer 5, no. 9 (2024): 1305–1317. 38961276 10.1038/s43018-024-00793-2 PMC12413935

[95] E. J. Hwang , W. G. Jeong , P. M. David , M. Arentz , M. Ruhwald , and S. H. Yoon , “AI for Detection of Tuberculosis: Implications for Global Health,” Radiology: Artificial Intelligence 6, no. 2 (2024): e230327. 38197795 10.1148/ryai.230327 PMC10982823

[96] Y. Ren , X. Liu , J. Ge , et al., “Ipsilateral Lesion Detection Refinement for Tomosynthesis,” Ieee Transactions on Medical Imaging 42, no. 10 (2023): 3080–3090. 37227903 10.1109/TMI.2023.3280135 PMC11033619

[97] E. P. V. Le , Y. Wang , Y. Huang , S. Hickman , and F. J. Gilbert , “Artificial Intelligence in Breast Imaging,” Clinical Radiology 74, no. 5 (2019): 357–366. 30898381 10.1016/j.crad.2019.02.006

[98] T. Uematsu , K. Nakashima , T. L. Harada , H. Nasu , and T. Igarashi , “Comparisons Between Artificial Intelligence Computer‐aided Detection Synthesized Mammograms and Digital Mammograms When Used Alone and in Combination With Tomosynthesis Images in a Virtual Screening Setting,” Japanese Journal of Radiology 41, no. 1 (2023): 63–70. 36068450 10.1007/s11604-022-01327-5 PMC9813079

[99] E. Yagis , A. G. S. De Herrera , and L. Citi , “Generalization Performance of Deep Learning Models in Neurodegenerative Disease Classification,” In: 2019 IEEE international conference on bioinformatics and biomedicine (BIBM) . IEEE; 2019:1692–1698.

[100] S. Minaee , Y. Boykov , F. Porikli , A. Plaza , N. Kehtarnavaz , and D. Terzopoulos , “Image Segmentation Using Deep Learning: A Survey,” Ieee Transactions on Pattern Analysis and Machine Intelligence 44, no. 7 (2021): 3523–3542. 10.1109/TPAMI.2021.3059968 33596172

[101] C. Chen , C. Qin , H. Qiu , et al., “Deep Learning for Cardiac Image Segmentation: A Review,” Frontiers in Cardiovascular Medicine 7 (2020): 25. 32195270 10.3389/fcvm.2020.00025 PMC7066212

[102] Z. Q. Zhao , P. Zheng , S. T. Xu , and X. Wu , “Object Detection with Deep Learning: A Review,” IEEE Transactions on Neural Networks and Learning Systems 30, no. 11 (2019): 3212–3232. 30703038 10.1109/TNNLS.2018.2876865

[103] J. Fan , X. Cao , Z. Xue , P.‐T. Yap , and D. Shen , “Adversarial Similarity Network for Evaluating Image Alignment in Deep Learning Based Registration,” In: Medical Image Computing and Computer Assisted Intervention–MICCAI 2018: 21st International Conference, Granada, Spain, September 16–20, 2018, Proceedings, Part I . Springer; 2018:739–746. 10.1007/978-3-030-00928-1_83 PMC6322551 30627709

[104] J. Teuwen , N. Moriakov , C. Fedon , et al., “Deep Learning Reconstruction of Digital Breast Tomosynthesis Images for Accurate Breast Density and Patient‐specific Radiation Dose Estimation,” Medical Image Analysis 71 (2021): 102061. 33910108 10.1016/j.media.2021.102061

[105] T. Gomi , Y. Kijima , T. Kobayashi , and Y. Koibuchi , “Evaluation of a Generative Adversarial Network to Improve Image Quality and Reduce Radiation‐Dose During Digital Breast Tomosynthesis,” Diagnostics (Basel) 12, no. 2 (2022): 495. 35204582 10.3390/diagnostics12020495 PMC8871529

[106] J. Reifman and E. E. Feldman , “Multilayer Perceptron for Nonlinear Programming,” Computers & Operations Research 29, no. 9 (2002): 1237–1250.

[107] T. Kim and T. Adali , “Fully Complex Multi‐layer Perceptron Network for Nonlinear Signal Processing,” Journal of Signal Processing Systems 32 (2002): 29–43.

[108] Y. LeCun , L. Bottou , Y. Bengio , and P. Haffner , “Gradient‐based Learning Applied to Document Recognition,” Proceedings of the Ieee 86, no. 11 (1998): 2278–2324.

[109] P.‐S. Zhu , Y.‐R. Zhang , J.‐Y. Ren , et al., “Ultrasound‐based Deep Learning Using the VGGNet Model for the Differentiation of Benign and Malignant Thyroid Nodules: A Meta‐analysis,” Frontiers in Oncology 12 (2022): 944859. 36249056 10.3389/fonc.2022.944859 PMC9554631

[110] N. Zakaria and Y. M. M. Hassim , “Improved Image Classification Task Using Enhanced Visual Geometry Group of Convolution Neural Networks,” International Journal on Informatics Visualization 7, no. 4 (2023): 2498–2505.

[111] N. Veni and J. Manjula , “High‐performance Visual Geometric Group Deep Learning Architectures for MRI Brain Tumor Classification,” The Journal of Supercomputing 78, no. 10 (2022): 12753–12764.

[112] N. Zakaria and Y. M. Mohmad Hassim , “A Review Study of the Visual Geometry Group Approaches for Image Classification,” Journal of Applied Science, Technology and Computing 1, no. 1 (2024): 14–28.

[113] L. Balagourouchetty , J. K. Pragatheeswaran , B. Pottakkat , and R. Govindarajalou , “GoogLeNet‐Based Ensemble FCNet Classifier for Focal Liver Lesion Diagnosis,” IEEE Journal of Biomedical and Health Informatics 24, no. 6 (2020): 1686–1694. 31545749 10.1109/JBHI.2019.2942774

[114] S. M. Sam , K. Kamardin , N. N. A. Sjarif , and N. Mohamed , “Offline Signature Verification Using Deep Learning Convolutional Neural Network (CNN) Architectures GoogLeNet Inception‐v1 and Inception‐v3,” Procedia Computer Science 161 (2019): 475–483.

[115] W. Xu , Y. L. Fu , and D. Zhu , “ResNet and Its Application to Medical Image Processing: Research Progress and Challenges,” Computer Methods and Programs in Biomedicine 240 (2023): 107660. 37320940 10.1016/j.cmpb.2023.107660

[116] L. Borawar and K. R. ResNet , Solving Vanishing Gradient in Deep Networks. In: *Proceedings of International Conference on Recent Trends in Computing: ICRTC 2022* . Springer; 2023:235–247.

[117] Z. Kurt , S. Isik , Z. Kaya , Y. Anagun , N. Koca , and S. Cicek , “Evaluation of EfficientNet Models for COVID‐19 Detection Using Lung Parenchyma,” Neural Computing and Applications 35, no. 16 (2023): 12121–12132. 36843903 10.1007/s00521-023-08344-z PMC9940669

[118] H. O. Ahmed and A. K. Nandi , “High Performance Breast Cancer Diagnosis From Mammograms Using Mixture of Experts With EfficientNet Features (MoEffNet),” IEEE Access 12 (2024): 133703–133725.

[119] Q. Abbas , Y. Daadaa , U. Rashid , M. Z. Sajid , and M. E. A. Ibrahim , “HDR‐EfficientNet: A Classification of Hypertensive and Diabetic Retinopathy Using Optimize EfficientNet Architecture,” Diagnostics (Basel) 13, no. 20 (2023): 3236. 37892058 10.3390/diagnostics13203236 PMC10606674

[120] N. Li , Y. Chen , W. Li , Z. Ding , D. Zhao , and N. S. BViT , “Broad Attention‐based Vision Transformer,” IEEE Transactions on Neural Networks and Learning Systems 35, no. 9 (2023): 12772–12783. 10.1109/TNNLS.2023.3264730 37126636

[121] J. Maurício , I. Domingues , and J. Bernardino , “Comparing Vision Transformers and Convolutional Neural Networks for Image Classification: A Literature Review,” Applied Sciences 13, no. 9 (2023): 5521.

[122] O. N. Manzari , H. Ahmadabadi , H. Kashiani , S. B. Shokouhi , and A. Ayatollahi , “MedViT: A Robust Vision Transformer for Generalized Medical Image Classification,” Computers in Biology and Medicine 157 (2023): 106791. 36958234 10.1016/j.compbiomed.2023.106791

[123] L. Zhang , X. Wang , D. Yang , et al., “Generalizing Deep Learning for Medical Image Segmentation to Unseen Domains via Deep Stacked Transformation,” Ieee Transactions on Medical Imaging 39, no. 7 (2020): 2531–2540. 32070947 10.1109/TMI.2020.2973595 PMC7393676

[124] F. J. Gilbert , L. Tucker , and K. C. Young , “Digital Breast Tomosynthesis (DBT): A Review of the Evidence for Use as a Screening Tool,” Clinical Radiology 71, no. 2 (2016): 141–150. 26707815 10.1016/j.crad.2015.11.008

[125] C. Mandoul , C. Verheyden , I. Millet , et al., “Breast Tomosynthesis: What Do We Know and Where Do We Stand?,” Diagn Interv Imaging 100, no. 10 (2019): 537–551. 31427217 10.1016/j.diii.2019.07.012

[126] P. S. Sujlana , M. Mahesh , S. Vedantham , S. C. Harvey , L. A. Mullen , and R. W. Woods , “Digital Breast Tomosynthesis: Image Acquisition Principles and Artifacts,” Clinical Imaging 55 (2019): 188–195. 30236642 10.1016/j.clinimag.2018.07.013

[127] C. I. Lee and C. D. Lehman , “Digital Breast Tomosynthesis and the Challenges of Implementing an Emerging Breast Cancer Screening Technology into Clinical Practice,” J Am Coll Radiol 13, no. 11S (2016): R61–R66. 27814817 10.1016/j.jacr.2016.09.029

[128] Y. Gao , L. Moy , and S. L. Heller , “Digital Breast Tomosynthesis: Update on Technology, Evidence, and Clinical Practice,” Radiographics 41, no. 2 (2021): 321–337. 33544665 10.1148/rg.2021200101 PMC8170874

[129] N. W. Marshall and H. Bosmans , “Performance Evaluation of Digital Breast Tomosynthesis Systems: Physical Methods and Experimental Data,” Physics in Medicine and Biology 67, no. 22 (2022): 22TR03. 10.1088/1361-6560/ac9a35 36228632

[130] R. K. Samala , H.‐P. Chan , L. Hadjiiski , M. A. Helvie , C. D. Richter , and K. H. Cha , “Breast Cancer Diagnosis in Digital Breast Tomosynthesis: Effects of Training Sample Size on Multi‐stage Transfer Learning Using Deep Neural Nets,” Ieee Transactions on Medical Imaging 38, no. 3 (2018): 686–696. 10.1109/TMI.2018.2870343 PMC6812655 31622238

[131] R. V. Aswiga and A. P. Shanthi , “A Multilevel Transfer Learning Technique and LSTM Framework for Generating Medical Captions for Limited CT and DBT Images,” Journal of Digital Imaging 35, no. 3 (2022): 564–580. 35217942 10.1007/s10278-021-00567-7 PMC9156604

[132] R. K. Samala , H. P. Chan , L. M. Hadjiiski , M. A. Helvie , C. Richter , and K. Cha , “Evolutionary Pruning of Transfer Learned Deep Convolutional Neural Network for Breast Cancer Diagnosis in Digital Breast Tomosynthesis,” Physics in Medicine and Biology 63, no. 9 (2018): 095005. 29616660 10.1088/1361-6560/aabb5b PMC5967610

[133] Y.‐D. Zhang , S. C. Satapathy , D. S. Guttery , J. M. Górriz , and S.‐H. Wang , “Improved Breast Cancer Classification Through Combining Graph Convolutional Network and Convolutional Neural Network,” Inf Process Manage 58, no. 2 (2021): 102439.

[134] S. D. Pawar , K. K. Sharma , S. G. Sapate , et al., “Multichannel DenseNet Architecture for Classification of Mammographic Breast Density for Breast Cancer Detection,” Front Public Health 10 (2022): 885212. 35548086 10.3389/fpubh.2022.885212 PMC9081505

[135] Z. Q. Habeeb , B. Vuksanovic , and I. Q. Al‐Zaydi , “Breast Cancer Detection Using Image Processing and Machine Learning,” J Image Graph (UK) 11, no. 1 (2023): 1–8.

[136] X. Chen , Y. Zhang , J. Zhou , et al., “Diagnosis of Architectural Distortion on Digital Breast Tomosynthesis Using Radiomics and Deep Learning,” Frontiers in oncology 12 (2022): 991892. 36582788 10.3389/fonc.2022.991892 PMC9792864

[137] D. Esposito , G. Paternò , R. Ricciardi , A. Sarno , P. Russo , and G. Mettivier , “A Pre‐processing Tool to Increase Performance of Deep Learning‐based CAD in Digital Breast Tomosynthesis,” Health Technology 14, no. 1 (2024): 81–91.

[138] K. Mendel , H. Li , D. Sheth , and M. Giger , “Transfer Learning from Convolutional Neural Networks for Computer‐Aided Diagnosis: A Comparison of Digital Breast Tomosynthesis and Full‐Field Digital Mammography,” Academic Radiology 26, no. 6 (2019): 735–743. 30076083 10.1016/j.acra.2018.06.019 PMC6355376

[139] A. A. Mukhlif , B. Al‐Khateeb , and M. A. Mohammed , “Incorporating a Novel Dual Transfer Learning Approach for Medical Images,” Sensors (Basel) 23, no. 2 (2023): 570. 36679370 10.3390/s23020570 PMC9866662

[140] N. A. Harron , S. N. Sulaiman , M. K. Osman , I. S. Isa , N. K. A. Karim , and M. I. F. Maruzuki , “Deep Learning Approach for Blur Detection of Digital Breast Tomosynthesis Images,” Journal of Electrical & Electronic Systems Research 21 (2022): 39–44.

[141] Z. Cao , L. Duan , G. Yang , T. Yue , and Q. Chen , “An Experimental Study on Breast Lesion Detection and Classification From Ultrasound Images Using Deep Learning Architectures,” BMC Medical Imaging 19, no. 1 (2019): 51. 31262255 10.1186/s12880-019-0349-x PMC6604293

[142] M. A. Kassem , K. M. Hosny , R. Damasevicius , and M. M. Eltoukhy , “Machine Learning and Deep Learning Methods for Skin Lesion Classification and Diagnosis: A Systematic Review,” Diagnostics (Basel) 11, no. 8 (2021): 1390. 34441324 10.3390/diagnostics11081390 PMC8391467

[143] H. Jiang , Z. Diao , T. Shi , et al., “A Review of Deep Learning‐based Multiple‐lesion Recognition From Medical Images: Classification, Detection and Segmentation,” Computers in Biology and Medicine 157 (2023): 106726. 36924732 10.1016/j.compbiomed.2023.106726

[144] M. Ahammed , M. Al Mamun , and M. S. Uddin , “A Machine Learning Approach for Skin Disease Detection and Classification Using Image Segmentation,” Healthcare Analytics 2 (2022): 100122.

[145] A. Krizhevsky , I. Sutskever , and G. E. Hinton , “Imagenet Classification With Deep Convolutional Neural networks,” Advances in Neural Information Processing Systems. (Curran Associates, Inc, 2012): 1–9.

[146] S. S. Islam , S. Rahman , M. M. Rahman , E. K. Dey , and M. Shoyaib , “Application of Deep Learning to Computer Vision: A Comprehensive Study,” In: 2016 5th international conference on informatics, electronics and vision (ICIEV) . IEEE; 2016:592–597.

[147] X. Chen , C. Lian , H. H. Deng , et al., “Fast and Accurate Craniomaxillofacial Landmark Detection via 3D Faster R‐CNN,” Ieee Transactions on Medical Imaging 40, no. 12 (2021): 3867–3878. 34310293 10.1109/TMI.2021.3099509 PMC8686670

[148] S. Ren , K. He , R. Girshick , and J. Sun , “Faster R‐CNN: Towards Real‐Time Object Detection With Region Proposal Networks,” Ieee Transactions on Pattern Analysis and Machine Intelligence 39, no. 6 (2017): 1137–1149. 27295650 10.1109/TPAMI.2016.2577031

[149] A. Rasheed , S. H. Shirazi , A. I. Umar , M. Shahzad , W. Yousaf , and Z. Khan , “Cervical Cell's Nucleus Segmentation Through an Improved UNet Architecture,” PLoS ONE 18, no. 10 (2023): e0283568. 37788295 10.1371/journal.pone.0283568 PMC10547184

[150] Y. Su , Q. Liu , W. Xie , and P. Hu , “YOLO‐LOGO: A Transformer‐based YOLO Segmentation Model for Breast Mass Detection and Segmentation in Digital Mammograms,” Computer Methods and Programs in Biomedicine 221 (2022): 106903. 35636358 10.1016/j.cmpb.2022.106903

[151] Y. D. Jeon , M. J. Kang , S. U. Kuh , et al., “Deep Learning Model Based on You Only Look Once Algorithm for Detection and Visualization of Fracture Areas in Three‐Dimensional Skeletal Images,” Diagnostics (Basel) 14, no. 1 (2023): 11. 38201320 10.3390/diagnostics14010011 PMC10802847

[152] M. Durve , S. Orsini , A. Tiribocchi , et al., “Benchmarking YOLOv5 and YOLOv7 Models With DeepSORT for Droplet Tracking Applications,” The European Physical Journal. E, Soft Matter 46, no. 5 (2023): 32. 37154834 10.1140/epje/s10189-023-00290-x PMC10167152

[153] R. Azad , E. K. Aghdam , A. Rauland , et al., “Medical Image Segmentation Review: The Success of U‐Net,” Ieee Transactions on Pattern Analysis and Machine Intelligence 46, no. 12 (2024): 10076–10095. 39167505 10.1109/TPAMI.2024.3435571

[154] J. Cheng , W. Xiong , W. Chen , Y. Gu , and Y. Li , “Pixel‐level Crack Detection Using U‐Net,” In: TENCON 2018‐2018 IEEE region 10 conference . IEEE; 2018:0462–0466.

[155] M. Agarwal , S. K. Gupta , and K. K. Biswas , “Development of a Compressed FCN Architecture for Semantic Segmentation Using Particle Swarm Optimization,” Neural Computing and Applications 35, no. 16 (2023): 11833–11846. 36778195 10.1007/s00521-023-08324-3 PMC9897161

[156] E. Evain , C. Raynaud , C. Ciofolo‐Veit , et al., “Breast Nodule Classification With Two‐dimensional Ultrasound Using Mask‐RCNN Ensemble Aggregation,” Diagnostic and Interventional Imaging 102, no. 11 (2021): 653–658. 34600861 10.1016/j.diii.2021.09.002

[157] L. C. Chen , G. Papandreou , I. Kokkinos , K. Murphy , and A. L. Yuille , “DeepLab: Semantic Image Segmentation With Deep Convolutional Nets, Atrous Convolution, and Fully Connected CRFs,” Ieee Transactions on Pattern Analysis and Machine Intelligence 40, no. 4 (2018): 834–848. 28463186 10.1109/TPAMI.2017.2699184

[158] K. Zhou , W. Li , and D. Zhao , “Deep Learning‐based Breast Region Extraction of Mammographic Images Combining Pre‐processing Methods and Semantic Segmentation Supported by Deeplab v3,” Technology and Health Care 30, no. S1 (2022): 173–190. 35124595 10.3233/THC-228017 PMC9028646

[159] B. Felfeliyan , A. Hareendranathan , G. Kuntze , J. L. Jaremko , and J. L. Ronsky , “Improved‐Mask R‐CNN: Towards an Accurate Generic MSK MRI Instance Segmentation Platform (data From the Osteoarthritis Initiative),” Computerized Medical Imaging and Graphics 97 (2022): 102056. 35364383 10.1016/j.compmedimag.2022.102056

[160] F. Shamshad , S. Khan , S. W. Zamir , et al., “Transformers in Medical Imaging: A Survey,” Medical Image Analysis 88 (2023): 102802. 37315483 10.1016/j.media.2023.102802

[161] K. He , C. Gan , Z. Li , et al., “Transformers in Medical Image Analysis,” Intelligent Medicine 3, no. 1 (2023): 59–78.

[162] K. Xia and J. Wang , “Recent Advances of Transformers in Medical Image Analysis: A Comprehensive Review,” MedComm 2, no. 1 (2023): e38.

[163] K. Han , Y. Wang , H. Chen , et al., “A Survey on Vision Transformer,” Ieee Transactions on Pattern Analysis and Machine Intelligence 45, no. 1 (2023): 87–110. 35180075 10.1109/TPAMI.2022.3152247

[164] S. Yan , C. Wang , W. Chen , and J. Lyu , “Swin Transformer‐based GAN for Multi‐modal Medical Image Translation,” Frontiers in oncology 12 (2022): 942511. 36003791 10.3389/fonc.2022.942511 PMC9395186

[165] S. V. Fotin , Y. Yin , H. Haldankar , J. W. Hoffmeister , and S. Periaswamy , “Detection of Soft Tissue Densities From Digital Breast Tomosynthesis: Comparison of Conventional and Deep Learning approaches,” Medical Imaging 2016: Computer‐aided Diagnosis. (SPIE, 2016): 228–233.

[166] N. Konz , M. Buda , H. Gu , et al., “A Competition, Benchmark, Code, and Data for Using Artificial Intelligence to Detect Lesions in Digital Breast Tomosynthesis,” JAMA Network Open 6, no. 2 (2023): e230524. 36821110 10.1001/jamanetworkopen.2023.0524 PMC9951043

[167] M. Buda , A. Saha , R. Walsh , et al., “A Data Set and Deep Learning Algorithm for the Detection of Masses and Architectural Distortions in Digital Breast Tomosynthesis Images,” JAMA Network Open 4, no. 8 (2021): e2119100. 34398205 10.1001/jamanetworkopen.2021.19100 PMC8369362

[168] R. Aggarwal , V. Sounderajah , G. Martin , et al., “Diagnostic Accuracy of Deep Learning in Medical Imaging: A Systematic Review and Meta‐analysis,” NPJ Digital Medicine 4, no. 1 (2021): 65. 33828217 10.1038/s41746-021-00438-z PMC8027892

[169] A. J. Maxwell , M. Michell , Y. Y. Lim , et al., “A Randomised Trial of Screening With Digital Breast Tomosynthesis plus Conventional Digital 2D Mammography versus 2D Mammography Alone in Younger Higher Risk Women,” European Journal of Radiology 94 (2017): 133–139. 28716454 10.1016/j.ejrad.2017.06.018

[170] K. Simon , K. Dodelzon , M. Drotman , et al., “Accuracy of Synthetic 2D Mammography Compared with Conventional 2D Digital Mammography Obtained with 3D Tomosynthesis,” Ajr American Journal of Roentgenology 212, no. 6 (2019): 1406–1411. 30917028 10.2214/AJR.18.20520

[171] S. Kulkarni , V. Freitas , and D. Muradali , “Digital Breast Tomosynthesis: Potential Benefits in Routine Clinical Practice,” Canadian Association of Radiologists Journal 73, no. 1 (2022): 107–120. 34229477 10.1177/08465371211025229

[172] D. B. Russakoff , T. Rohlfing , K. Mori , et al., “Fast Generation of Digitally Reconstructed Radiographs Using Attenuation Fields With Application to 2D‐3D Image Registration,” Ieee Transactions on Medical Imaging 24, no. 11 (2005): 1441–1454. 16279081 10.1109/TMI.2005.856749

[173] Y. Gao and L. Moy , “Phase‐Sensitive Breast Tomosynthesis May Address Shortcomings of Digital Breast Tomosynthesis,” Radiology 306, no. 2 (2022): e222184. 36165798 10.1148/radiol.222184 PMC9885346

[174] I. Kassis , D. Lederman , G. Ben‐Arie , M. Giladi Rosenthal , I. Shelef , and Y. Zigel , “Detection of Breast Cancer in Digital Breast Tomosynthesis With Vision Transformers,” Scientific Reports 14, no. 1 (2024): 22149. 39333178 10.1038/s41598-024-72707-2 PMC11436893

[175] S. M. McKinney , M. Sieniek , V. Godbole , et al., “International Evaluation of an AI System for Breast Cancer Screening,” Nature 577, no. 7788 (2020): 89–94. 31894144 10.1038/s41586-019-1799-6

[176] X. Lai , W. Yang , and R. Li , “DBT Masses Automatic Segmentation Using U‐Net Neural Networks,” Computational and Mathematical Methods in Medicine 2020, no. 1 (2020): 7156165. 32411285 10.1155/2020/7156165 PMC7204342

[177] M. Fan , Y. Li , S. Zheng , W. Peng , W. Tang , and L. Li , “Computer‐aided Detection of Mass in Digital Breast Tomosynthesis Using a Faster Region‐based Convolutional Neural Network,” Methods (San Diego, Calif.) 166 (2019): 103–111. 30771490 10.1016/j.ymeth.2019.02.010

[178] M. Fan , H. Zheng , S. Zheng , et al., “Mass Detection and Segmentation in Digital Breast Tomosynthesis Using 3D‐Mask Region‐Based Convolutional Neural Network: A Comparative Analysis,” Frontiers in Molecular Biosciences 7 (2020): 599333. 33263004 10.3389/fmolb.2020.599333 PMC7686533

[179] M. B. Hossain , R. M. Nishikawa , and J. Lee , “Developing Breast Lesion Detection Algorithms for Digital Breast Tomosynthesis: Leveraging False Positive Findings,” Medical Physics 49, no. 12 (2022): 7596–7608. 35916103 10.1002/mp.15883 PMC10156088

[180] J. Sun , X. Wang , N. Xiong , and J. Shao , “Learning Sparse Representation With Variational Auto‐encoder for Anomaly Detection,” IEEE Access 6 (2018): 33353–33361.

[181] R. F. Mansour , J. Escorcia‐Gutierrez , M. Gamarra , D. Gupta , O. Castillo , and S. Kumar , “Unsupervised Deep Learning Based Variational Autoencoder Model for COVID‐19 Diagnosis and Classification,” Pattern Recognition Letters 151 (2021): 267–274. 34566223 10.1016/j.patrec.2021.08.018 PMC8455283

[182] H. Uzunova , S. Schultz , H. Handels , and J. Ehrhardt , “Unsupervised Pathology Detection in Medical Images Using Conditional Variational Autoencoders,” International Journal of Computer Assisted Radiology and Surgery 14, no. 3 (2019): 451–461. 30542975 10.1007/s11548-018-1898-0

[183] I. Goodfellow , J. Pouget‐Abadie , M. Mirza , et al., “Generative Adversarial Networks,” Communications of the Acm 63, no. 11 (2020): 139–144.

[184] N. K. Singh and K. Raza . Medical Image Generation Using Generative Adversarial Networks: A review. In: R. Patgiri , A. Biswas , P. Roy , eds. “Health Informatics: A Computational Perspective in Healthcare” (Springer: Singapore, 2021): 77–96.

[185] D. Hu , L. Wang , W. Jiang , S. Zheng , and B. Li , “A Novel Image Steganography Method via Deep Convolutional Generative Adversarial Networks,” IEEE Access 6 (2018): 38303–38314.

[186] R. Toda , A. Teramoto , M. Kondo , K. Imaizumi , K. Saito , and H. Fujita , “Lung Cancer CT Image Generation From a Free‐form Sketch Using Style‐based pix2pix for Data Augmentation,” Scientific Reports 12, no. 1 (2022): 12867. 35896575 10.1038/s41598-022-16861-5 PMC9329467

[187] Y. Zhang , S. Liu , C. Dong , X. Zhang , and Y. Yuan , “Multiple Cycle‐in‐Cycle Generative Adversarial Networks for Unsupervised Image Super‐Resolution,” Ieee Transactions on Image Processing 29 (2019): 1101–1112. 10.1109/TIP.2019.2938347 31502972

[188] Q. Yang , P. Yan , Y. Zhang , et al., “Low‐Dose CT Image Denoising Using a Generative Adversarial Network with Wasserstein Distance and Perceptual Loss,” Ieee Transactions on Medical Imaging 37, no. 6 (2018): 1348–1357. 29870364 10.1109/TMI.2018.2827462 PMC6021013

[189] H. Ma , D. Liu , and F. Wu , “Rectified wasserstein Generative Adversarial Networks for Perceptual Image Restoration,” Ieee Transactions on Pattern Analysis and Machine Intelligence 45, no. 3 (2022): 3648–3663. 10.1109/TPAMI.2022.3185316 35731773

[190] Z. Ni , W. Yang , S. Wang , L. Ma , and S. Kwong , “Towards Unsupervised Deep Image Enhancement With Generative Adversarial Network,” Ieee Transactions on Image Processing 29 (2020): 9140–9151. 10.1109/TIP.2020.3023615 32960763

[191] V. Bharti , B. Biswas , and K. K. Shukla , “EMOCGAN: A Novel Evolutionary Multiobjective Cyclic Generative Adversarial Network and Its Application to Unpaired Image Translation,” Neural Computing and Applications 34, no. 24 (2022): 21433–21447.

[192] P. Hambar , Z. Gosher , S. Fengade , J. Jain , R. Nikam , and S. Dange , “Contrastive Learning Approach for Text‐to Image Synthesis,” In: 2023 International Conference on Advanced Computing Technologies and Applications (ICACTA) . IEEE; 2023:1–7.

[193] C. Zheng , T.‐L. Vuong , J. Cai , and D. Phung , “Movq: Modulating Quantized Vectors for High‐fidelity Image Generation,” Advances in Neural Information Processing Systems 35 (2022): 23412–23425.

[194] A. Gallucci , D. Znamenskiy , Y. Long , N. Pezzotti , and M. Petkovic , “Generating High‐Resolution 3D Faces and Bodies Using VQ‐VAE‐2 With PixelSNAIL Networks on 2D Representations,” Sensors (Basel) 23, no. 3 (2023): 1168. 36772208 10.3390/s23031168 PMC9921729

[195] I. Gligorea , M. Cioca , R. Oancea , A.‐T. Gorski , H. Gorski , and P. Tudorache , “Adaptive Learning Using Artificial Intelligence in E‐learning: A Literature Review,” Education Sciences 13, no. 12 (2023): 1216.

[196] W. Lin , Z. Zhao , X. Zhang , et al., “Pmc‐clip: Contrastive Language‐image Pre‐training Using Biomedical Documents,” In: International Conference on Medical Image Computing and Computer‐Assisted Intervention . Springer; 2023:525–536.

[197] J. Liu , H.‐Y. Zhou , C. Li , et al., “Mlip: Medical Language‐image Pre‐training With Masked Local Representation Learning,” In: 2024 IEEE International Symposium on Biomedical Imaging (ISBI) . IEEE; 2024:1–5.

[198] S. W. Zamir , A. Arora , S. Khan , et al., “Learning Enriched Features for Real Image Restoration and Enhancement,” In: Computer Vision–ECCV2020: 16th European Conference, Glasgow, UK, August 23–28, 2020, Proceedings, Part XXV 16 . Springer; 2020:492–511.

[199] L. Chen , P. Bentley , K. Mori , K. Misawa , M. Fujiwara , and D. Rueckert , “Self‐supervised Learning for Medical Image Analysis Using Image Context Restoration,” Medical Image Analysis 58 (2019): 101539. 31374449 10.1016/j.media.2019.101539 PMC7613987

[200] X. Liu , L. Song , S. Liu , and Y. Zhang , “A Review of Deep‐learning‐based Medical Image Segmentation Methods,” Sustainability 13, no. 3 (2021): 1224.

[201] L. Cai , J. Gao , and D. Zhao , “A Review of the Application of Deep Learning in Medical Image Classification and Segmentation,” Annals of translational medicine 8, no. 11 (2020): 713. 32617333 10.21037/atm.2020.02.44 PMC7327346

[202] D. Nie , R. Trullo , J. Lian , et al., “Medical Image Synthesis With Deep Convolutional Adversarial Networks,” Ieee Transactions on Bio‐Medical Engineering 65, no. 12 (2018): 2720–2730. 29993445 10.1109/TBME.2018.2814538 PMC6398343

[203] D. Nie , R. Trullo , J. Lian , et al., “Medical Image Synthesis With Context‐aware Generative Adversarial Networks,” In: Medical Image Computing and Computer Assisted Intervention− MICCAI 2017: 20th International Conference, Quebec City, QC, Canada, September 11–13, 2017, Proceedings, Part III 20 . Springer; 2017:417–425. 10.1007/978-3-319-66179-7_48 PMC6044459 30009283

[204] X. Li , L. Yu , H. Chen , C.‐W. Fu , L. Xing , and P.‐A. Heng , “Transformation‐consistent Self‐ensembling Model for Semisupervised Medical Image Segmentation,” IEEE Transactions on Neural Networks and Learning Systems 32, no. 2 (2020): 523–534. 10.1109/TNNLS.2020.2995319 32479407

[205] T. Fernando , H. Gammulle , S. Denman , S. Sridharan , and C. Fookes , “Deep Learning for Medical Anomaly Detection–a Survey,” ACM Computing Surveys (CSUR) 54, no. 7 (2021): 1–37.

[206] E. Shivhare and V. Saxena , “Optimized Generative Adversarial Network Based Breast Cancer Diagnosis With Wavelet and Texture Features,” Multimedia Systems 28, no. 5 (2022): 1639–1655.

[207] E. Strelcenia and S. Prakoonwit , “Improving Cancer Detection Classification Performance Using GANs in Breast Cancer Data,” IEEE Access 11 (2023): 71594–71615.

[208] S. Guan and M. Loew , “Breast Cancer Detection Using Synthetic Mammograms From Generative Adversarial Networks in Convolutional Neural Networks,” Journal of Medical Imaging (Bellingham) 6, no. 3 (2019): 031411. 10.1117/1.JMI.6.3.031411 PMC6430964 30915386

[209] M. Gao , J. A. Fessler , and H. P. Chan , “Deep Convolutional Neural Network with Adversarial Training for Denoising Digital Breast Tomosynthesis Images,” Ieee Transactions on Medical Imaging 40, no. 7 (2021): 1805–1816. 33729933 10.1109/TMI.2021.3066896 PMC8274391

[210] A. Swiecicki , N. Konz , M. Buda , and M. A. Mazurowski , “A Generative Adversarial Network‐based Abnormality Detection Using Only Normal Images for Model Training With Application to Digital Breast Tomosynthesis,” Scientific Reports 11, no. 1 (2021): 10276. 33986361 10.1038/s41598-021-89626-1 PMC8119417

[211] D. Shah , M. A. Ullah Khan , and M. Abrar , “Reliable Breast Cancer Diagnosis With Deep Learning: DCGAN‐Driven Mammogram Synthesis and Validity Assessment,” Applied Computational Intelligence and Soft Computing 2024, no. 1 (2024): 1122109.

[212] F. Shahidi , “Breast Cancer Histopathology Image Super‐resolution Using Wide‐attention Gan With Improved Wasserstein Gradient Penalty and Perceptual Loss,” IEEE Access 9 (2021): 32795–32809.

[213] J. Lee and R. M. Nishikawa , “Identifying Women with Mammographically‐ Occult Breast Cancer Leveraging GAN‐Simulated Mammograms,” Ieee Transactions on Medical Imaging 41, no. 1 (2022): 225–236. 34460371 10.1109/TMI.2021.3108949 PMC8799372

[214] M. Staffa , L. D'Errico , R. Ricciardi , et al., “How to Increase and Balance Current DBT Datasets via an Evolutionary GAN: Preliminary Results,” In: 2022 22nd IEEE International Symposium on Cluster, Cloud and Internet Computing (CCGrid) . IEEE; 2022:913–920.

[215] J. Son , S. E. Lee , E. K. Kim , and S. Kim , “Prediction of Breast Cancer Molecular Subtypes Using Radiomics Signatures of Synthetic Mammography From Digital Breast Tomosynthesis,” Scientific Reports 10, no. 1 (2020): 21566. 33299040 10.1038/s41598-020-78681-9 PMC7726048

[216] M. Ma , R. Liu , C. Wen , et al., “Predicting the Molecular Subtype of Breast Cancer and Identifying Interpretable Imaging Features Using Machine Learning Algorithms,” European Radiology 32, no. 3 (2022): 1652–1662. 34647174 10.1007/s00330-021-08271-4

[217] J. Gu , T. Tong , C. He , et al., “Deep Learning Radiomics of Ultrasonography Can Predict Response to Neoadjuvant Chemotherapy in Breast Cancer at an Early Stage of Treatment: A Prospective Study,” European Radiology 32, no. 3 (2022): 2099–2109. 34654965 10.1007/s00330-021-08293-y

[218] D. Zuo , L. Yang , Y. Jin , H. Qi , Y. Liu , and L. Ren , “Machine Learning‐based Models for the Prediction of Breast Cancer Recurrence Risk,” BMC Medical Informatics and Decision Making [Electronic Resource] 23, no. 1 (2023): 276. 38031071 10.1186/s12911-023-02377-z PMC10688055

[219] D. Shimokawa , K. Takahashi , K. Oba , et al., “Deep Learning Model for Predicting the Presence of Stromal Invasion of Breast Cancer on Digital Breast Tomosynthesis,” Radiological Physics and Technology 16, no. 3 (2023): 406–413. 37466807 10.1007/s12194-023-00731-4

[220] M. M. Schmitgen , I. Niedtfeld , R. Schmitt , et al., “Individualized Treatment Response Prediction of Dialectical Behavior Therapy for Borderline Personality Disorder Using Multimodal Magnetic Resonance Imaging,” Brain and Behavior 9, no. 9 (2019): e01384. 31414575 10.1002/brb3.1384 PMC6749487

[221] B. Rigaud , O. O. Weaver , J. B. Dennison , et al., “Deep Learning Models for Automated Assessment of Breast Density Using Multiple Mammographic Image Types,” Cancers (Basel) 14, no. 20 (2022): 5003. 36291787 10.3390/cancers14205003 PMC9599904

[222] K. Michielsen , A. Rodriguez‐Ruiz , I. Reiser , J. G. Nagy , and I. Sechopoulos , “Iodine Quantification in Limited Angle Tomography,” Medical Physics 47, no. 10 (2020): 4906–4916. 32803800 10.1002/mp.14400 PMC7689880

[223] H. Jang and J. Baek , “Convolutional Neural Network‐based Model Observer for Signal Known Statistically Task in Breast Tomosynthesis Images,” Medical Physics 50, no. 10 (2023): 6390–6408. 36971505 10.1002/mp.16395

[224] M. Gao , J. A. Fessler , and H. P. Chan , “Model‐based Deep CNN‐regularized Reconstruction for Digital Breast Tomosynthesis With a Task‐based CNN Image Assessment Approach,” Physics in Medicine and Biology 68, no. 24 (2023): 245024. 10.1088/1361-6560/ad0eb4 PMC10719554 37988758

[225] T. Su , X. Deng , J. Yang , et al., “DIR‐DBTnet: Deep Iterative Reconstruction Network for Three‐dimensional Digital Breast Tomosynthesis Imaging,” Medical Physics 48, no. 5 (2021): 2289–2300. 33594671 10.1002/mp.14779

[226] B. Yang , Y. Wu , Z. Zhou , et al., “A Collection Input Based Support Tensor Machine for Lesion Malignancy Classification in Digital Breast Tomosynthesis,” Physics in Medicine and Biology 64, no. 23 (2019): 235007. 31698349 10.1088/1361-6560/ab553d PMC7103089

[227] J. Wang , H. Sun , K. Jiang , et al., “CAPNet: Context Attention Pyramid Network for Computer‐aided Detection of Microcalcification Clusters in Digital Breast Tomosynthesis,” Computer Methods and Programs in Biomedicine 242 (2023): 107831. 37783114 10.1016/j.cmpb.2023.107831

[228] R. K. Samala , H. P. Chan , L. Hadjiiski , M. A. Helvie , J. Wei , and K. Cha , “Mass Detection in Digital Breast Tomosynthesis: Deep Convolutional Neural Network With Transfer Learning From Mammography,” Medical Physics 43, no. 12 (2016): 6654–6666. 27908154 10.1118/1.4967345 PMC5135717

[229] A. M. Mota , M. J. Clarkson , P. Almeida , and N. Matela , “Automatic Classification of Simulated Breast Tomosynthesis Whole Images for the Presence of Microcalcification Clusters Using Deep CNNs,” Journal of Imaging 8, no. 9 (2022): 231. 36135397 10.3390/jimaging8090231 PMC9503015

[230] E. F. Conant , A. Y. Toledano , S. Periaswamy , et al., “Improving Accuracy and Efficiency With Concurrent Use of Artificial Intelligence for Digital Breast Tomosynthesis,” Radiology: Artificial Intelligence 1, no. 4 (2019): e180096. 32076660 10.1148/ryai.2019180096 PMC6677281

[231] B. Xiao , H. Sun , Y. Meng , et al., “Classification of Microcalcification Clusters in Digital Breast Tomosynthesis Using Ensemble Convolutional Neural Network,” Biomedical Engineering Online [Electronic Resource] 20, no. 1 (2021): 71. 34320986 10.1186/s12938-021-00908-1 PMC8317331

[232] H. M. Whitney , H. Li , Y. Ji , P. Liu , and M. L. Giger , “Comparison of Breast MRI Tumor Classification Using Human‐Engineered Radiomics, Transfer Learning from Deep Convolutional Neural Networks, and Fusion Methods,” Proceedings of the IEEE 108, no. 1 (2020): 163–177. 34045769 10.1109/jproc.2019.2950187 PMC8152568

[233] T. P. Matthews , S. Singh , B. Mombourquette , et al., “A Multisite Study of a Breast Density Deep Learning Model for Full‐Field Digital Mammography and Synthetic Mammography,” Radiology: Artificial Intelligence 3, no. 1 (2021): e200015. 33937850 10.1148/ryai.2020200015 PMC8082294

[234] D. H. Kim , S. T. Kim , J. M. Chang , and Y. M. Ro , “Latent Feature Representation With Depth Directional Long‐term Recurrent Learning for Breast Masses in Digital Breast Tomosynthesis,” Physics in Medicine and Biology 62, no. 3 (2017): 1009–1031. 28081006 10.1088/1361-6560/aa504e

[235] M. L. Altoe , A. Marone , H. K. Kim , et al., “Diffuse Optical Tomography of the Breast: A Potential Modifiable Biomarker of Breast Cancer Risk With Neoadjuvant Chemotherapy,” Biomedical Optics Express 10, no. 8 (2019): 4305–4315. 31453012 10.1364/BOE.10.004305 PMC6701514

[236] A. S. Tagliafico , B. Bignotti , F. Rossi , et al., “Breast Cancer Ki‐67 Expression Prediction by Digital Breast Tomosynthesis Radiomics Features,” European Radiology Experimental 3, no. 1 (2019): 36. 31414273 10.1186/s41747-019-0117-2 PMC6694353

[237] M. G. Davey , M. S. Davey , M. R. Boland , E. J. Ryan , A. J. Lowery , and M. J. Kerin , “Radiomic Differentiation of Breast Cancer Molecular Subtypes Using Pre‐operative Breast Imaging—A Systematic Review and Meta‐analysis,” European Journal of Radiology 144 (2021): 109996. 34624649 10.1016/j.ejrad.2021.109996

[238] E. K. Park , K. S. Lee , B. K. Seo , et al., “Machine Learning Approaches to Radiogenomics of Breast Cancer Using Low‐Dose Perfusion Computed Tomography: Predicting Prognostic Biomarkers and Molecular Subtypes,” Scientific Reports 9, no. 1 (2019): 17847. 31780739 10.1038/s41598-019-54371-z PMC6882909

[239] I. Nissar , S. Alam , S. Masood , and M. Kashif , “MOB‐CBAM: A Dual‐channel Attention‐based Deep Learning Generalizable Model for Breast Cancer Molecular Subtypes Prediction Using Mammograms,” Computer Methods and Programs in Biomedicine 248 (2024): 108121. 38531147 10.1016/j.cmpb.2024.108121

[240] S. Cai , M. Yao , D. Cai , et al., “Association Between Digital Breast Tomosynthesis and Molecular Subtypes of Breast Cancer,” Oncology letters 17, no. 3 (2019): 2669–2676. 30867729 10.3892/ol.2019.9918 PMC6366033

[241] S. Huang , J. Yang , S. Fong , and Q. Zhao , “Artificial Intelligence in Cancer Diagnosis and Prognosis: Opportunities and Challenges,” Cancer Letters 471 (2020): 61–71. 31830558 10.1016/j.canlet.2019.12.007

[242] E. F. Conant , S. P. Zuckerman , E. S. McDonald , et al., “Five Consecutive Years of Screening With Digital Breast Tomosynthesis: Outcomes by Screening Year and Round,” Radiology 295, no. 2 (2020): 285–293. 32154771 10.1148/radiol.2020191751 PMC7193918

[243] G. Chugh , S. Kumar , and N. Singh , “Survey on Machine Learning and Deep Learning Applications in Breast Cancer Diagnosis,” Cognitive Computation 13, no. 6 (2021): 1451–1470.

[244] R. Ha , P. Chang , S. Mutasa , et al., “Convolutional Neural Network Using a Breast MRI Tumor Dataset Can Predict Oncotype Dx Recurrence Score,” Journal of Magnetic Resonance Imaging 49, no. 2 (2019): 518–524. 30129697 10.1002/jmri.26244 PMC8139130

[245] M. A. Durand , B. M. Haas , X. Yao , et al., “Early Clinical Experience With Digital Breast Tomosynthesis for Screening Mammography,” Radiology 274, no. 1 (2015): 85–92. 25188431 10.1148/radiol.14131319

[246] N. Alsheik , L. Blount , Q. Qiong , et al., “Outcomes by Race in Breast Cancer Screening with Digital Breast Tomosynthesis versus Digital Mammography,” Journal of the American College of Radiology 18, no. 7 (2021): 906–918. 33607065 10.1016/j.jacr.2020.12.033 PMC9391198

[247] E. F. Conant , W. E. Barlow , S. D. Herschorn , et al., “Association of Digital Breast Tomosynthesis vs Digital Mammography with Cancer Detection and Recall Rates by Age and Breast Density,” JAMA oncology 5, no. 5 (2019): 635–642. 30816931 10.1001/jamaoncol.2018.7078 PMC6512257

[248] M. Eriksson , S. Destounis , K. Czene , et al., “A Risk Model for Digital Breast Tomosynthesis to Predict Breast Cancer and Guide Clinical Care,” Science Translational Medicine 14, no. 644 (2022): eabn3971. 35544593 10.1126/scitranslmed.abn3971

[249] K. Johnson , K. Lang , D. M. Ikeda , A. Akesson , I. Andersson , and S. Zackrisson , “Interval Breast Cancer Rates and Tumor Characteristics in the Prospective Population‐based Malmo Breast Tomosynthesis Screening Trial,” Radiology 299, no. 3 (2021): 559–567. 33825509 10.1148/radiol.2021204106

[250] S. Niu , T. Yu , Y. Cao , Y. Dong , Y. Luo , and X. Jiang , “Digital Breast Tomosynthesis‐based Peritumoral Radiomics Approaches in the Differentiation of Benign and Malignant Breast Lesions,” Diagnostic and Interventional Radiology 28, no. 3 (2022): 217–225. 35748203 10.5152/dir.2022.20664 PMC9634934

[251] D. Wang , M. Liu , Z. Zhuang , et al., “Radiomics Analysis on Digital Breast Tomosynthesis: Preoperative Evaluation of Lymphovascular Invasion Status in Invasive Breast Cancer,” Academic Radiology 29, no. 12 (2022): 1773–1782. 35400556 10.1016/j.acra.2022.03.011

[252] M. Bahl , S. Mercaldo , P. A. Dang , A. M. McCarthy , K. P. Lowry , and C. D. Lehman , “Breast Cancer Screening With Digital Breast Tomosynthesis: Are Initial Benefits Sustained?,” Radiology 295, no. 3 (2020): 529–539. 32255414 10.1148/radiol.2020191030

[253] G. Kim , S. Mercaldo , and M. Bahl , “Impact of Digital Breast Tomosynthesis (DBT) on Finding Types Leading to True‐positive and False‐positive Examinations,” Clinical Imaging 71 (2021): 155–159. 33276203 10.1016/j.clinimag.2020.10.046

[254] Y. Peng , S. Wu , G. Yuan , et al., “A Radiomics Method to Classify Microcalcification Clusters in Digital Breast Tomosynthesis,” Medical Physics 47, no. 8 (2020): 3435–3446. 32358973 10.1002/mp.14216

[255] A. Sakai , Y. Onishi , M. Matsui , et al., “A Method for the Automated Classification of Benign and Malignant Masses on Digital Breast Tomosynthesis Images Using Machine Learning and Radiomic Features,” Radiological Physics and Technology 13, no. 1 (2020): 27–36. 31686300 10.1007/s12194-019-00543-5

[256] M. Wels , B. M. Kelm , M. Hammon , A. Jerebko , M. Sühling , and D. Comaniciu , “Data‐driven Breast Decompression and Lesion Mapping From Digital Breast Tomosynthesis,” In: Medical Image Computing and Computer‐Assisted Intervention–MICCAI 2012: 15th International Conference, Nice, France, October 1–5, 2012, Proceedings, Part I 15 . Springer; 2012:438–446. 10.1007/978-3-642-33415-3_54 23285581

[257] M. Chen , S. J. Copley , P. Viola , H. Lu , and E. O. Aboagye , “Radiomics and Artificial Intelligence for Precision Medicine in Lung Cancer treatment,” Semin Cancer Biol. (Elsevier, 2023): 97–113. 10.1016/j.semcancer.2023.05.004 37211292

[258] M. Avanzo , L. Wei , J. Stancanello , et al., “Machine and Deep Learning Methods for Radiomics,” Medical Physics 47, no. 5 (2020): e185–e202. 32418336 10.1002/mp.13678 PMC8965689

[259] S. Rizzo , F. Botta , S. Raimondi , et al., “Radiomics: The Facts and the Challenges of Image Analysis,” European Radiology Experimental 2, no. 1 (2018): 36. 30426318 10.1186/s41747-018-0068-z PMC6234198

[260] C. Scapicchio , M. Gabelloni , A. Barucci , D. Cioni , L. Saba , and E. Neri , “A Deep Look Into Radiomics,” La Radiologia Medica 126, no. 10 (2021): 1296–1311. 34213702 10.1007/s11547-021-01389-x PMC8520512

[261] F. Murtas , V. Landoni , P. Ordonez , et al., “Clinical‐radiomic Models Based on Digital Breast Tomosynthesis Images: A Preliminary Investigation of a Predictive Tool for Cancer Diagnosis,” Frontiers in oncology 13 (2023): 1152158. 37251915 10.3389/fonc.2023.1152158 PMC10213670

[262] A. S. Tagliafico , F. Valdora , G. Mariscotti , et al., “An Exploratory Radiomics Analysis on Digital Breast Tomosynthesis in Women With Mammographically Negative Dense Breasts,” Breast (Edinburgh, Scotland) 40 (2018): 92–96. 29723697 10.1016/j.breast.2018.04.016

[263] R. Fusco , P. Vallone , S. Filice , et al., “Radiomic Features Analysis by Digital Breast Tomosynthesis and Contrast‐enhanced Dual‐energy Mammography to Detect Malignant Breast Lesions,” Biomedical Signal Processing and Control 53 (2019): 101568.

[264] G. Murtaza , L. Shuib , A. W. Abdul Wahab , et al., “Deep Learning‐based Breast Cancer Classification Through Medical Imaging Modalities: State of the Art and Research Challenges,” Artificial Intelligence Review 53 (2020): 1655–1720.

[265] A. Sarno , G. Mettivier , F. di Franco , et al., “Dataset of Patient‐derived Digital Breast Phantoms for in Silico Studies in Breast Computed Tomography, Digital Breast Tomosynthesis, and Digital Mammography,” Medical Physics 48, no. 5 (2021): 2682–2693. 33683711 10.1002/mp.14826

[266] K. Dembrower , P. Lindholm , and F. Strand , “A Multi‐million Mammography Image Dataset and Population‐Based Screening Cohort for the Training and Evaluation of Deep Neural Networks‐the Cohort of Screen‐Aged Women (CSAW),” Journal of Digital Imaging 33, no. 2 (2020): 408–413. 31520277 10.1007/s10278-019-00278-0 PMC7165146

[267] O. M. Velarde , C. Lin , S. Eskreis‐Winkler , and L. C. Parra , “Robustness of Deep Networks for Mammography: Replication across Public Datasets,” Journal of Imaging Informatics in Medicine 37, no. 2 (2024): 536–546. 38343223 10.1007/s10278-023-00943-5 PMC11031505

[268] M. Muštra and A. Štajduhar , “Segmentation Masks for the Mini‐mammographic Image analysis society (mini‐MIAS) Database,” IEEE Consumer Electronics Magazine 9, no. 5 (2020): 28–33.

[269] H. Chougrad , H. Zouaki , and O. Alheyane , “Deep Convolutional Neural Networks for Breast Cancer Screening,” Computer Methods and Programs in Biomedicine 157 (2018): 19–30. 29477427 10.1016/j.cmpb.2018.01.011

[270] P. Murty , C. Anuradha , P. A. Naidu , et al., “Integrative Hybrid Deep Learning for Enhanced Breast Cancer Diagnosis: Leveraging the Wisconsin Breast Cancer Database and the CBIS‐DDSM Dataset,” Scientific Reports 14, no. 1 (2024): 26287. 39487199 10.1038/s41598-024-74305-8 PMC11530441

[271] X. Yu , Q. Zhou , S. Wang , and Y. D. Zhang , “A Systematic Survey of Deep Learning in Breast Cancer,” International Journal of Intelligent Systems 37, no. 1 (2022): 152–216.

[272] P. Oza , U. Oza , R. Oza , et al., “Digital Mammography Dataset for Breast Cancer Diagnosis Research (DMID) With Breast Mass Segmentation Analysis,” Biomedical Engineering Letters 14, no. 2 (2024): 317–330. 38374902 10.1007/s13534-023-00339-y PMC10874363

[273] H. T. Nguyen , H. Q. Nguyen , H. H. Pham , et al., “VinDr‐Mammo: A Large‐scale Benchmark Dataset for Computer‐aided Diagnosis in Full‐field Digital Mammography,” Scientific Data 10, no. 1 (2023): 277. 37173336 10.1038/s41597-023-02100-7 PMC10182079

[274] H. Koziolek , S. Grüner , R. Hark , V. Ashiwal , S. Linsbauer , and N. Eskandani , “LLM‐based and Retrieval‐Augmented Control Code Generation,” In: Proceedings of the 1st International Workshop on Large Language Models for Code . Association for Computing Machinery; 2024:22–29.

[275] B. B. Zimmermann , B. Deng , B. Singh , et al., “Multimodal Breast Cancer Imaging Using Coregistered Dynamic Diffuse Optical Tomography and Digital Breast Tomosynthesis,” Journal of Biomedial Optics 22, no. 4 (2017): 46008. 10.1117/1.JBO.22.4.046008 PMC5406652 28447102

[276] B. L. Sprague , R. Y. Coley , K. P. Lowry , et al., “Digital Breast Tomosynthesis versus Digital Mammography Screening Performance on Successive Screening Rounds From the Breast Cancer Surveillance Consortium,” Radiology 307, no. 5 (2023): e223142. 37249433 10.1148/radiol.223142 PMC10315524

[277] O. Imane , A. Mohamed , R. F. Lazhar , S. Hama , B. Elhadj , and A. Conci , “LAMIS‐DMDB: A New Full Field Digital Mammography Database for Breast Cancer AI‐CAD Researches,” Biomedical Signal Processing and Control 90 (2024): 105823.

[278] J. Park , J. Chłędowski , S. Jastrzębski , et al., “An Efficient Deep Neural Network to Classify Large 3D Images With Small Objects,” Ieee Transactions on Medical Imaging 43, no. 1 (2023): 351–365. 10.1109/TMI.2023.3302799 PMC11449265 37590109

[279] A. C. Pujara , J. Hui , and L. C. Wang , “Architectural Distortion in the Era of Digital Breast Tomosynthesis: Outcomes and Implications for Management,” Clinical Imaging 54 (2019): 133–137. 30639524 10.1016/j.clinimag.2019.01.004

[280] O. N. Oyelade , A. E. Ezugwu , M. S. Almutairi , A. K. Saha , L. Abualigah , and H. Chiroma , “A Generative Adversarial Network for Synthetization of Regions of Interest Based on Digital Mammograms,” Scientific Reports 12, no. 1 (2022): 6166. 35418566 10.1038/s41598-022-09929-9 PMC9008034

[281] J. R. Burt , N. Torosdagli , N. Khosravan , et al., “Deep Learning Beyond Cats and Dogs: Recent Advances in Diagnosing Breast Cancer With Deep Neural Networks,” British Journal of Radiology 91, no. 1089 (2018): 20170545. 29565644 10.1259/bjr.20170545 PMC6223155

[282] T. Viriyasaranon , J. W. Chun , Y. H. Koh , et al., “Annotation‐Efficient Deep Learning Model for Pancreatic Cancer Diagnosis and Classification Using CT Images: A Retrospective Diagnostic Study,” Cancers (Basel) 15, no. 13 (2023): 3392. 37444502 10.3390/cancers15133392 PMC10340780

[283] P. Oza , P. Sharma , S. Patel , and P. Kumar , “Computer‐aided Breast Cancer Diagnosis: Comparative Analysis of Breast Imaging Modalities and Mammogram Repositories,” Current Medical Imaging Reviews 19, no. 5 (2023): 456–468. 10.2174/1573405618666220621123156 35726812

[284] A. S. Betancourt Tarifa , C. Marrocco , M. Molinara , F. Tortorella , and A. Bria , “Transformer‐based Mass Detection in Digital Mammograms,” Journal of Ambient Intelligence and Humanized Computing 14, no. 3 (2023): 2723–2737.

[285] M. D. Halling‐Brown , L. M. Warren , D. Ward , et al., “OPTIMAM Mammography Image Database: A Large‐Scale Resource of Mammography Images and Clinical Data,” Radiology: Artificial Intelligence 3, no. 1 (2021): e200103. 33937853 10.1148/ryai.2020200103 PMC8082293

[286] M. M. Najafabadi , F. Villanustre , T. M. Khoshgoftaar , N. Seliya , R. Wald , and E. Muharemagic , “Deep Learning Applications and Challenges in Big Data Analytics,” Journal of Big Data 2 (2015): 1–21.

[287] W. Lotter , A. R. Diab , B. Haslam , et al., “Robust Breast Cancer Detection in Mammography and Digital Breast Tomosynthesis Using an Annotation‐efficient Deep Learning Approach,” Nature Medicine 27, no. 2 (2021): 244–249. 10.1038/s41591-020-01174-9 PMC9426656 33432172

[288] L. Balkenende , J. Teuwen , and R. M. Mann , “Application of Deep Learning in Breast Cancer imaging,” Seminars in Nuclear Medicine. (Elsevier, 2022): 584–596. 10.1053/j.semnuclmed.2022.02.003 35339259

[289] M. Yousefi , A. Krzyzak , and C. Y. Suen , “Mass Detection in Digital Breast Tomosynthesis Data Using Convolutional Neural Networks and Multiple Instance Learning,” Computers in Biology and Medicine 96 (2018): 283–293. 29665537 10.1016/j.compbiomed.2018.04.004

[290] D. Petrov , N. Marshall , K. Young , G. Zhang , and H. Bosmans , “Model and human Observer Reproducibility for Detection of Microcalcification Clusters in Digital Breast Tomosynthesis Images of Three‐dimensionally Structured Test Object,” Journal of Medical Imaging (Bellingham) 6, no. 1 (2019): 015503. 10.1117/1.JMI.6.1.015503 PMC6430963 30915383

[291] N. Houssami , K. Lång , S. Hofvind , et al., “Effectiveness of Digital Breast Tomosynthesis (3D‐mammography) in Population Breast Cancer Screening: A Protocol for a Collaborative Individual Participant Data (IPD) Meta‐analysis,” Translational Cancer Research 6, no. 4 (2017): 869–877.

[292] C. Reis , A. Pascoal , T. Sakellaris , and M. Koutalonis , “Quality Assurance and Quality Control in Mammography: A Review of Available Guidance Worldwide,” Insights Imaging 4, no. 5 (2013): 539–553. 23912879 10.1007/s13244-013-0269-1 PMC3781250

[293] C. Hill and L. Robinson , “Mammography Image Assessment; Validity and Reliability of Current Scheme,” Radiography 21, no. 4 (2015): 304–307.

[294] E. Goceri , “Medical Image Data Augmentation: Techniques, Comparisons and Interpretations,” Artificial Intelligence Review 56, no. 11 (2023): 1–45. 10.1007/s10462-023-10453-z PMC10027281 37362888

[295] Q. Zheng , M. Yang , X. Tian , N. Jiang , and D. Wang , “A Full Stage Data Augmentation Method in Deep Convolutional Neural Network for Natural Image Classification,” Discrete Dynamics in Nature and Society 2020, no. 1 (2020): 4706576.

[296] L. Taylor and G. Nitschke , “Improving Deep Learning With Generic Data Augmentation,” In: 2018 IEEE symposium series on computational intelligence (SSCI) . IEEE; 2018:1542–1547.

[297] A. J. Plompen , O. Cabellos , C. De Saint Jean , et al., “The Joint Evaluated Fission and Fusion Nuclear Data Library, JEFF‐3.3,” European Physical Journal A: Hadrons and Nuclei 56 (2020): 1–108.

[298] L. Garrucho , K. Kushibar , R. Osuala , et al., “High‐resolution Synthesis of High‐density Breast Mammograms: Application to Improved Fairness in Deep Learning Based Mass Detection,” Frontiers in oncology 12 (2022): 1044496. 36755853 10.3389/fonc.2022.1044496 PMC9899892

[299] J. G. Elmore and C. I. Lee , “Data Quality, Data Sharing, and Moving Artificial Intelligence Forward,” JAMA Network Open 4, no. 8 (2021): e2119345. 34398208 10.1001/jamanetworkopen.2021.19345 PMC8612009

[300] G. A. Kaissis , M. R. Makowski , D. Rückert , and R. F. Braren , “Secure, Privacy‐preserving and Federated Machine Learning in Medical Imaging,” Nature Machine Intelligence 2, no. 6 (2020): 305–311.

[301] M. Field , D. I. Thwaites , M. Carolan , et al., “Infrastructure Platform for Privacy‐preserving Distributed Machine Learning Development of Computer‐assisted Theragnostics in Cancer,” Journal of Biomedical Informatics 134 (2022): 104181. 36055639 10.1016/j.jbi.2022.104181

[302] F. Cossio , H. Schurz , M. Engstrom , et al., “VAI‐B: A Multicenter Platform for the External Validation of Artificial Intelligence Algorithms in Breast Imaging,” J Med Imaging (Bellingham) 10, no. 6 (2023): 061404. 36949901 10.1117/1.JMI.10.6.061404 PMC10026999

[303] X. Liu , L. Xie , Y. Wang , et al., “Privacy and Security Issues in Deep Learning: A Survey,” IEEE Access 9 (2020): 4566–4593.

[304] Y. Chen , X. Qin , J. Wang , C. Yu , and W. Gao , “Fedhealth: A Federated Transfer Learning Framework for Wearable Healthcare,” Ieee Intelligent Systems 35, no. 4 (2020): 83–93.

[305] G. Dhiman , S. Juneja , H. Mohafez , et al., “Federated Learning Approach to Protect Healthcare Data Over Big Data Scenario,” Sustainability 14, no. 5 (2022): 2500.

[306] Q. Yang , Y. Liu , T. Chen , and Y. Tong , “Federated Machine Learning: Concept and Applications,” ACM Transactions on Intelligent Systems and Technology 10, no. 2 (2019): 1–19.

[307] Y. Chen , J. Li , F. Wang , et al., “DS2PM: A Data‐sharing Privacy Protection Model Based on Blockchain and Federated Learning,” IEEE Internet of Things Journal 10, no. 14 (2021): 12112–12125.

[308] M. J. Sheller , G. A. Reina , B. Edwards , J. Martin , and S. Bakas , “Multi‐institutional Deep Learning Modeling Without Sharing Patient Data: A Feasibility Study on Brain Tumor Segmentation,” In: Brainlesion: Glioma, Multiple Sclerosis, Stroke and Traumatic Brain Injuries: 4th International Workshop, BrainLes 2018, Held in Conjunction with MICCAI 2018, Granada, Spain, September 16, 2018, Revised Selected Papers, Part I 4 . Springer; 2019:92–104. 10.1007/978-3-030-11723-8_9 PMC6589345 31231720

[309] R. Yan , F. Zhang , X. Rao , et al., “Richer Fusion Network for Breast Cancer Classification Based on Multimodal Data,” BMC Medical Informatics and Decision Making [Electronic Resource] 21, no. Suppl1 (2021): 134. 33888098 10.1186/s12911-020-01340-6 PMC8061018

[310] F.‐Z. Nakach , A. Idri , and E. Goceri , “A Comprehensive Investigation of Multimodal Deep Learning Fusion Strategies for Breast Cancer Classification,” Artificial Intelligence Review 57, no. 12 (2024): 1–53.

[311] S. Steyaert , M. Pizurica , D. Nagaraj , et al., “Multimodal Data Fusion for Cancer Biomarker Discovery With Deep Learning,” Nature Machine Intelligence 5, no. 4 (2023): 351–362. 10.1038/s42256-023-00633-5 PMC10484010 37693852

[312] W. A. Berg , L. Gutierrez , M. S. NessAiver , et al., “Diagnostic Accuracy of Mammography, Clinical Examination, US, and MR Imaging in Preoperative Assessment of Breast Cancer,” Radiology 233, no. 3 (2004): 830–849. 15486214 10.1148/radiol.2333031484

[313] L. A Carbonaro . Clinical Applications for Digital Breast Tomosynthesis. In: A. Tagliafico , N. Houssami , M. Calabrese , eds. “Digital Breast Tomosynthesis: A Practical Approach”. (Springer, 2016): 45–58.

[314] A. Rodriguez‐Ruiz , E. Krupinski , J. J. Mordang , et al., “Detection of Breast Cancer With Mammography: Effect of an Artificial Intelligence Support System,” Radiology 290, no. 2 (2019): 305–314. 30457482 10.1148/radiol.2018181371

[315] J. Dong , Y. Geng , D. Lu , et al., “Clinical Trials for Artificial Intelligence in Cancer Diagnosis: A Cross‐Sectional Study of Registered Trials in ClinicalTrials.Gov,” Frontiers in oncology 10 (2020): 1629. 33042806 10.3389/fonc.2020.01629 PMC7522504

[316] B. Abhisheka , S. K. Biswas , and B. Purkayastha , “A Comprehensive Review on Breast Cancer Detection, Classification and Segmentation Using Deep Learning,” Archives of Computational Methods in Engineering 30, no. 8 (2023): 5023–5052.

[317] S. Ramesh , S. Sasikala , S. Gomathi , V. Geetha , and V. Anbumani , “Segmentation and Classification of Breast Cancer Using Novel Deep Learning Architecture,” Neural Computing and Applications 34, no. 19 (2022): 16533–16545.

[318] J. Zhu , J. Geng , W. Shan , et al., “Development and Validation of a Deep Learning Model for Breast Lesion Segmentation and Characterization in Multiparametric MRI,” Frontiers in oncology 12 (2022): 946580. 36033449 10.3389/fonc.2022.946580 PMC9402900

[319] R. Azad , M. Heidari , M. Shariatnia , et al., “Transdeeplab: Convolution‐free Transformer‐based Deeplab v3+ for Medical Image segmentation,” International Workshop on PRedictive Intelligence in MEdicine. (Springer, 2022): 91–102.

[320] H. Hui , X. Zhang , F. Li , X. Mei , and Y. Guo , “A Partitioning‐stacking Prediction Fusion Network Based on an Improved Attention U‐Net for Stroke Lesion Segmentation,” IEEE Access 8 (2020): 47419–47432.

[321] W. C. Shia , F. R. Hsu , S. T. Dai , S. L. Guo , and D. R. Chen , “Semantic Segmentation of the Malignant Breast Imaging Reporting and Data System Lexicon on Breast Ultrasound Images by Using DeepLab v3,” Sensors (Basel) 22, no. 14 (2022): 5352. 35891030 10.3390/s22145352 PMC9323504

[322] T. Alam , W. C. Shia , F. R. Hsu , and T. Hassan , “Improving Breast Cancer Detection and Diagnosis Through Semantic Segmentation Using the Unet3+ Deep Learning Framework,” Biomedicines 11, no. 6 (2023): 1536. 37371631 10.3390/biomedicines11061536 PMC10294974

[323] J. Li , L. Cheng , T. Xia , H. Ni , and J. Li , “Multi‐scale Fusion U‐net for the Segmentation of Breast Lesions,” IEEE Access 9 (2021): 137125–137139.

[324] M. Bobowicz , M. Rygusik , J. Buler , et al., “Attention‐Based Deep Learning System for Classification of Breast Lesions‐Multimodal, Weakly Supervised Approach,” Cancers (Basel) 15, no. 10 (2023): 2704. 37345041 10.3390/cancers15102704 PMC10216803

[325] T. Cogan , M. Cogan , and L. Tamil , “RAMS: Remote and Automatic Mammogram Screening,” Computers in Biology and Medicine 107 (2019): 18–29. 30771549 10.1016/j.compbiomed.2019.01.024

[326] K. Balaji , “Image Augmentation Based on Variational Autoencoder for Breast Tumor Segmentation,” Academic Radiology 30, no. Suppl2 (2023): S172–S183. 36804294 10.1016/j.acra.2022.12.035

[327] L. Luo , X. Wang , Y. Lin , et al., “Deep Learning in Breast Cancer Imaging: A Decade of Progress and Future Directions,” Ieee Reviews in Biomedical Engineering 18 (2024): 130–151. 10.1109/RBME.2024.3357877 38265911

[328] X. Wang , Z. Li , X. Luo , et al., “Black‐box Domain Adaptative Cell Segmentation via Multi‐source Distillation,” In: International Conference on Medical Image Computing and Computer‐Assisted Intervention . Springer; 2023:749–758.

[329] C. Chen , W. Xie , Y. Wen , Y. Huang , and X. Ding , “Multiple‐source Domain Adaptation With Generative Adversarial Nets,” Knowledge‐Based Systems 199 (2020): 105962.

[330] L. Garrucho , K. Kushibar , S. Jouide , O. Diaz , L. Igual , and K. Lekadir , “Domain Generalization in Deep Learning Based Mass Detection in Mammography: A Large‐scale Multi‐center Study,” Artificial Intelligence in Medicine 132 (2022): 102386. 36207090 10.1016/j.artmed.2022.102386

[331] G. Kang , L. Jiang , Y. Wei , Y. Yang , and A. Hauptmann , “Contrastive Adaptation Network for Single‐and Multi‐source Domain Adaptation,” Ieee Transactions on Pattern Analysis and Machine Intelligence 44, no. 4 (2020): 1793–1804. 10.1109/TPAMI.2020.3029948 33035160

[332] K. Li , J. Lu , H. Zuo , and G. Zhang , “Multi‐source Contribution Learning for Domain Adaptation,” IEEE Transactions on Neural Networks and Learning Systems 33, no. 10 (2021): 5293–5307. 10.1109/TNNLS.2021.3069982 33835927

[333] Q. Wu , X. Zhou , Y. Yan , H. Wu , and H. Min , “Online Transfer Learning by Leveraging Multiple Source Domains,” Knowledge and Information Systems 52 (2017): 687–707.

[334] A. S. Morcos , D. G. Barrett , N. C. Rabinowitz , and M. Botvinick . “On the Importance of Single Directions for Generalization,” *arXiv preprint arXiv:180306959* . 2018.

[335] I. Salehin and D.‐K. Kang , “A Review on Dropout Regularization Approaches for Deep Neural Networks Within the Scholarly Domain,” Electronics 12, no. 14 (2023): 3106.

[336] S. H. Khan , M. Hayat , and F. Porikli , “Regularization of Deep Neural Networks With Spectral Dropout,” Neural Networks 110 (2019): 82–90. 30504041 10.1016/j.neunet.2018.09.009

[337] Y. Ma , Q. Yan , Y. Liu , J. Liu , J. Zhang , and Y. Zhao , “StruNet: Perceptual and Low‐rank Regularized Transformer for Medical Image Denoising,” Medical Physics 50, no. 12 (2023): 7654–7669. 37278312 10.1002/mp.16550

[338] Z. Xiao , Y. Su , Z. Deng , and W. Zhang , “Efficient Combination of CNN and Transformer for Dual‐Teacher Uncertainty‐guided Semi‐supervised Medical Image Segmentation,” Computer Methods and Programs in Biomedicine 226 (2022): 107099. 36116398 10.1016/j.cmpb.2022.107099

[339] S. Aslani , M. Dayan , L. Storelli , et al., “Multi‐branch Convolutional Neural Network for Multiple Sclerosis Lesion Segmentation,” Neuroimage 196 (2019): 1–15. 30953833 10.1016/j.neuroimage.2019.03.068

[340] C. Sendra‐Balcells , V. M. Campello , C. Martin‐Isla , et al., “Domain Generalization in Deep Learning for Contrast‐enhanced Imaging,” Computers in Biology and Medicine 149 (2022): 106052. 36055164 10.1016/j.compbiomed.2022.106052

[341] J. Wang , C. Lan , C. Liu , et al., “Generalizing to Unseen Domains: A Survey on Domain Generalization,” Ieee Transactions on Knowledge and Data Engineering 35, no. 8 (2022): 8052–8072.

[342] A. J. Thirunavukarasu , D. S. J. Ting , K. Elangovan , L. Gutierrez , T. F. Tan , and D. S. W. Ting , “Large Language Models in Medicine,” Nature Medicine 29, no. 8 (2023): 1930–1940. 10.1038/s41591-023-02448-8 37460753

[343] E. Kasneci , K. Seßler , S. Küchemann , et al., “ChatGPT for Good? On Opportunities and Challenges of Large Language Models for Education,” Learning and Individual Differences 103 (2023): 102274.

[344] L. Wang , C. Ma , X. Feng , et al., “A Survey on Large Language Model Based Autonomous Agents,” Frontiers of Computer Science 18, no. 6 (2024): 186345.

[345] Y. Liu , T. Han , S. Ma , et al., “Summary of Chatgpt‐related Research and Perspective towards the Future of Large Language Models,” Meta‐Radiology 1, no. 2 (2023): 100017.

[346] K. Huang , Y. Qu , H. Cousins , et al., “Crispr‐GPT: An LLM Agent for Automated Design of Gene‐editing Experiments,” arXiv preprint arXiv:240418021. 2024.

[347] Q. Jin , Z. Wang , C. S. Floudas , et al., “Matching Patients to Clinical Trials With Large Language Models,” Nature Communications 15, no. 1 (2024): 9074. 10.1038/s41467-024-53081-z PMC11574183 39557832

[348] S. Jabbour , D. Fouhey , E. Kazerooni , J. Wiens , and M. W. Sjoding , “Combining Chest X‐rays and Electronic Health Record (EHR) Data Using Machine Learning to Diagnose Acute respiratory Failure,” Journal of the American Medical Informatics Association 29, no. 6 (2022): 1060–1068. 35271711 10.1093/jamia/ocac030 PMC9093032

[349] X. Yang , A. Chen , N. PourNejatian , et al., “A Large Language Model for Electronic Health Records,” NPJ Digital Medicine 5, no. 1 (2022): 194. 36572766 10.1038/s41746-022-00742-2 PMC9792464

[350] M. Wornow , Y. Xu , R. Thapa , et al., “The Shaky Foundations of Large Language Models and Foundation Models for Electronic Health Records,” NPJ Digital Medicine 6, no. 1 (2023): 135. 37516790 10.1038/s41746-023-00879-8 PMC10387101

[351] M. Guevara , S. Chen , S. Thomas , et al., “Large Language Models to Identify Social Determinants of Health in Electronic Health Records,” NPJ Digital Medicine 7, no. 1 (2024): 6. 38200151 10.1038/s41746-023-00970-0 PMC10781957

[352] V. Lievin , C. E. Hother , A. G. Motzfeldt , and O. Winther , “Can Large Language Models Reason About Medical Questions?,” Patterns (N Y) 5, no. 3 (2024): 100943. 38487804 10.1016/j.patter.2024.100943 PMC10935498

[353] H. Huang , O. Zheng , D. Wang , et al., “ChatGPT for Shaping the Future of Dentistry: The Potential of Multi‐modal Large Language Model,” International Journal of Oral Science 15, no. 1 (2023): 29. 37507396 10.1038/s41368-023-00239-y PMC10382494

[354] Z. Tan , M. Yang , L. Qin , et al., “An Empirical Study and Analysis of Text‐to‐image Generation Using Large Language Model‐powered Textual Representation,” In: European Conference on Computer Vision . Springer; 2025:472–489.

[355] Y. Guo , W. Qiu , G. Leroy , S. Wang , and T. Cohen , “Retrieval Augmentation of Large Language Models for Lay Language Generation,” Journal of Biomedical Informatics 149 (2024): 104580. 38163514 10.1016/j.jbi.2023.104580 PMC10874606

[356] J. J. Woo , A. J. Yang , R. J. Olsen , et al., “Custom Large Language Models Improve Accuracy: Comparing Retrieval Augmented Generation and Artificial Intelligence Agents to Non‐custom Models for Evidence‐based Medicine,” Arthroscopy 41, no. 3 (2024): 565–573. e6. 39521391 10.1016/j.arthro.2024.10.042

[357] M. Ryspayeva , M. Molinara , A. Bria , C. Marrocco , and F. Tortorella , “Transfer Learning in Breast Mass Detection on the OMI‐DB Dataset: A Preliminary Study,” Pattern Recognition, Computer Vision, and Image Processing ICPR 2022 International Workshops and Challenges. (Springer Nature Switzerland, 2023): 529–538.

[358] M. Jeong , J. Sohn , M. Sung , and J. Kang , “Improving Medical Reasoning Through Retrieval and Self‐reflection With Retrieval‐augmented Large Language Models,” Bioinformatics 40, no. Suppl1 (2024): i119–i129. 38940167 10.1093/bioinformatics/btae238 PMC11211826

[359] A. Cozzi , K. Pinker , A. Hidber , et al., “BI‐RADS Category Assignments by GPT‐3.5, GPT‐4, and Google Bard: A Multilanguage Study,” Radiology 311, no. 1 (2024): e232133. 38687216 10.1148/radiol.232133 PMC11070611

[360] V. Sorin , B. S. Glicksberg , Y. Artsi , et al., “Utilizing Large Language Models in Breast Cancer Management: Systematic Review,” Journal of Cancer Research and Clinical Oncology 150, no. 3 (2024): 140. 38504034 10.1007/s00432-024-05678-6 PMC10950983

[361] A. Rao , J. Kim , M. Kamineni , et al., “Evaluating GPT as an Adjunct for Radiologic Decision Making: GPT‐4 versus GPT‐3.5 in a Breast Imaging Pilot,” Journal of the American College of Radiology 20, no. 10 (2023): 990–997. 37356806 10.1016/j.jacr.2023.05.003 PMC10733745

[362] R. Bhayana , “Chatbots and Large Language Models in Radiology: A Practical Primer for Clinical and Research Applications,” Radiology 310, no. 1 (2024): e232756. 38226883 10.1148/radiol.232756

[363] S. Pan , L. Luo , Y. Wang , C. Chen , J. Wang , and X. Wu , “Unifying Large Language Models and Knowledge Graphs: A Roadmap,” Ieee Transactions on Knowledge and Data Engineering 36, no. 7 (2024): 3580–3599.

[364] T. Guo , Q. Yang , C. Wang , et al., “Knowledgenavigator: Leveraging Large Language Models for Enhanced Reasoning Over Knowledge Graph,” Complex Intell Syst 10, no. 5 (2024): 7063–7076.

[365] Y. Hu , F. Zou , J. Han , X. Sun , and Y. Wang , “Llm‐tikg: Threat Intelligence Knowledge Graph Construction Utilizing Large Language Model,” Computers and Security 145 (2024): 103999.

[366] A. Fang , P. Lou , J. Hu , et al., “Head and Tail Entity Fusion Model in Medical Knowledge Graph Construction: Case Study for Pituitary Adenoma,” JMIR medical informatics Med Inform 9, no. 7 (2021): e28218. 10.2196/28218 PMC8367125 34057414

[367] Z. Zhang , L. Cao , X. Chen , W. Tang , Z. Xu , and Y. Meng , “Representation Learning of Knowledge Graphs With Entity Attributes,” IEEE Access 8 (2020): 7435–7441.

[368] X. Huang , J. Tang , Z. Tan , W. Zeng , J. Wang , and X. Zhao , “Knowledge Graph Embedding by Relational and Entity Rotation,” Knowledge‐Based Systems 229 (2021): 107310.

[369] S. M. S. Hasan , D. Rivera , X. C. Wu , E. B. Durbin , J. B. Christian , and G. Tourassi , “Knowledge Graph‐Enabled Cancer Data Analytics,” IEEE Journal of Biomedical and Health Informatics 24, no. 7 (2020): 1952–1967. 32386166 10.1109/JBHI.2020.2990797 PMC8324069

[370] X. Cao and Y. Liu , “Relmkg: Reasoning With Pre‐trained Language Models and Knowledge Graphs for Complex Question Answering,” Applied Intelligence 53, no. 10 (2023): 12032–12046.

[371] X. Li , A. Henriksson , M. Duneld , J. Nouri , and Y. Wu , “Evaluating Embeddings From Pre‐trained Language Models and Knowledge Graphs for Educational Content Recommendation,” Future Internet 16, no. 1 (2023): 12.

[372] X. Li , S. Sun , T. Tang , et al., “Construction of a Knowledge Graph for Breast Cancer Diagnosis Based on Chinese Electronic Medical Records: Development and Usability Study,” BMC Medical Informatics and Decision Making [Electronic Resource] 23, no. 1 (2023): 210. 37817193 10.1186/s12911-023-02322-0 PMC10563203

[373] C. Zhang and X. Cao , “Biological Gene Extraction Path Based on Knowledge Graph and Natural Language Processing,” Frontiers in Genetics 13 (2022): 1086379. 36712855 10.3389/fgene.2022.1086379 PMC9880067

[374] C. Wang , Y. Chen , F. Liu , et al., “An Interpretable and Accurate Deep‐learning Diagnosis Framework Modelled With Fully and Semi‐supervised Reciprocal Learning,” Ieee Transactions on Medical Imaging 43, no. 1 (2023): 392–404. 10.1109/TMI.2023.3306781 37603481

[375] S. T. Kim , H. Lee , H. G. Kim , and Y. M Ro , “ICADx: Interpretable Computer Aided Diagnosis of Breast Masses,” In: Medical Imaging 2018: Computer‐Aided Diagnosis . SPIE; 2018:450–459.

[376] S. T. Kim , J. H. Lee , H. Lee , and Y. M. Ro , “Visually Interpretable Deep Network for Diagnosis of Breast Masses on Mammograms,” Physics in Medicine and Biology 63, no. 23 (2018): 235025. 30511660 10.1088/1361-6560/aaef0a

[377] D. Castelvecchi , “Can We Open the Black Box of AI?,” Nature 538, no. 7623 (2016): 20–23. 27708329 10.1038/538020a

[378] R. Geirhos , J.‐H. Jacobsen , C. Michaelis , et al., “Shortcut Learning in Deep Neural Networks,” Nature Machine Intelligence 2, no. 11 (2020): 665–673.

[379] K. Freeman , J. Geppert , C. Stinton , et al., “Use of Artificial Intelligence for Image Analysis in Breast Cancer Screening Programmes: Systematic Review of Test Accuracy,” Bmj 374 (2021): n1872. 34470740 10.1136/bmj.n1872 PMC8409323

[380] T. Ching , D. S. Himmelstein , B. K. Beaulieu‐Jones , et al., “Opportunities and Obstacles for Deep Learning in Biology and Medicine,” Journal of the Royal Society, Interface 15, no. 141 (2018): 20170387. 29618526 10.1098/rsif.2017.0387 PMC5938574

[381] W. Samek , G. Montavon , A. Vedaldi , L. K. Hansen , and K. R. Müller , Explainable Ai–preface. Explainable AI: Interpreting, Explaining and Visualizing Deep Learning. (Springer Nature, 2019): v–vii.

[382] V. Beaudouin , I. Bloch , D. Bounie , et al., “Flexible and Context‐specific AI Explainability: A Multidisciplinary Approach,” arXiv preprint arXiv:200307703. 2020.

[383] S. Mohseni , N. Zarei , and E. D. Ragan , “A Multidisciplinary Survey and Framework for Design and Evaluation of Explainable AI Systems,” ACM Transactions on Interactive Intelligent Systems 11, no. 3‐4 (2021): 1–45.

[384] L. Farah , J. M. Murris , I. Borget , A. Guilloux , N. M. Martelli , and S. I. M. Katsahian , “Assessment of Performance, Interpretability, and Explainability in Artificial Intelligence‐Based Health Technologies: What Healthcare Stakeholders Need to Know,” Mayo Clinic Proceedings: Digital Health 1, no. 2 (2023): 120–138. 40206724 10.1016/j.mcpdig.2023.02.004 PMC11975643

[385] S. Chakraborty , R. Tomsett , R. Raghavendra , et al., “Interpretability of Deep Learning Models: A Survey of Results,” In: 2017 IEEE smartworld, ubiquitous intelligence & computing, advanced & trusted computed, scalable computing & communications, cloud & big data computing, Internet of people and smart city innovation (smartworld/SCALCOM/UIC/ATC/CBDcom/IOP/SCI) . IEEE; 2017:1–6.

[386] Y. Lu , T. Chen , N. Hao , C. Van Rechem , J. Chen , and T. Fu , “Uncertainty Quantification and Interpretability for Clinical Trial Approval Prediction,” Health Data Science 4 (2024): 0126. 38645573 10.34133/hds.0126 PMC11031120

[387] H. Panwar , P. K. Gupta , M. K. Siddiqui , R. Morales‐Menendez , P. Bhardwaj , and V. Singh , “A Deep Learning and Grad‐CAM Based Color Visualization Approach for Fast Detection of COVID‐19 Cases Using Chest X‐ray and CT‐Scan Images,” Chaos, Solitons & Fractals 140 (2020): 110190. 32836918 10.1016/j.chaos.2020.110190 PMC7413068

[388] Y. Zhong , Y. Piao , and G. Zhang , “Multi‐view Fusion‐based Local‐global Dynamic Pyramid Convolutional Cross‐tansformer Network for Density Classification in Mammography,” Physics in Medicine and Biology 68, no. 22 (2023): 225012. 10.1088/1361-6560/ad02d7 37827166

[389] Y. Gal and Z. Ghahramani , “Dropout as a Bayesian Approximation: Representing Model Uncertainty in Deep Learning,” In: international conference on machine learning . PMLR; 2016:1050–1059.

[390] S. Walia , K. Kumar , S. Agarwal , and H. Kim , “Using xai for Deep Learning‐based Image Manipulation Detection With shapley Additive Explanation,” Symmetry 14, no. 8 (2022): 1611.

[391] P. Guleria , P. N. Srinivasu , and M. Hassaballah , “Diabetes Prediction Using Shapley Additive Explanations and DSaaS Over Machine Learning Classifiers: A Novel Healthcare Paradigm,” Multimedia Tools and Applications 83, no. 14 (2024): 40677–40712.

[392] J. Li , Y. Zhang , S. He , and Y. Tang , “Interpretable Mortality Prediction Model for ICU Patients With Pneumonia: Using shapley Additive Explanation Method,” BMC Pulmonary Medicine 24, no. 1 (2024): 447. 39272037 10.1186/s12890-024-03252-x PMC11395639

[393] O. O. Oladimeji , H. Ayaz , I. McLoughlin , and S. Unnikrishnan , “Mutual Information‐based Radiomic Feature Selection With SHAP Explainability for Breast Cancer Diagnosis,” Results in Engineering 24 (2024): 103071.

[394] S. Shen , S. X. Han , D. R. Aberle , A. A. Bui , and W. Hsu , “An Interpretable Deep Hierarchical Semantic Convolutional Neural Network for Lung Nodule Malignancy Classification,” Expert Systems with Applications 128 (2019): 84–95. 31296975 10.1016/j.eswa.2019.01.048 PMC6623975

[395] A. A. Verma , J. Murray , R. Greiner , et al., “Implementing Machine Learning in Medicine,” Cmaj 193, no. 34 (2021): E1351–E1357. 35213323 10.1503/cmaj.202434 PMC8432320

[396] H. Bosmans and N. Marshall , “Radiation Doses and Risks Associated With Mammographic Screening,” Current Radiology Reports 1, no. 1 (2013): 30–38.

