# Supplementary material for: Deep Learning in Digital Breast Tomosynthesis: Current Status, Challenges, and Future Trends
Source: MedComm (2020). 2025 Jun 9;6(6):e70247. doi: 10.1002/mco2.70247 (PMC12146671; doi:10.1002/mco2.70247)
Supplement: Supplementary file 1 — Supporting Information [file MCO2-6-e70247-s001.docx]

**SUPPLEMENTAL MATERIAL**

**Deep Learning in Digital Breast Tomosynthesis: Current Status, Challenges and Future Trends**

Ruoyun Wang^1,#^, Fanxuan Chen^1,2,3,#^, Haoman Chen^1,2,3,#^, Chenxing Lin^3,#^, Jincen Shuai^4^, Yutong Wu^1^, Lixiang Ma^5^, Xiaoqu Hu^1^, Min Wu^2,6,7^, Jin Wang^2,8^, Qi Zhao^9,10,*^, Jianwei Shuai^2,3,10,*^, Jingye Pan^10,11,12*^.

**^1^**Wenzhou Medical University, Wenzhou, 325000, China

**^2^**Wenzhou Institute, University of Chinese Academy of Sciences, Wenzhou, 325000, China

**^3^**Oujiang Laboratory (Zhejiang Lab for Regenerative Medicine, Vision, and Brain Health), Wenzhou, 325000, China

**^4^**UCSC Baskin School of Engineering, University of California, Santa Cruz, 95054, USA

**^5^**Department of Anatomy, Histology & Embryology, School of Basic Medical Sciences, Fudan University, Shanghai, 200433, China

**^6^**Department of Medicine, Harvard Medical School, and Brigham and Women's Hospital, Boston, 02115, USA

**^7^**Joint Research Centre on Medicine, The Affiliated Xiangshan Hospital of Wenzhou Medical University, Ningbo, 315700, China

**^8^**Stony Brook University, Stony Brook, New York, 11794, USA

**^9^**School of Computer Science and Software Engineering, University of Science and Technology Liaoning, Anshan, 114051, China

**^10^**Key Laboratory of Intelligent Treatment and Life Support for Critical Diseases of Zhejiang Province, Wenzhou, 325000, China

**^11^**Department of Big Data in Health Science, The First Affiliated Hospital of Wenzhou Medical University, Wenzhou, 325000, China

**^12^**Zhejiang Engineering Research Center for Hospital Emergency and Process Digitization, Wenzhou, 325000, China

**^#^**These authors contributed equally to the paper as first authors.

*Corresponding author

**Email:** zhaoqi@lnu.edu.cn, shuaijw@wiucas.ac.cn, panjingye@wzhospital.ac.cn

**This file includes:**

Tables. S1

**Table S1. The summary of data privacy and sharing technologies.**

| **Method** | **Technology** | **Description** | **Advantages** | **Disadvantages** | **Links** |
| --- | --- | --- | --- | --- | --- |
| Data Preprocessing Techniques | Anonymization | Removes personal identifiers to protect privacy. | Allows data analysis without revealing personal information. | May reduce data usability and accuracy; complex algorithms can increase costs. | https://pubmed.ncbi.nlm.nih.gov/39018389/ |
|  | De-identification | Deletes direct identifiers to lower re-identification risks. | Facilitates data analysis while protecting privacy. | Still carries potential re-identification risks. | https://pubmed.ncbi.nlm.nih.gov/38414537/ |
|  | Data Masking | Alters sensitive data to reduce sensitivity while retaining usability. | Balances data usability and privacy. | May devalue original data; requires careful masking rules to prevent leaks. | https://pubmed.ncbi.nlm.nih.gov/24307745/ |
|  | Differential Privacy | Adds noise to queries to protect individual data points. | Provides strict privacy protection with quantifiable levels. | May increase data noise, affecting accuracy and usability. | https://pubmed.ncbi.nlm.nih.gov/34333623/ |
| Data Access Techniques | Attribute Based Access Control | Dynamically decides access rights based on attributes like department or role. | Highly flexible and adaptable to changes. | Complex policy design and management; increased system overhead. | https://pubmed.ncbi.nlm.nih.gov/37274839/ |
|  | Role-Based Access Control | Manages permissions through roles assigned to users. | Simplified management and consistency across roles. | Complex role design; less flexible for special needs; potential over-permissions. | https://pubmed.ncbi.nlm.nih.gov/34206164/ |
|  | Access Control List | Restricts data access based on user attributes. | Precise control over data access. | Complex permission management; increased system complexity. | <https://pubmed.ncbi.nlm.nih.gov/36298378/> |
|  | Application Programming Interface | Facilitates data exchange between systems with fine-grained access control. | Eases integration and maintenance across platforms. | Security risks; dependency issues. | <https://pubmed.ncbi.nlm.nih.gov/35773915/> |
|  | Searchable Encryption | Allows efficient and secure data retrieval while encrypted. | Prevents data interception and eavesdropping. | High performance overhead; complex key management. | https://pubmed.ncbi.nlm.nih.gov/30832294/ |
| Data Transmission Techniques | Secure File Transfer Protocol | Encrypts data in transit to ensure confidentiality and integrity. | Data encryption; identity verification; widely supported. | Performance overhead; complex configuration. | <https://pubmed.ncbi.nlm.nih.gov/12590154/> |
|  | Virtual Private Network | Securely connects remote users to internal networks via encrypted tunnels. | Remote access; data encryption; flexible encryption protocols. | Performance overhead; complex configuration. | <https://ieeexplore.ieee.org/document/7314859> |
|  | Key Encryption Algorithm | Includes symmetric, asymmetric, and digest algorithms for data encryption and decryption. | Ensures data confidentiality, integrity, authentication, and non-repudiation. | Computational complexity; key management challenges; performance impact. | <https://ieeexplore.ieee.org/document/7043532> |
| Data Sharing Techniques | Federated Learning | Allows model training across multiple data sources without sharing raw data. | Protects data privacy while enabling data sharing and model training. | Requires solving synchronization issues across devices and servers. | <https://pubmed.ncbi.nlm.nih.gov/35737624/> |
|  | Blockchain | Ensures data immutability and transparency through distributed ledgers. | High security and immutability; enhances trust in data sharing. | Increases system complexity and maintenance costs; high computational demands. | <https://pubmed.ncbi.nlm.nih.gov/35315778/> |
|  | Secure Multiparty Computation | Enables secure collaborative computations without revealing data to any party. | Supports secure data collaboration without privacy leakage. | Complex and inefficient implementation; requires advanced technical support. | <https://pubmed.ncbi.nlm.nih.gov/31950974/> |
|  | Homomorphic Encryption | Allows computations on encrypted data that yield the same results as on plaintext. | Enables computations while maintaining data privacy. | High computational complexity; lower efficiency due to encryption overhead. | <https://pubmed.ncbi.nlm.nih.gov/35531323/> |
|  | Data Sharing Platforms | Provides a secure platform for data sharing while protecting privacy, with features like data storage, access control, and usage tracking. | Promotes research, data reuse, collaboration, transparency, and standardization. | Requires ensuring privacy, managing data quality, access control, compliance, and maintenance costs. | <https://pubmed.ncbi.nlm.nih.gov/29241491/> |
